# Bdh1l Gene Expression Is a Potential Molecular Factor in the Evolution of Carotenoid‐Based Colour Diversity of Cichlid Fishes

**DOI:** 10.1111/mec.70065

**Published:** 2025-08-20

**Authors:** Pooja Singh, Angelika Ziegelbecker, Christoph Hahn, Walter Goessler, Ronald A. Glabonjat, Ehsan Pashay Ahi, Kristina M. Sefc

**Affiliations:** ^1^ Aquatic Ecology & Evolution Division, Institute of Ecology and Evolution University of Bern Bern Switzerland; ^2^ Department of Fish Ecology & Evolution Swiss Federal Institute of Aquatic Science and Technology (EAWAG) Kastanienbaum Switzerland; ^3^ Institute of Biology University of Graz Graz Austria; ^4^ Institute of Chemistry University of Graz Graz Austria; ^5^ Department of Environmental Health Science Columbia University Mailman School of Public Health New York USA; ^6^ Organismal and Evolutionary Biology Research Programme Faculty of Biological and Environmental Sciences, University of Helsinki Finland

**Keywords:** carotenoid metabolism, coloration, colour pattern evolution, comparative transcriptomics, diversification, fish

## Abstract

Carotenoids contribute substantially to animal body colour pattern diversity. While the ecological and evolutionary drivers of carotenoid coloration are reasonably well understood, the molecular mechanisms facilitating evolutionary transitions between red and yellow hues are less investigated. Here we leverage phylogenetically replicated red‐versus‐yellow colour contrasts in three pairs of closely related cichlid fishes (*Tropheus* and *Aulonocara*; Haplochromini) to investigate biochemical and genetic parallels in carotenoid colour differentiation. Red skin samples contained the ketocarotenoids rhodoxanthin, canthaxanthin, and astacene, the latter as likely saponification product of astaxanthin. A re‐analysis of existing RNA‐seq data using an improved bioinformatics pipeline identified consistent red‐versus‐yellow gene expression differences. Notably, transcripts of a gene coding for a 3‐hydroxybutyrate dehydrogenase type 1 enzyme (*bdh1l*) and further known carotenoid genes (*scarb1*, *bco2*, *ttc39b*) were significantly more abundant in red than in yellow skin tissue in all taxon pairs. Homologues of Bdh1l have recently been discovered to mediate C4‐ketocarotenoid biosynthesis in birds and fish, but only in the presence of a cytochrome P450 enzyme. We found no consistent differences in cytochrome P450 gene expression. Our results suggest that *bdh1l* expression regulation might operate as a molecular switch for C4‐ketocarotenoid biosynthesis and colour pattern differentiation in different radiations of cichlid fish, apparently in the presence of a stably expressed and therefore inconspicuous P450 cytochrome enzyme. The divergent chemical structure of rhodoxanthin requires a different biosynthesis pathway than the C4‐ketocarotenoids astaxanthin and canthaxanthin. Differential expression of *hsd3b*, encoding a dehydrogenase with a corresponding function in the steroid pathway, suggests a new candidate for rhodoxanthin biosynthesis.

## 1 Introduction

Body coloration plays important roles in various aspects of animal life, including courtship, social interactions, as well as camouflage and temperature regulation (Caro 2005; Maan and Sefc 2013). It is also involved in diversification and speciation processes in various taxonomic groups (Olsson et al. 2013; Maan and Sefc 2013; Rudh and Qvarnström 2013). Among the diverse animal pigment types known to date, red and yellow carotenoids have received particular attention as signals of individual quality and cues for mate choice (Svensson and Wong 2011; Maan et al. 2006; Simons et al. 2012; Sefc et al. 2014; Weaver et al. 2017; Weaver et al. 2018). This is because of the cost associated with the acquisition and metabolic conversion of dietary carotenoids—the only source of carotenoids for most animals—and their allocation away from vital physiological functions towards body colour displays (Powers and Hill 2021). Altogether, the signalling and physiological functions of carotenoids are reasonably well understood, at least in vertebrates (Blount and McGraw 2008; Svensson and Wong 2011; Powers and Hill 2021). In contrast, the investigation of the genetic, regulatory and biochemical basis of carotenoid coloration in animals has only more recently gained momentum (Toews et al. 2017 and references below). Knowledge of these molecular mechanisms contributes to the understanding of ecological and evolutionary processes behind carotenoid colour diversity, such as what facilitates the frequent evolutionary transitions between carotenoid‐based colours (Delhey et al. 2023; Friedman et al. 2014; Singh, Tschanz‐Lischer, et al. 2025).

The display of carotenoid pigments involves multiple physiological and metabolic steps, ranging from intestinal absorption, transport and metabolic conversion to integumentary deposition. Each of these steps can potentially be influenced by genetic and regulatory effects. The hue of carotenoid pigmentation depends on the number and position of conjugated double bonds along the molecule (Meléndez‐Martínez et al. 2007), and enzymatically catalysed conversions of dietary carotenoids allow animals to modify their carotenoid‐based colour displays. The higher the number of conjugated double bonds in the carotenoid molecule, the higher the wavelength of maximal absorption, such that yellow carotenoids are characterised by short, and red carotenoids by longer runs of conjugated double bonds (Meléndez‐Martínez et al. 2007). In the red ketocarotenoids, the conjugated double bond system of the polyene chain is extended by oxidation of the end rings (Twomey et al. 2020; Huang et al. 2021; Toomey et al. 2022). A key gene in the production of C4‐ketocarotenoids in birds is a member of the cytochrome P450 family, CYP2J19 (Mundy et al. 2016; Lopes et al. 2016; Twyman et al. 2016; Kirschel et al. 2020). However, as this gene occurs only in birds and turtles (Twyman et al. 2016), it cannot underlie carotenoid bioconversion in other taxonomic groups. Recent work has identified a different cytochrome P450 family 2 member, Cyp2ae2, as mediator of carotenoid ketolation in the zebrafish relative *Danio albolineatus* (Huang et al. 2021; Toomey et al. 2022). Two further cytochrome P450 family 2 members have been linked to the presence of C4‐ketocarotenoids in lizards (encoded by *cyp2j2* and *cyp2j6*; de Mello et al. 2021), and family 3 members were implicated in frogs (*cyp3A80*; Twomey et al. 2020), mites (*cyp384a1*; Wybouw et al. 2019) and shrimp (Weaver et al. 2020). In both birds and zebrafish, the cytochrome P450 enzyme is not sufficient to catalyse the full conversion of yellow to red carotenoids, which also requires the expression of a 3‐hydroxybutyrate dehydrogenase type 1 enzyme (BDH1L in birds, Bdh1a in fish; Toomey et al. 2022). Neither BDH1L nor Bdh1a produced ketocarotenoids in the absence of the corresponding cytochrome P450 enzyme. Assays interrogating the individual enzyme functions in birds revealed that BDH1L oxidises the product of CYP2J19 into red C4,4′‐ketocarotenoids, while BDH1L produces yellow canary xanthophyll in the absence of CYP2J19 (Toomey et al. 2022). Furthermore, the tetratricopeptide repeat protein 39b (TTC39B) enhanced ketocarotenoid synthesis in cultured cells co‐transfected with *CYP2J19* and *BDH1L* (Toomey et al. 2022), and genotypes of SNPs linked to avian *CYP2J19* and *TTC39B* are epistatically associated with bill coloration in long‐tailed finches (Hooper et al. 2024). Prior to these studies, expression levels of the *ttc39b* gene were found to be correlated with red coloration in fish (Ahi et al. 2020a; McKinnon et al. 2022; Salis et al. 2019) and poison frogs (Stuckert et al. 2021).

A molecular switch operated by the regulation of one (or both) of the two enzymes, cytochrome P450 or BDH1L/Bdh1a, would conceivably facilitate rapid evolutionary transitions between red and yellow body coloration. In fish, bioconversion of integumentary carotenoids is believed to take place in the skin (Hata and Hata 1976; Yamashita et al. 1996; Katsuyama and Matsuno 1988), and gene expression profiles in yellow and red fish skin may therefore be expected to differ between colour variants with respect to genes involved in carotenoid bioconversion. Alternatively, transitions between red and yellow body coloration may be caused by coding sequence changes in key carotenoid bioconversion genes, and this may also happen repeatedly in different evolutionary lineages. In the present study, we take advantage of phylogenetically replicated changes of carotenoid‐based body coloration that occurred during the diversification of East African cichlid fishes to investigate the gene regulatory mechanisms underlying yellow versus red body colour differentiation.

**FIGURE 1:**
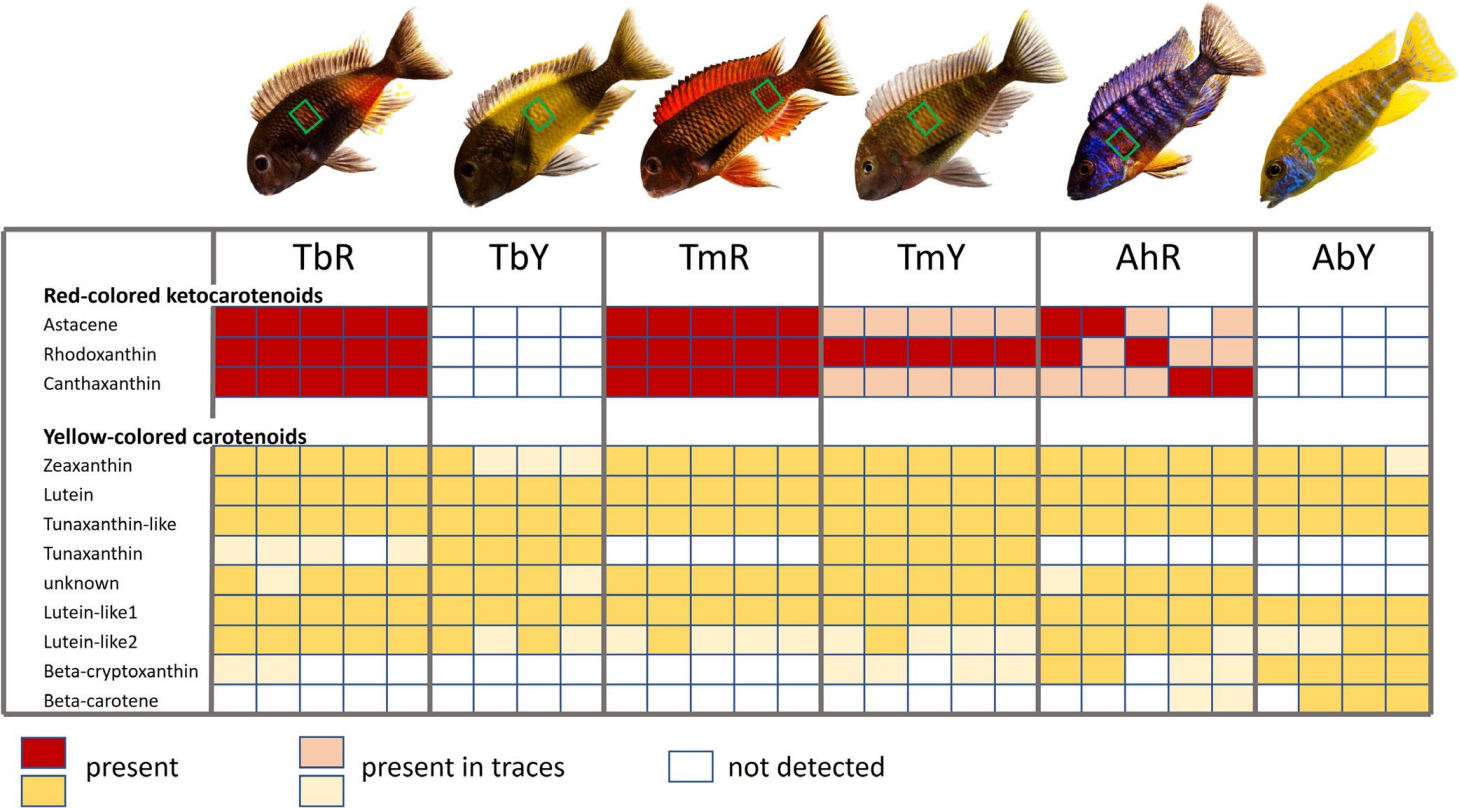
Carotenoids detected in the fish skin samples. Fish photographs above the table represent the investigated taxa. Tissue samples were taken from the body areas indicated by the squares. Cichlid taxa are: TbR, *T*. sp. ‘black’ ‘Bulu Point’ (red); TbY, *T*. sp. ‘black’ ‘Ikola’ (yellow); TmR, *T. moorii* ‘Moliro’ (red); TmY, *T. moorii* ‘Mbita’ (yellow); AhR, *A. hansbaenschi* ‘Red Flush’ (red); AbY, *A. baenschi* (yellow). In the table, each column represents a sample of the corresponding cichlid taxon; integumentary carotenoids are represented in rows. Cell colours indicate the detection of a particular carotenoid (red for ketocarotenoids, yellow for yellow carotenoids) in a particular sample. Photos by Wolfgang Gessl, used with permission.

The current study extends an earlier investigation (Ahi et al. 2020a), in which we compared skin transcriptomes from three pairs of related cichlid taxa that differed in carotenoid‐based yellow and red skin colour (Figure 1) and demonstrated consistently higher expression of *ttc39b* in the red tissue. Surprisingly, none of the other annotated transcripts displayed consistent expression differences between red and yellow taxa. The limitation of the previous study was that it used a *de novo* transcriptome assembly due to the absence of a closely related reference genome. Gene expression estimates from *de novo* assembled short‐read sequencing data can be noisy and biased (Freedman et al. 2021), motivating us to reanalyse the data using the recently published reference genome of *Tropheus moorii* (Fischer et al. 2021), which represents one of the taxa in our transcriptome study and is closely related to the other five taxa (all from the taxonomic tribe Haplochromini). We employed a robust bioinformatics pipeline for genome‐guided transcriptome assembly and expression quantification (Dobin et al. 2013; Pertea et al. 2015) developed to analyse phylogenetically nested transcriptomes (Singh, Ahi, et al. 2025). We expect this approach to provide better sensitivity and specificity into the gene expression dynamics of carotenoid‐based red and yellow colouration differentiation in East African cichlids. Additionally, the previous study (Ahi et al. 2020a) demonstrated variation in the carotenoid profiles among the investigated cichlid taxa, and we now conducted further chemical analyses to identify the carotenoid compounds responsible for the colour differences.

The power of the new gene expression analysis approach and the identification of integumentary carotenoids puts us in a strong position to progress in the search for a shared biochemical and molecular basis of red and yellow body coloration across cichlid taxa from different lineages. We first examine whether the yellow‐red colour differentiation is associated with the same types of carotenoids, and which gene expression levels differ consistently across the three taxon pairs. Then, given the presence of C4‐ketocarotenoids in the skin of the red fish, we asked whether the molecular mechanisms of ketocarotenoid‐based coloration in the red cichlids are similar to those characterised in zebrafish (Toomey et al. 2022). Specifically, do red and yellow cichlids differ in the expression levels of cichlid homologues of the C4‐ketocarotenoid biosynthesis genes *bdh1* and *cyp2ae2*?

## 2 Material and Methods

### 2.1 Samples Used in Gene Expression and Carotenoid Analysis

We used three pairs of closely related cichlid fish taxa to represent replicated differentiation in carotenoid‐based skin coloration. The taxon pairs (yellow vs. red) were (1) *Tropheus moorii* ‘Mbita’ versus *T. moorii* ‘Moliro’, (2) *T*. sp. ‘black’ ‘Ikola’ versus *T*. sp. ‘black’ ‘Bulu Point’, all four from Lake Tanganyika, and (3) *Aulonocara baenschi* versus *A. hansbaenschi* ‘Red Flush’ from Lake Malawi (Figure 1). The taxa are abbreviated as TmY, TmR, TbY, TbR, AbY, AhR (Figure 1) in the text and figures. The species‐level taxonomy in the genus *Tropheus* is largely unresolved. The genus includes numerous different colour variants (also referred to as colour morphs, although mostly allopatrically distributed; Egger et al. 2007) of unclear taxonomic status. The two *Tropheus* pairs used in this study belong to different genetic lineages separated by 1–2 million years of divergence (Egger et al. 2007). Divergence between the *Tropheus* taxa within each pair dates to approximately 100,000 years ago (Egger et al. 2007). The clades including the *Tropheus* (Lake Tanganyika Tropheini) and *Aulonocara* (Lake Malawi Haplochromini) are separated by approximately 5 million years of divergence (Irisarri et al. 2018), and divergence between the two *Aulonocara* species is in the range of 325,000–570,000 years (based on sequence data in Hashem et al. 2022 and substitution rates in Koblmüller et al. 2009 and Genner et al. 2007).

The present study re‐analyses RNA‐seq data of Ahi et al. (2020a) and tissue sampling for RNA extraction is briefly summarised here. Adult, captive‐bred fish were obtained from the aquarium trade. For a minimum of 4 weeks prior to the experiment, the fish were kept in the aquarium facility at University of Graz and fed identical flake food diets providing a mixture of algal, animal, and plant carotenoids (Spirulina Super Forte 36, Tropical). Before dissection, fish were sacrificed in a solution of 1–2 g Tricaine methanesulfonate (MS‐222) per 1 L water. Skin patches were sampled as indicated in Figure 1. Scales were removed and discarded. When tissue for RNA and carotenoid extraction was taken from the same fish, we sampled the same body region on opposite sides. Samples for RNA analysis were taken from six male fish per taxon and immediately transferred into RNAlater (Qiagen) and stored at −20°C.

Carotenoids were extracted from skin dissected from fish that had previously been sampled for RNA and then stored at −80°C (number of fish per colour variant, all males: TbR = 5; TbY = 2; TmY = 3; TmR = 3; AhR = 3; AbY = 3) and from additional fish that were sacrificed immediately before dissection (number of fish per colour variant: TbY = 2 females; TmY = 2 males; TmR = 2 females; AhR = 2 males; AbY = 1 male), yielding a sample size of 4–5 fish per colour variant.

### 2.2 Identification of Carotenoids

Skin tissue was cut into small (2 × 2 mm) pieces and extracted overnight at 4°C in a solution of acetone with butylated hydroxytoluene (BHT, 1 g/L) in volumes ranging from 150 to 1000 μL. The supernatant was recovered and stored at −20°C in the dark for up to 2 days prior to high‐performance liquid chromatography (HPLC) coupled to high‐resolution electrospray ionisation mass spectrometry (MS) analysis. Samples were centrifuged at 20.817*g* for 4 min prior to transfer into HPLC vials. As hydroxylated carotenoids occur as fatty ester acids in the fish skin, we analysed both free carotenoids (crude extracts before saponification) and saponified extracts. For saponification, 40 μL of each extract was incubated with 0.25% methanolic sodium hydroxide (NaOH) for 24 h at 4°C in the dark.

Carotenoids in both crude and saponified extracts were separated by HPLC, and retention times and absorption spectra were compared to signals obtained from carotenoid standards (Figures S1 and S2, Data S1). Carotenoid standards were purchased from CaroteNature GmbH, Münsingen, Switzerland (product numbers in subsequent brackets): (3RS,3′RS)‐astaxanthin (No. 0403), astacene (No. 0398), zeaxanthin (No. 0119), lutein (No. 0133), tunaxanthin (No. 0149), rhodoxanthin (No. 0424), canthaxanthin (No. 0380), beta‐cryptoxanthin (No. 0055), (rac.)‐alpha‐carotene (No. 0007.1) and beta‐carotene (No. 0003). This analysis was carried out in the Agilent 1290 UHPLC System (Agilent Technologies, Waldbronn, Germany) with an Agilent Zorbax Eclipse Plus C18 column (2.1 × 150 mm, 1.8 μm Rapid resolution HD, Agilent Technologies Waldbronn, Germany). Mobile phase A consisted of acetonitrile (gradient grade, VWR International, UK), water (18.2 MΩ cm, obtained from a Millipore Milli‐Q reference ultrapure water purification system, USA) and formic acid (MS grade, Merck, Germany) in a ratio of 80 + 20 + 0.1 v/v; mobile phase B consisted of 2‐propanol (HPLC grade, VWR International, UK). A gradient with the following settings was used at a flow rate of 0.49 mL/min: 0 to 13.5 min 0% B to 100% B, 13.5 to 15 min 100% B, and 3 min equilibration with 0% B. An injection volume of 1 μL was used, and the column temperature was set to 55°C. The detector obtained UV/VIS spectra (210–640 nm, 2 nm steps), and wavelengths of 440 and 480 nm with a bandwidth of 8 nm and a sampling rate of 40 Hz were recorded.

For MS analyses, saponified extracts were separated on a Dionex Ultimate 3000 UHPLC System (Thermo Scientific, Waltham, USA) connected to a Q‐Exactive Orbitrap mass spectrometer equipped with an electrospray HESI‐II ionisation source (Thermo Scientific, Waltham, USA), using the same HPLC column and mobile phases as above. A gradient with the following settings was used at a flow rate of 0.5 mL/min: 0 to 13.5 min 0% B to 90% B, 13.5 to 20 min 90% B, from 20 to 20.5 min equilibration from 90% B to 0% B, which was then kept for 4.5 min. Injection volumes were 5 μL for carotenoid extracts and 1 μL for carotenoid standards. The detector obtained UV/VIS spectra (220–800 nm, 2 nm steps), and the wavelengths of 440 and 480 nm with a bandwidth of 2 nm and a sampling rate of 100 Hz were recorded.

MS/MS experiments were performed in positive ionisation mode with spray voltages of +3.5 kV. Nitrogen was used as drying gas at a temperature of 350°C; sheath gas flow was set to 50 instrument units (IU) and auxiliary gas flow was 12.5 IU; capillary temperature was set to 265°C. The full MS scan range was set to 250–900 m/z at a resolution of 70000 (full width at half maximum (FWHM), specified at m/z 200) with product reaction monitoring (PRM) MS/MS of the precursor masses of the carotenoid standards (Table S1). The fragmentation with combined normalised collision energies of 20, 40 and 60 was performed at a resolution of 35,000 (FWHM). Accurate masses were considered consistent with specific carotenoids when the mass difference between measured and theoretical m/z was below 1 ppm.

Specific carotenoids were scored as ‘present’ in the skin samples, when their UV absorbance signal exceeded 3‐fold the signal‐to‐noise significance level compared to baseline, and their retention time, assignment of accurate mass, isotope pattern, m/z ratio and fraction pattern in MS/MS experiment matched those of a carotenoid standard. Additionally, we scored carotenoids as ‘present in traces’, when the corresponding mass was detected and the isotope pattern, m/z ratios and fraction pattern in MS/MS experiments matched those of a carotenoid standard, but the UV absorbance signal did not reach the significance level. UV absorbance signals that could not be assigned to any of the standards were tested for carotenoid‐specific isotope patterns and labelled as ‘tunaxanthin‐like’ (based on accurate mass, absorption spectra and m/z ratios), ‘lutein‐like’ (based on accurate mass) and ‘unknown’. These features were compared between samples for their characteristic retention times, mass and m/z ratios and scored as ‘present’ and ‘present in traces’ likewise.

### 2.3 RNA Data Reanalysis Using Genome Guided Transcriptome Assembly

We reanalyzed the RNA‐seq data from Ahi et al. (2020a; NCBI SRA PRJNA658843) using an improved bioinformatics pipeline (Singh, Ahi, et al. 2025) and the recently published reference genome of *Tropheus moorii* (version 1.0; Fischer et al. 2021). After a quality check with Fastqc (v0.11.8) (Andrews 2012) and a trimming step with Trimmomatic (v0.3.9) (Bolger et al. 2014), only reads with a phred > 28 and a minimum length of 70 bp were retained, resulting in a median of 11.5 million reads per sample. For read alignment we used RNAstar (v2.7.3.a) (Dobin et al. 2013) with the *T. moorii* reference genome as guide reference. A median of 83% of input reads per sample mapped to the reference genome. After checking the mapping statistics with samtools idxstats (v1.9) (Danecek et al. 2021) we used StringTie (v2.0.6) (Pertea et al. 2015) to assemble the RNA‐seq alignments into transcripts The transcript/gene annotation for each biological replicate was then merged for each species. To estimate the accuracy of the produced annotation files we compared them with gffcompare (v0.11.2) (Pertea and Pertea 2020) to the *T. moorii* reference annotation. We removed monoexonic transcripts not contained in the reference and a class code assigned by gffcompare, indicating ‘possible polymerase run‐on’ fragments. The maximum intron length was reduced to 200,000 bp, which is the maximum intron length found in the *T. moorii* reference. Using the final improved gene annotation, we estimated gene expression with StringTie allowing no multimapping. From these expression estimates, count matrices were produced using a Perl script from the Griffith lab (https://github.com/griffithlab/rnaseq_tutorial/blob/master/scripts/stringtie_expression_matrix.pl) to extract raw count data from StringTie results. Scripts for the RNA‐seq pipeline we used can be found on github: https://github.com/poojasingh09/2024_singh_transcriptomeanalysis_pipeline

### 2.4 Gene Expression Analysis and Analysis of DNA Sequence Variation

We used DESeq2 (Love et al. 2014) in R (R Core Team 2020) using RStudio (RStudio Team 2021) to detect differentially expressed genes running comparisons between all red‐yellow species‐pairs. DESeq2 uses raw read counts and estimates variance–mean dependence based on a model that utilises the negative binomial distribution (Love et al. 2014). *p*‐values were corrected for multiple comparisons using the method of Benjamini and Hochberg (1995). The cutoff for differentially expressed genes was chosen at a false discovery rate of *p*.adj. < 0.05.

Pearson correlation coefficients between expression levels of focal genes (VST normalised read count data) were calculated and visualised in R 4.4.1 using the functions ‘cor’, ‘cor_pmat’ and ‘ggcorrplot’ in the package ‘ggcorrplot’ (Kassambara 2023).

To assess patterns of allelic variation in *bdh1l* and *ttc39b* between yellow and red taxon pairs, reads were extracted based on their mapping coordinates (candidate gene body ± 1000 bp) using samtools (v1.9; Danecek et al. 2021) and bedtools (bamtofastq; v.2.29; Quinlan and Hall 2010). Extracted reads were then mapped to the corresponding coding sequence extracted from the genomes of *T. moorii* (Fischer et al. 2021; all *Tropheus* samples) and *Metriaclima zebra* (Ensembl release 113; all *Aulonocara* samples) genomes, respectively, using bowtie2 (v2.3.5; Langmead and Salzberg 2012). Single nucleotide polymorphisms (SNPs) were called using samtools (mpileup, v1.9) and converted to vcf format using bcftools (v.1.9). Consensus sequences per sample were generated using vcfutils (vcf2fq) excluding positions with either read coverage or root mean square (RMS) mapping quality below 10. Nucleotide and amino acid sequence alignments across all samples were inspected in AliView (Larsson 2014).

## 3 Results

### 3.1 Comparison Between the Previous and Current Analysis

The transcriptome assembly used in the previous analysis by Ahi et al. (2020a) was less complete (BUSCO completeness score 82.8%) than the *T. moorii* genome (Fischer et al. 2021) used in the current analysis (BUSCO completeness score 96.4%, Table S2). The unique mapping rate of RNA reads in the previous analysis was much lower than that of the current analysis (mean across samples 16.5% vs. 82.4%), and the mapping rate was similar across samples in the current analysis (*p*‐values > 0.2 in all pairwise taxon comparisons; Table S2). The previous analysis had ~134,000 genes in the transcriptome assembly versus the 34,305 genes in the current genome guided analysis, the latter being consistent with the expectation of cichlid genomes and transcriptomes (Brawand et al. 2014). The high number of genes in the transcriptome assembly approach (Ahi et al. 2020a) was most likely an artefact of isoforms from the same gene being treated as independent genes. This resulted in a high percentage of ambiguously aligned reads (mean across samples 83.4%; Table S2) in the previous analysis, which most likely impacted the gene expression estimates and thus the differential gene expression results.

### 3.2 Red Body Coloration Is due to Rhodoxanthin and C4‐Ketocarotenoids

We found that ketocarotenoids were present in the skin samples of each of the three red taxa, but only in one of the yellow taxa, *T. moorii* ‘Mbita’ (Figure 1, Table S3). The ketocarotenoids were identified as rhodoxanthin, a C3,3′‐ketocarotenoid, and the C4,4′‐ketocarotenoids canthaxanthin and astacene. Astacene was detected only after saponification and is most likely a product of the alkaline saponification process via degradation of esterified astaxanthin, also a C4,4′‐ketocarotenoid (Su et al. 2018; Negro and Garrido‐Fernandez 2000). The yellow carotenoids zeaxanthin, lutein, and un‐identified tunaxanthin‐ and lutein‐like compounds occurred in all taxa (Figure 1). Tunaxanthin was present in the yellow *Tropheus* taxa and in traces in the red *T*. sp. ‘black’ ‘Bulu Point’ (TbR), but was absent from both *Aulonocara* species. In contrast, apart from traces of beta‐cryptoxanthin in a few *Tropheus* samples, beta‐cryptoxanthin and beta‐carotene were specific to *Aulonocara*.

Hierarchical clustering of samples based on their carotenoid profiles (carotenoid compounds scored as 2, 1 and 0 for ‘present’, ‘present in traces’ and ‘absent’, respectively) revealed distinct, taxon‐specific clusters of yellow samples, and a fourth cluster containing the red samples (Figure S3).

### 3.3 Expression Differences of Carotenoid Coloration‐Associated Genes Between Red and Yellow Cichlids

**FIGURE 2:**
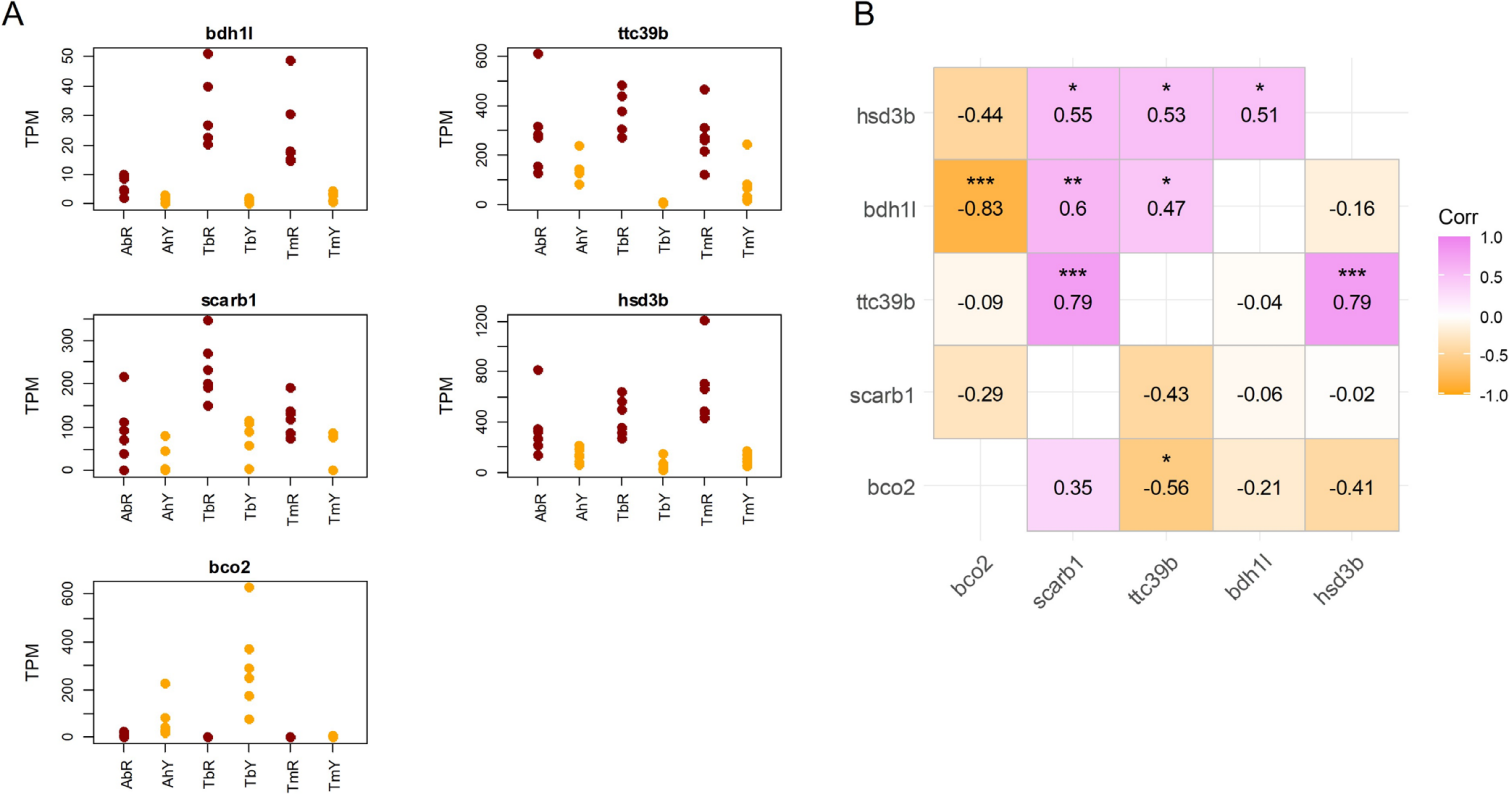
Expression patterns of carotenoid colour genes *bdh1l*, *ttc39b*, *scarb1*, *hsd3b* and *bco2*. (A) Transcript abundance (TPM, transcripts per million). See Table S5 for TPM values. Some dots overlap, *n* = 6 for each taxon. Cichlid taxa are: TbR, *T*. sp. ‘black’ ‘Bulu Point’ (red); TbY, *T*. sp. ‘black’ ‘Ikola’ (yellow); TmR, *T. moorii* ‘Moliro’ (red); TmY, *T. moorii* ‘Mbita’ (yellow); AhR, *A. hansbaenschi* ‘Red Flush’ (red); AbY, *A. baenschi* (yellow). (B) Pearson correlation coefficients calculated from VST normalised count data. Above diagonal: gene expression correlations in the red samples (AhR, TbR and TmR combined); below diagonal: gene expression correlations in the yellow samples (AbY, TbY and TmY combined). Asterisks indicate uncorrected significance levels: ***, *p* < 0.001; **, 0.001 < *p* < 0.01; *, 0.01 < *p* < 0.05.

The number of differentially expressed (DE) transcripts varied among taxon pairs, with most DE transcripts detected in the comparison between *Tropheus moorii* ‘Mbita’ and *T. moorii* ‘Moliro’ (TmR vs. TmY, *n* = 4163 transcripts, 3855 annotated transcripts representing 3419 different gene names), followed by the comparison between *T*. sp. ‘black’ ‘Ikola’ and *T*. sp. ‘black’ ‘Bulu Point’ (TbR vs. TbY, *n* = 1146 transcripts, 1015 annotated transcripts representing 922 different gene names) and *Aulonocara baenschi* versus *A. hansbaenschi* ‘Red Flush’ (AhR vs. AbY, *n* = 618 transcripts, 539 annotated transcripts representing 488 different gene names) (Table S4, Figures S4 and S6). The complete DESeq2 results for each taxon pair are provided in Table S4. Only 25 annotated transcripts were differentially expressed in all of the three taxon pairs (Table S4) after correction for multiple testing, and expression levels of only nine of these 25 transcripts differed consistently in the same direction (either higher or lower in the red taxon) in all taxon pairs. Genes with statistically significantly higher expression in red than in yellow taxa across all three pairs included the carotenoid colour genes D‐beta‐hydroxybutyrate dehydrogenase mitochondrial‐like (*bdh1l*; Figure 2a) and an isoform of scavenger receptor class B member 1 (*scarb1*; Figure 2a), in addition to a homeobox protein HMX2‐like transcript (associated with inner ear development, Wang and Lufkin 2005) and isoforms of cadherin‐13 (*cdh13*). Elevated expression of *ttc39b* in red taxa, as reported in Ahi et al. (2020a), was also detected in the current analysis (Figure 2a), but was not significant in one of the pairs (AhR vs. AbY) after correction for multiple comparisons (Table S4). In fact, expression differences of carotenoid genes were generally weaker between the *Aulonocara* compared to the *Tropheus* taxa, which might be connected with the overall lower concentration of carotenoids in the skin of the red *Aulonocara* (Figures 1 and 2a). The five transcripts with consistent higher expression in yellow than in red taxa were beta‐carotene 9′10′‐oxygenase‐like (*bco2*, Figure 2a), which mediates cleavage of carotenoids, along with melanocortin receptor 5 (*mc5r*), interleukin‐10 receptor subunit beta‐like (*il10rb*), an unidentified transcript (TM1_G0000024895 in the *T. moorii* genome annotation, version 1.0; Fischer et al. 2021) and transmembrane protein 256 (*tmem256*) isoform. Reads of *tmem256* were detected in only four samples (2 AbY, 1 TbY and 1 TmY), where they ranged between 4 and 12 TPM.

We also screened the DE gene sets of the individual transcriptome comparisons for taxon pair‐specific signals of additional candidate carotenoid genes (beta‐carotene‐15,15′‐oxygenase *bco1*, fatty acid 2‐hydroxylase *fa2h*, as well as retinol dehydrogenases, hydroxysteroid dehydrogenases, dehydrogenase/reductase SDR gene family members; Koch et al. 2025). There were no significant differential expression signals for *fa2h* and *bco1* in any of the transcriptome comparisons, while three retinol dehydrogenase genes (*rdh10*, *rdh3‐like* and *rdh12*) showed elevated expression in the red TmR compared to the yellow TmY (Table S4). Interestingly, the transcripts of two short‐chain dehydrogenase/reductase (SDR) superfamily gene members, 3‐beta‐hydroxysteroid dehydrogenase (*hsd3b*) and dehydrogenase/reductase SDR family member 11 (*dhrs11*), were significantly more abundant in red than in yellow fish across all taxon pairs, when cases with uncorrected *p*‐values < 0.05 were considered (Figure 2a; all results in Table S4). The *T. moorii* reference genome includes multiple copies and isoforms of *dhrs11*, and the RNA‐seq reads mapped to several of them, complicating accurate expression level comparisons across taxa. Additionally, the abundance of *dhrs12‐like* transcripts was elevated in the red TmR and TbY, but not in AhR, and the abundance of *dhrsx‐like* transcripts was elevated in the red AhR and TbR, but reduced in TbR (Table S4).

Expression levels of the differentially expressed carotenoid colour genes *ttc39b*, *scarb1, hsd3b* and *bdh1l* were significantly positively correlated with each other in the red fish (Figure 2b). In the yellow fish, the only positive expression correlation was between *ttc39b* and *hsd3b* (Figure 2b). In addition, we tested for expression correlations between the differentially expressed transcription factor *hmx2‐like* and the differentially expressed carotenoid colour genes. In the yellow fish, expression levels of *hmx2‐like* correlated positively with *ttc39b* expression (*r* = 0.71, *p* = 0.001), and in both red and yellow fish, expression of *hmx2‐like* was correlated negatively with *scarb1* expression (red fish: *r* = −0.52, *p* = 0.027; yellow fish: *r* = −0.58, *p* = 0.011).

### 3.4 Coding Sequence Comparisons

We investigated allelic variation of the coding sequences of *bdh1l* and *ttc39b*, that is, the two genes which are known to function in ketocarotenoid biosynthesis and whose expression levels differed between red and yellow fish in our study. The amino acid sequences coded by *bdh1l* were highly conserved among the investigated fish (mean similarity 99.7%, minimum similarity 97.44%, across comparisons between fish) as well as in comparison to reference genomes of *T. moorii* (Fischer et al. 2021) and *M. zebra* (Conte and Kocher 2015) (Figure S7). The amino acid sequence alignment of *ttc39b* contained several polymorphic sites, including fixed differences between *Tropheus* and *Aulonocara* and several taxon‐specific substitutions (Figure S8). There were no fixed protein‐coding polymorphisms associated with either red or yellow body coloration across taxa.

### 3.5 No Consistent Differential Expression of *cyp2* Genes Between Cichlid Colour Variants

Neither *cyp2ae2* nor its ortholog *cyp2ae1* (Huang et al. 2021) are included in the *T. moorii* genome annotation. DNA sequence information of *Danio cyp2ae2* is not available, but the protein sequence of *cyp2ae2* (Toomey et al. 2022) is most similar to cichlid *cyp2j6* and *cyp2j2* (~45% identity, as determined by a blastp search of the *cyp2ae2* amino acid query sequence against “Cichlidae” coding sequence data in Genbank). The differentially expressed genes for the red/yellow taxon pairs included several P450 genes (Table S4), but none displayed consistent differential expression patterns across all taxon pairs. Expression levels of a *cyp2j2*‐like transcript were significantly higher in the red fish in one taxon pair (TmR vs. TmY), but significantly lower in another (TbR vs. TbY), and not significantly different from those of yellow fish in the third pair (AhR vs. AbY). Each of the skin samples expressed one or more isoforms of *cyp2j2*‐like transcripts (Table S4). No colour‐dependent differential expression was detected for transcripts of *cyp2j6* in any of the taxon pairs.

## 4 Discussion

Carotenoid based body coloration is an important component of animal signalling and species diversification in many lineages, though its molecular and genetic underpinnings remain less understood. Our study showed that the red‐versus‐yellow body colour contrast between sister taxa of cichlid fishes is due to the presence or absence of C3‐ and C4‐ketocarotenoids in their skin. Consistent differential expression—higher in red compared to yellow fish—of the genes *bdh1l* and *ttc39b*, whose products are known to mediate C4‐ketocarotenoid biosynthesis (Huang et al. 2021; Toomey et al. 2022), across the three taxon pairs furthermore points to parallel molecular mechanisms contributing to the body colour differences in these taxa. In particular, the expression patterns discovered in this study suggest that *bdh1l* expression might be pivotal for C4‐ketocarotenoid biosynthesis in the red colour variants. *Bdh1* encodes a short‐chain dehydrogenase/reductase, which interconverts hydroxyl and ketone groups (Otsuka et al. 2020). We detected significant differential expression of *bdh1l* in the analysis of standardised read counts in each of the taxon pairs. Moreover, transcript abundance of *bdh1l* (Figure 2a, Table S5) was low in the yellow AbY (mean TPM, 0.87) and TbY (mean TPM, 1.12), and slightly higher in TmY (2.33), which also expresses a low amount of C4‐ketocarotenoids according to our HPLC/MS analyses. Rapid and recurrent evolutionary transitions between colour trait states would be facilitated if mediated by simple molecular changes. Our results on expression differences and the absence of protein‐coding changes in *bdh1*, together with the BDH1 enzyme functions reported by Huang et al. (2021) and Toomey et al. (2022), are consistent with the hypothesis that *bdh1l* gene expression is an important factor in cichlid body colour diversification by regulating the production of red C4‐ketocarotenoids.

Huang et al. (2021) demonstrated that knock‐out mutant *Danio albolineatus* deficient in either *bdh1a* or in the cytochrome P450 family member *cyp2ae2* did not produce ketocarotenoids. The requirement of both genes for ketocarotenoid production from yellow carotenoids was then confirmed in cultured cells and transgenic *D. rerio* (Toomey et al. 2022). A cichlid homologue of *cyp2ae2* has not yet been identified. In our study, no P450 family member showed consistent colour‐related expression differences across the taxon pairs. The genome annotation of *T. moorii* contains multiple putative paralogs (Fischer et al. 2021) for the two P450 genes that are most similar to *cyp2ae2* in cichlids (*cyp2j2* and *cyp2j6*), and it is possible that the presence of these paralogues hampers accurate gene expression estimation. However, pooling reads that were mapped to the paralogues of either *cyp2j2* or *cyp2j6* did not indicate colour‐related transcript abundance variation shared among the three taxon pairs, either. Possibly, different P450 enzymes contribute to ketocarotenoid production in each of the red taxa, for instance the products of *cyp2j2‐like* in TmR and *cyp2a8‐like* in AhR (Table S4). Alternatively, as our study did not come up with a single compelling P450 candidate for a role in ketocarotenoid biosynthesis in cichlid fish, it is also plausible that the dramatically reduced expression of *bdh1l* in the yellow cichlids is alone sufficient to suppress the production of C4‐ketocarotenoids, even if the expression of the corresponding P450 enzyme is similar between red and yellow fish. This is consistent with the observation that BDH1L generates C4‐ketocarotenoids from the intermediate product of CYP2J19 in birds (Toomey et al. 2022; Koch et al. 2025).

Another molecular player involved in C4‐ketocarotenoid biosynthesis is the tetratricopeptide repeat protein TTC39B (Toomey et al. 2022; Hooper et al. 2024). In cells co‐transfected with avian CYP2J19 and BDH1L, the conversion of yellow carotenoids to C4‐ketocarotenoids was enhanced by the additional expression of *TTC39B* (Toomey et al. 2022; Koch et al. 2025). How TTC39B modulates ketocarotenoid biosynthesis is still unclear. Toomey et al. (2022) hypothesised a role in carotenoid transport to or from the sites of enzymatic conversion. Indeed, various fish species displayed elevated *ttc39b* expression in yellow and red carotenoid‐coloured skin tissue compared to skin tissue devoid of carotenoids (Salis et al. 2019; Ahi et al. 2020b; Liang et al. 2020; Kawamoto et al. 2021; McKinnon et al. 2022). In our previous study comparing red and yellow skin tissue (Ahi et al. 2020a), we showed that both *ttc39b* expression and total carotenoid concentration were higher in the red than in the yellow skin samples. The revised analysis of the RNASeq data in the present study confirmed the covariation of *ttc39b* expression with red body coloration. It also uncovered consistent differential expression of *scarb1* in all taxon pairs, which had not been detected in the previous analysis. Scavenger receptor class B membrane proteins mediate the transport and cellular uptake of carotenoids and have been associated with carotenoid coloration across a range of animals, including insects (Tsuchida and Sakudoh 2015), fish (Sundvold et al. 2011; Ahi et al. 2020; Saunders et al. 2019), frogs (Rodríguez et al. 2020), lizards (McLean et al. 2017; de Mello et al. 2021) and birds (Toomey et al. 2017). As hypothesised for *ttc39b*, the consistently elevated expression of *scarb1* in red compared to yellow skin samples may be associated with processes controlling the accumulation of carotenoids in the integument.

The carotenoid extracts of the cichlid skin tissues were rich in esterified carotenoids, and only rhodoxanthin and canthaxanthin, both of which lack hydroxyl groups and can therefore not form esters, appeared as free carotenoids before saponification (Figure S2). The saponification step hydrolysed the esters completely and released the free carotenoids for identification. As expected after alkaline saponification, we did not detect astaxanthin, which is a common carotenoid in the tissue of aquatic animals, but its saponification product astacene (Negro and Garrido‐Fernandez 2000; Su et al. 2018), and saponification protocols need to be adjusted to detect both weakly and strongly oxidised carotenoids in the mixed extracts (Toomey and McGraw 2007). All of the yellow carotenoids detected in the yellow skin samples were also present in at least some of the red skin samples, where they might serve as substrate for ketocarotenoid biosynthesis. The here identified yellow carotenoids as well as canthaxanthin, astaxanthin, and rhodoxanthin have been detected in the skin of cichlid fish before (Sefc et al. 2014).

Of note, the C3‐retro‐carotenoid rhodoxanthin differs from the other two C4‐ketocarotenoids in the position of the ketogroups on the end rings and in the biochemical pathways by which it is formed. C4‐ketocarotenoids are formed by the addition of carbonyl groups to the C4‐positions on the end rings of, for example, zeaxanthin, and the pathway involves BDH1L and a cytochrome P450 enzyme in various taxa (but see Koch et al. 2025). In contrast, as reconstructed in birds, the formation of rhodoxanthin from its precursors zeaxanthin or lutein presumably involves the dehydrogenation of hydroxyl groups at the C3 positions of beta and epsilon rings, hydroxylation, dehydration and rearrangements of double bonds (Prum et al. 2012; Hudon et al. 2012). In plants, the biosynthesis of rhodoxanthin similarly involves 3‐beta hydroxyl dehydrogenation and rearrangement of double bonds (Royer et al. 2020). Rhodoxanthin is a predominant carotenoid in the skin of the cichlid fish tilapia, but was not detected in any other tissue (Katsuyama and Matsuno 1988). While it has been suggested that birds and fish are able to endogenously synthesise rhodoxanthin from zeaxanthin and lutein (Hudon et al. 2007; Katsuyama and Matsuno 1988; Prum et al. 2012), no genes have yet been linked to the pathway in animals. Intriguingly, products of two carotenoid candidate genes with higher expression in the red than in the yellow taxa in each of the three taxon pairs in the current study, *hsd3b* and *dhrs11*, possess 3‐beta hydroxysteroid dehydrogenase activity (Simard et al. 2005; Endo et al. 2016). There are multiple copies of *dhrs11* in the genome of the Nile tilapia (Zhang et al. 2021), and several copies and isoforms of *dhrs11* are also included in the annotation of the *T. moorii* genome. This may dilute the gene expression signal across paralogs and isoforms of paralogs (Table S4), which also prevented its detection in the initial analysis. Human DHRS11 expresses reductive 3‐beta hydroxysteroid dehydrogenase activity and converts C3 keto‐groups to hydroxyl groups (Endo et al. 2016; Endo et al. 2019), a function which might also contribute to the carotenoid metabolism. The signal of *hsd3b* in the *Aulonocara* pair was statistically significant only before correction for multiple comparisons (Table S4), which is why it was not included in the list of consistently differentially expressed genes, which was based on corrected *p*‐values. Mammalian HSD3B catalyses the oxidative transformation of hydroxyl‐ to keto‐groups on the 3C position of beta‐rings and a shift of double bonds as a step in the formation of steroids (Simard et al. 2005), that is, biochemical conversions that are also part of the rhodoxanthin pathway. To our knowledge, HSD3B has not yet been linked to rhodoxanthin biosynthesis, but its capability to oxidise 3‐beta hydroxy groups and shift double bonds makes it an interesting candidate.

The interpretation of the observed differential expression patterns relies on the assumption that the crucial biochemical steps differentiating yellow from red fish take place in the skin, that is, the tissue examined in the transcriptome comparisons. In the cichlid fish *Pseudocrenilabrus multicolor* , the mix of carotenoids detected in the skin contained different carotenoids than that detected in the plasma (Williams et al. 2025), suggesting the in situ production of skin‐specific carotenoid species. Reductive pathways, for example, bioconversion of astaxanthin or canthaxanthin to yellow carotenoids, have been shown to occur both in the liver and the integument of fish (tilapia, Katsuyama and Matsuno 1988; rainbow trout, Guillou et al. 1989; Katsuyama et al. 1987; black bass, Yamashita et al. 1996; Synowiecki et al. 1996; yellowtail, Miki et al. 1985). Oxidative conversions have been located to the skin in tilapia, which is the same family as our study fish (Katsuyama and Matsuno 1988), and in red carp (Hata and Hata 1976), while rainbow trout biosynthesised astaxanthin from either zeaxanthin or canthaxanthin in the liver (Guillou et al. 1989). The red and yellow fish taxa examined here develop their different colours on the basis of the same diet under natural conditions as well as on the basis of the same flake food in laboratory conditions. Both the natural diet and the flake food include ketocarotenoids, for example, from crustacean and cyanobacterial ingredients, which are not deposited in the skin of the yellow fish. Our comparative transcriptome data did not identify compelling candidate genes for the conversion of red into yellow carotenoids. One of the reasons for this could be higher genetic diversity among taxa or genotypic redundancy within taxa in the biological pathways that control red to yellow colour conversion versus those that control yellow to red colour conservation (Láruson et al. 2020; Singh, Tschanz‐Lischer, et al. 2025). Alternatively, the absence of ketocarotenoids in the skin of yellow fish and the expression of a ketolase in the skin of red fish suggests that dietary ketocarotenoids might first be metabolised in a reductive pathway, which may even be shared by all studied taxa and may take place in organs other than the skin, followed by ketocarotenoid biosynthesis in the skin of red fish (Katsuyama and Matsuno 1988).

One of the five candidates with elevated expression in the yellow skin samples was a gene that codes for a carotenoid‐cleaving enzyme, the beta‐carotene oxygenase Bco2. Reduced and increased expression of *bco2* often go along with the accumulation and absence, respectively, of carotenoids in the respective tissue (Lehnert et al. 2019; Gazda et al. 2020; Våge and Boman 2010; Ahi et al. 2020b). The elevated abundance of *bco2* transcripts measured in the yellow skin samples of TbY stands in contrast to the high concentration of carotenoids that was measured previously in the yellow skin of this fish (Ziegelbecker et al. 2021). The other known colour gene with higher transcript abundance in yellow skin samples codes for the melanocortin 5 receptor, which is part of the melanocortin system that regulates (among other processes) the pigmentation of fish (Cal et al. 2017). Mc5r is associated with xanthophores in flounders and goldfish, and may be associated with pigment dispersion (Kobayashi et al. 2012; Kobayashi et al. 2010; Mizusawa et al. 2018). In contrast to our results, *mc5r* transcripts were more abundant in the erythrophore regions than in the xanthophore regions of the fins of *Danio albolineatus* (Huang et al. 2021).

Individual transcriptome comparisons typically bring forth hundreds to thousands of differentially expressed transcripts. To narrow down the pool of candidates, we combined classical differential expression analyses with a rigorous comparative approach and focused on genes which are consistently differentially expressed in three independent, closely related cichlid fish taxon pairs displaying the same phenotypic contrast (yellow vs. red coloration). Our study also makes a case for reanalysing RNA‐seq data when better (e.g., taxonomically closer or qualitatively improved) reference genome assemblies become available and bioinformatics tools with higher precision are developed, as this can reveal new insights that were previously overlooked for technical reasons (Rhie et al. 2021). Our analysis brought forth several consistently differentially expressed genes, including well‐known carotenoid colour genes (*scarb1*, *bco2*), as well as two genes (*bdh1l*, *ttc39b*) that have only recently been shown to function in C4‐ketocarotenoid biosynthesis (Huang et al. 2021; Toomey et al. 2022) and a potential novel candidate for C3‐ketocarotenoid production (*hsd3b*). In particular, the correlation between *bdh1l* expression and the presence of C4‐ketocarotenoids suggests a simple molecular mechanism that can contribute to carotenoid‐based body colour diversification in cichlid fishes. Furthermore, a small number of genes hitherto not directly linked to carotenoid metabolism (*hmx2‐like*, *il10rb*, *mc5r*) also showed consistent colour‐correlated expression levels and might constitute promising targets for further studies.

## Author Contributions

Kristina M. Sefc and Ehsan Pashay Ahi designed the study. Ehsan Pashay Ahi performed the RNA sequencing. Pooja Singh developed the RNA‐seq pipeline and conducted the RNA‐seq data analysis with contributions from Kristina M. Sefc and Christoph Hahn. Angelika Ziegelbecker, Ronald A. Glabonjat and Walter Goessler performed the identification of carotenoids. Kristina M. Sefc wrote and revised the manuscript with significant contributions from all authors.

## Disclosure

Benefit Sharing Statement: Benefits Generated: Benefits from this research accrue from the sharing of our data and results on public databases as described above.

## Conflicts of Interest

The authors declare no conflicts of interest.

## Supporting information


**Data S1:** mec70065‐sup‐0001‐Supinfo.docx.

## Data Availability

Sequence reads from the RNA‐Seq experiment are available from the NCBI sequence read archive under the accession number PRJNA658843 (https://www.ncbi.nlm.nih.gov/bioproject/658843). Smaller datasets are provided as Supporting Information. The code used for the analysis is available on Dryad (https://doi.org/10.5061/dryad.80gb5mm0n).
