# Supplementary material for: Bdh1l Gene Expression Is a Potential Molecular Factor in the Evolution of Carotenoid‐Based Colour Diversity of Cichlid Fishes
Source: Mol Ecol. 2025 Aug 20;34(18):e70065. doi: 10.1111/mec.70065 (PMC12421482; doi:10.1111/mec.70065)
Supplement: Supplementary file 1 — Data S1: mec70065‐sup‐0001‐Supinfo.docx. [file MEC-34-e70065-s001.zip › mec70065-sup-0001-Supinfo.docx]

**Supplemental Information for:**

**Bdh1l gene expression is a potential molecular switch in the evolution of carotenoid-based color diversity of cichlid fish**

Pooja Singh, Angelika Ziegelbecker, Christoph Hahn, Walter Goessler, Ronald A. Glabonjat, Ehsan Pashay Ahi, Kristina M. Sefc

**Table of Contents:**

| **Table S1** | Page 2 |
| --- | --- |
| **Table S5** | Page 3 |
| **Figure S1** | Page 5 |
| **Figure S2** | Page 6 |
| **Figure S3** | Page 7 |
| **Figure S4** | Page 8 |
| **Figure S5** | Page 9 |
| **Figure S6** | Page 10 |
| **Figure S7** | Page 11 |
| **Figure S8** | Page 31 |

Note that Table S2, Table S3 and Table S4 are provided in separate files.

**Table S1:** m/z of the respective carotenoid candidates (gained from carotenoid standards) tracked in SIM-mode. In bold: precursor mass of greater abundance. For HPLC chromatogram of carotenoid standards, see Fig. S7.

| carotenoid | M^+^ | [M+H]^+^ |
| --- | --- | --- |
| astaxanthin | 596.3860 | **597.3939** |
| astacene | 592.3547 | **593.3625** |
| zeaxanthin | **568.4275** | 569.4353 |
| lutein | **568.4275** | 569.4353 |
| tunaxanthin | **568.4275** | 569.4353 |
| rhodoxanthin | 562.3806 | **563.3884** |
| canthaxanthin | 564.3962 | **565.4040** |
| beta-cryptoxanthin | **552.4326** | 553.4404 |
| alpha-carotene | **536.4376** | 537.4455 |
| beta-carotene | **536.4376** | 537.4455 |

Table S5. Transcript abundance in TPM (transcripts per million) of carotenoid color genes *bdh1l*, *ttc39b*, *scarb1* and *bco2*. Cichlid taxa are: TbR, *T.* sp. ‘black’ “Bulu Point” (red); TbY, *T.* sp. ‘black’ “Ikola” (yellow); TmR, *T. moorii* “Moliro” (red); TmY, *T. moorii* “Mbita” (yellow); AhR, *A. hansbaenschi* “Red Flush” (red); AbY, *A. baenschi* (yellow).

| taxon | color | sample ID | *bdh1l* | *ttc39b* | *scarb1* | *bco2* |
| --- | --- | --- | --- | --- | --- | --- |
| AhR | red | AhR1 | 4.509249 | 607.945007 | 216.43486 | 18.583916 |
| AhR | red | AhR2 | 8.512291 | 270.621155 | 92.140938 | 2.511071 |
| AhR | red | AhR3 | 4.807945 | 317.457825 | 111.735741 | 23.915342 |
| AhR | red | AhR4 | 10.006022 | 279.826294 | 70.111877 | 6.863055 |
| AhR | red | AhR5 | 2.173101 | 125.600403 | 40.318981 | 24.599138 |
| AhR | red | AhR6 | 9.434765 | 155.553894 | 1.215899 | 1.217815 |
| AbY | yellow | AbY1 | 0.307366 | 145.789948 | 80.879402 | 24.15971 |
| AbY | yellow | AbY2 | 0.079352 | 235.398605 | 45.448524 | 29.608442 |
| AbY | yellow | AbY3 | 1.709481 | 126.385452 | 2.601139 | 42.672276 |
| AbY | yellow | AbY4 | 3.118534 | 80.029099 | 0.785236 | 81.690674 |
| AbY | yellow | AbY5 | 0 | 135.456238 | 3.874367 | 226.406448 |
| AbY | yellow | AbY6 | 0 | 81.49958 | 0.966547 | 19.197567 |
| TbR | red | TbR1 | 20.407166 | 305.29071 | 150.353577 | 0.15613 |
| TbR | red | TbR2 | 39.834984 | 482.229889 | 201.733994 | 0.011349 |
| TbR | red | TbR3 | 22.72123 | 269.564819 | 192.296326 | 1.88521 |
| TbR | red | TbR4 | 51.02063 | 483.417816 | 345.477814 | 1.302176 |
| TbR | red | TbR5 | 26.688347 | 378.450409 | 270.956146 | 0.093871 |
| TbR | red | TbR6 | 39.972858 | 438.39743 | 232.792465 | 0.365113 |
| TbY | yellow | TbY1 | 1.612464 | 1.895389 | 3.608664 | 629.393005 |
| TbY | yellow | TbY2 | 0.09813 | 11.393731 | 115.992241 | 250.10733 |
| TbY | yellow | TbY3 | 1.970229 | 12.193412 | 108.81575 | 292.050354 |
| TbY | yellow | TbY4 | 1.352319 | 10.643957 | 89.977921 | 79.573494 |
| TbY | yellow | TbY5 | 1.524025 | 5.158847 | 58.677067 | 178.214188 |
| TbY | yellow | TbY6 | 0.13285 | 1.921346 | 3.296256 | 368.534515 |
| TmR | red | TmR1 | 17.930073 | 260.410339 | 137.734207 | 1.449869 |
| TmR | red | TmR2 | 15.086651 | 120.902626 | 119.958885 | 0 |
| TmR | red | TmR3 | 30.698503 | 272.694183 | 75.111183 | 0.713189 |
| TmR | red | TmR4 | 48.844635 | 464.992523 | 191.511627 | 0.155497 |
| TmR | red | TmR5 | 17.489328 | 307.030945 | 131.666306 | 0.179364 |
| TmR | red | TmR6 | 14.697319 | 215.359283 | 86.383095 | 0.538628 |
| TmY | yellow | TmY1 | 0.997251 | 20.919966 | 76.541512 | 8.353036 |
| TmY | yellow | TmY2 | 2.977584 | 240.600647 | 86.497971 | 1.500332 |
| TmY | yellow | TmY3 | 0.793792 | 29.729345 | 0.725255 | 9.233682 |
| TmY | yellow | TmY4 | 3.688969 | 67.813866 | 0.986849 | 4.166883 |
| TmY | yellow | TmY5 | 4.409012 | 80.231857 | 0 | 1.334572 |
| TmY | yellow | TmY6 | 1.099879 | 16.466667 | 0.492548 | 0.364251 |

**Figure S1.** HPLC chromatograms at 440 nm and 480 nm of the two carotenoid standard mixtures (mix1 and mix2).


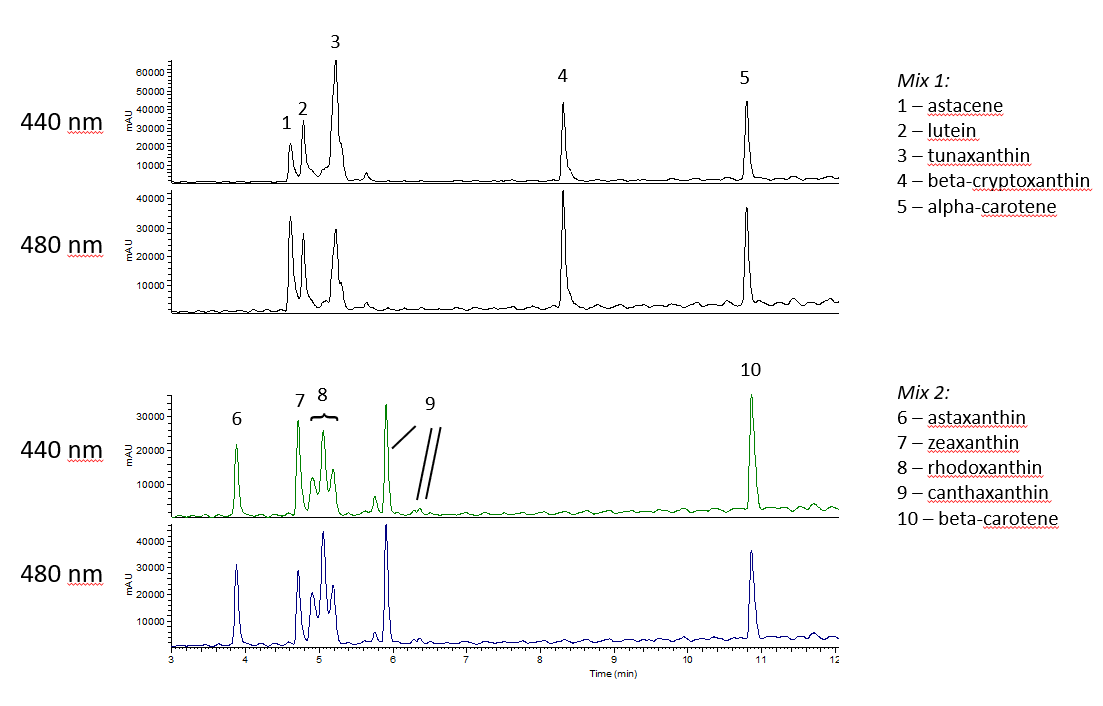


**Figure S2.** Example HPLC chromatograms. Black line: sample TbR135 (red skin); blue line: sample TbY148 (yellow skin). Chromatograms show HPLC signals at 480 nm before (upper) and after saponification (lower). Peaks of un-saponified extracts with retention times >8 min are carotenoid esters. The peak labeled with mass 549.4091 is the “unknown “carotenoid in Fig. 1 and Table S2.


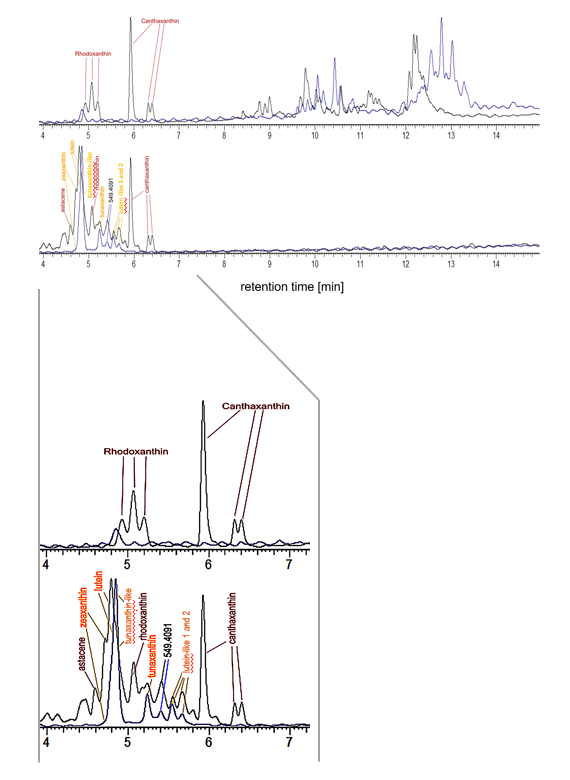


**Figure S3.** Hierarchical clustering of samples based on Euclidian distances between their carotenoid profiles. Carotenoid compounds were scored as 2, 1 and 0 for “present”, “present in traces” and “absent”, respectively.

**Figure S4.** Volcano plot of differentially expressed genes in the comparison between AbR and AhY; p.adjusted < 0.05, FC > 2.

**
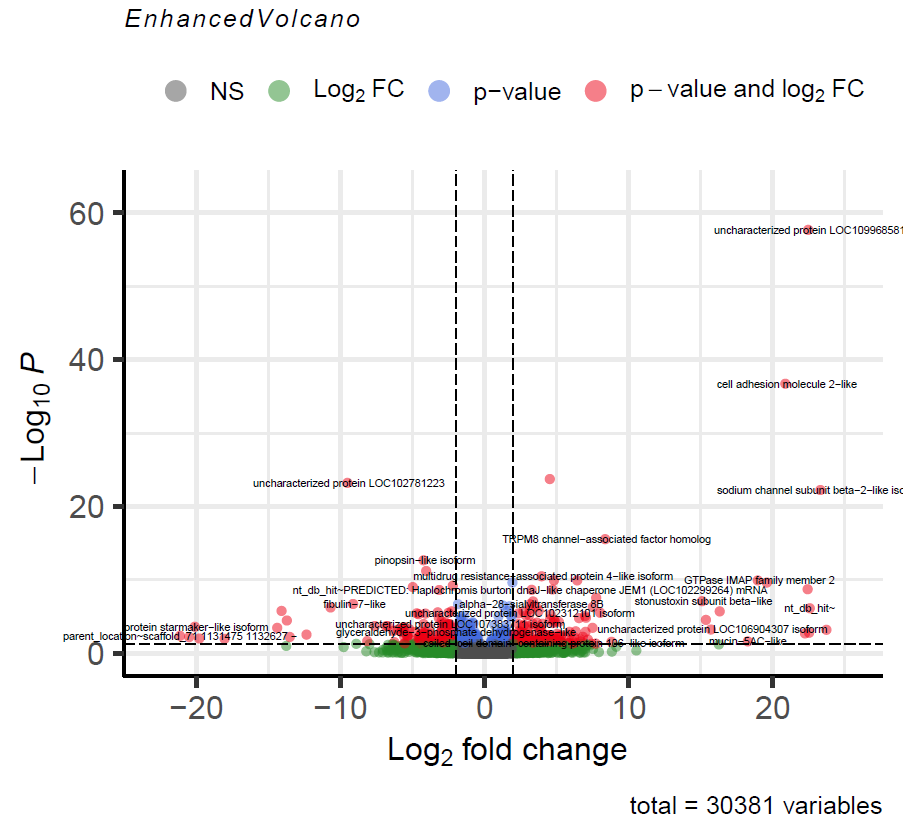
**

**Figure S5.** Volcano plot of differentially expressed genes in the comparison between TbR and TbY; p.adjusted < 0.05, FC > 2.

**
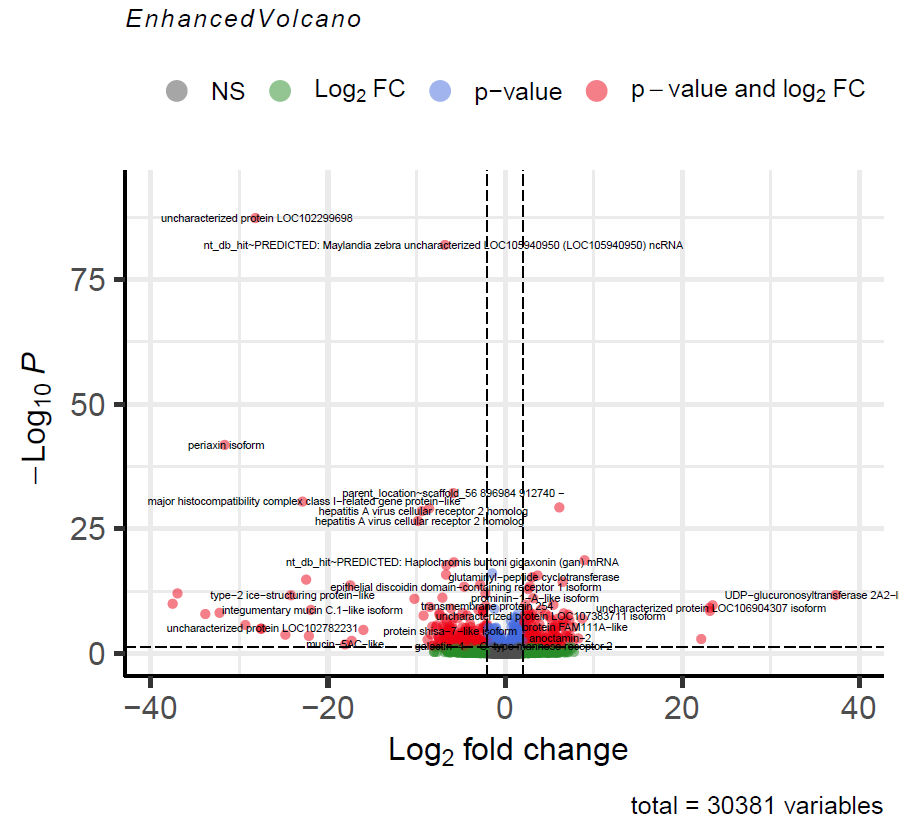
**

**Figure S6.** Volcano plot of differentially expressed genes in the comparison between TmR and TmY; p.adjusted < 0.05, FC > 2.

**
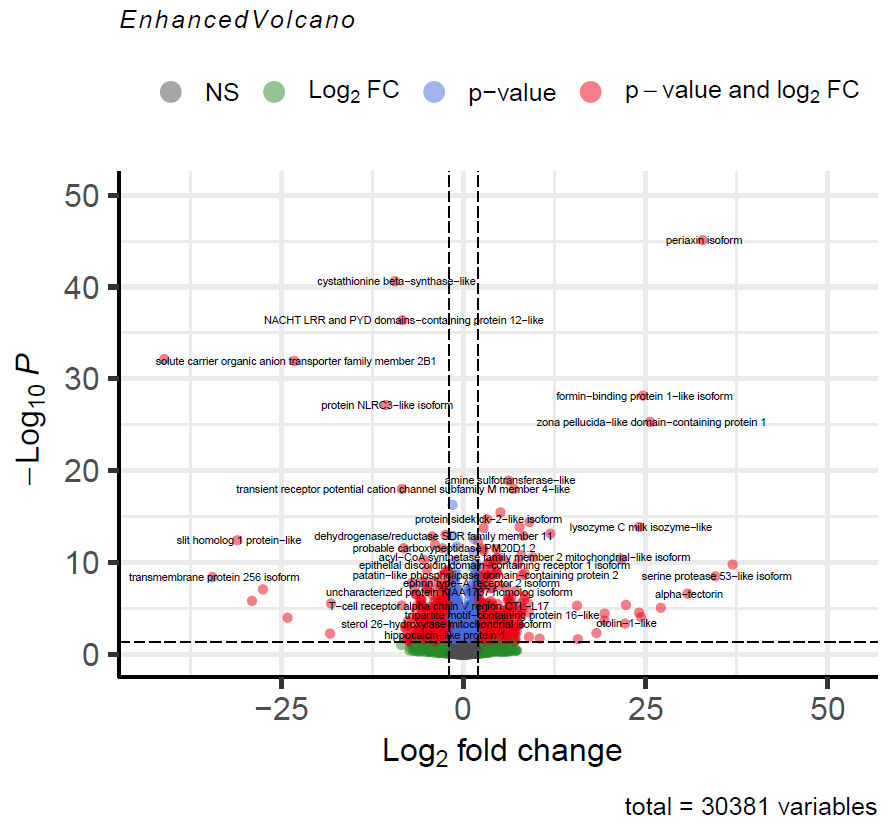
**

**Figure S7.** DNA and amino acid variation of *bdh1l*. Screenshots of colored amino acid sequence alignments, as well as amino acid and DNA sequence alignments in fasta format are provided. In some samples, transcript abundance of *bdh1l* was very low, resulting in incomplete or entirely failed assembly of the gene sequence.

The first two samples are the *T. moorii* reference (TM1 T0000032630-R1) and the *Metriaclima zebra* reference (ENSMZET00005027059).

***bdh1l*, amino acid alignment**


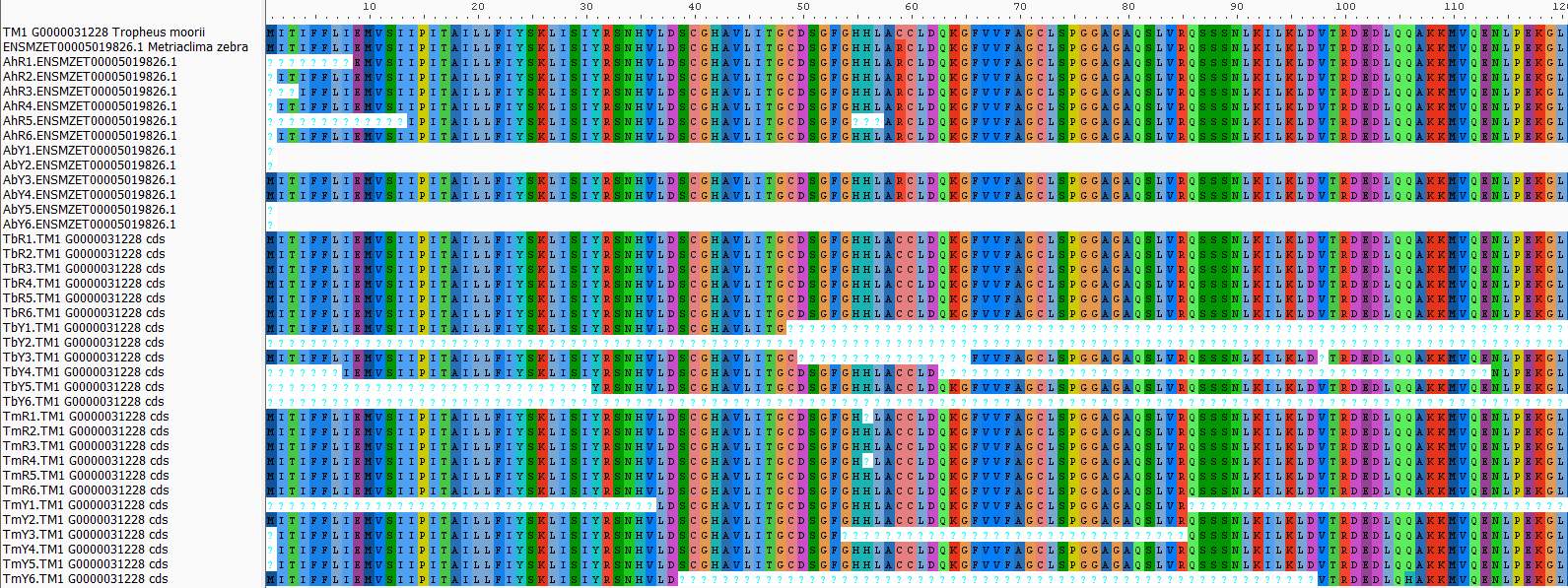


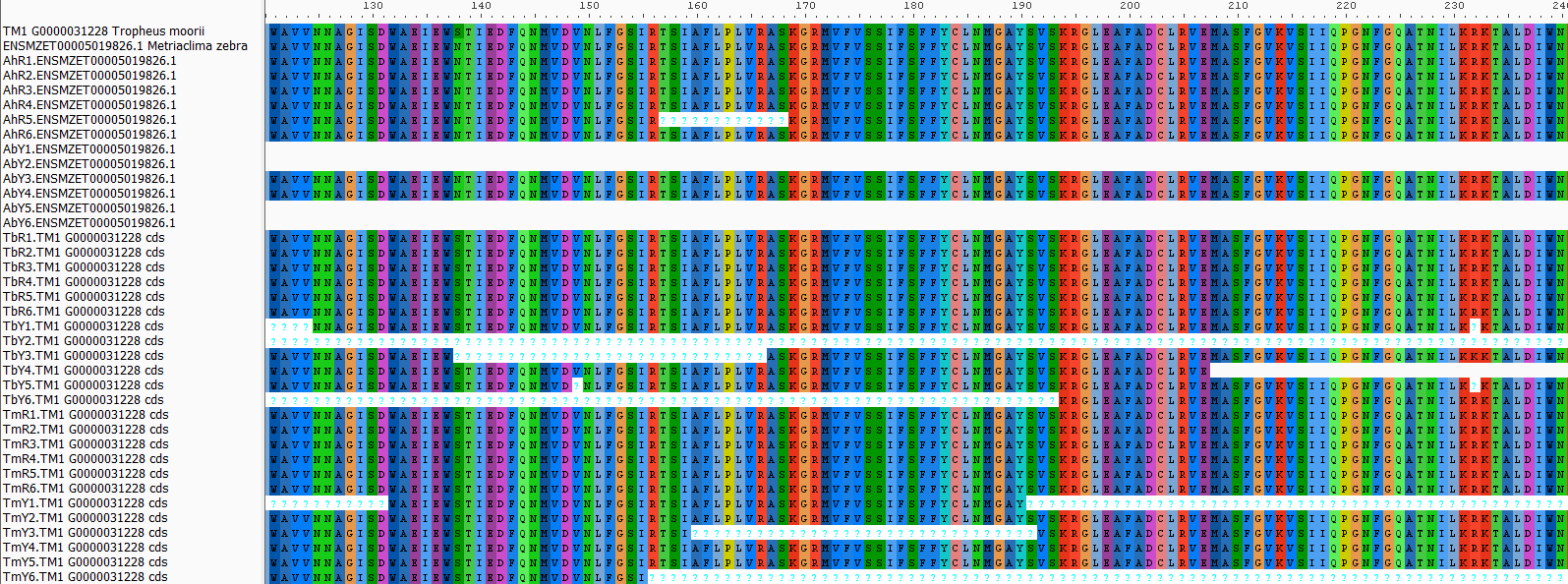


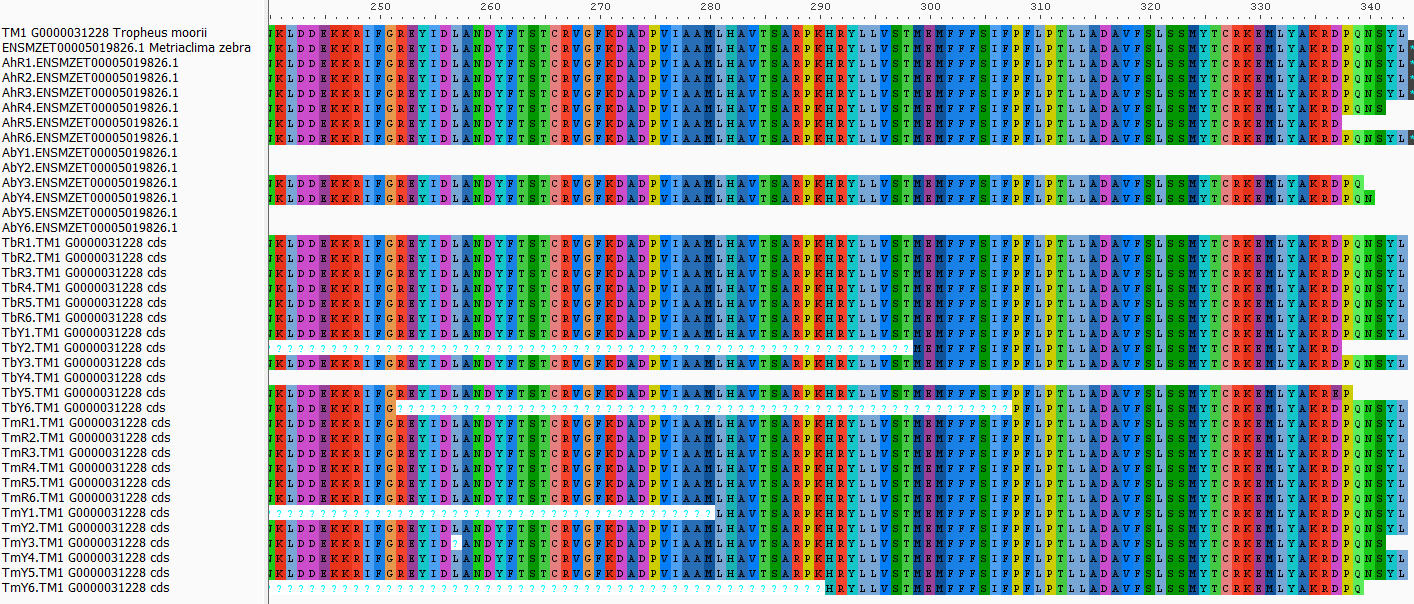


***bdh1l*, amino acid alignment in fasta format**

assembly failed for AbY1, AbY2, AbY5, AbY6 due to low transcript number

>TM1 G0000031228 Tropheus moorii

MITIFFLIEMVSIIPITAILLFIYSKLISIYRSNHVLDSCGHAVLITGCDSGFGHHLACCLDQKGFVVFAGCLSPGGAGAQSLVRQSSSNLKILKLDVTRDEDLQQAKKMVQENLPEKGLWAVVNNAGISDWAEIEWSTIEDFQNMVDVNLFGSIRTSIAFLPLVRASKGRMVFVSSIFSFFYCLNMGAYSVSKRGLEAFADCLRVEMASFGVKVSIIQPGNFGQATNILKRKTALDIWNKLDDEKKRIFGREYIDLANDYFTSTCRVGFKDADPVIAAMLHAVTSARPKHRYLLVSTMEMFFFSIFPFLPTLLADAVFSLSSMYTCRKEMLYAKRDPQNSYL

>ENSMZET00005019826.1 Metriaclima zebra

MITIFFLIEMVSIIPITAILLFIYSKLISIYRSNHVLDSCGHAVLITGCDSGFGHHLARCLDQKGFVVFAGCLSPGGAGAQSLVRQSSSNLKILKLDVTRDEDLQQAKKMVQENLPEKGLWAVVNNAGISDWAEIEWNTIEDFQNMVDVNLFGSIRTSIAFLPLVRASKGRMVFVSSIFSFFYCLNMGAYSVSKRGLEAFADCLRVEMASFGVKVSIIQPGNFGQATNILKRKTALDIWNKLDDEKKRIFGREYIDLANDYFTSTCRVGFKDADPVIAAMLHAVTSARPKHRYLLVSTMEMFFFSIFPFLPTLLADAVFSLSSMYTCRKEMLYAKRDPQNSYL*

>AhR1.ENSMZET00005019826.1

????????EMVSIIPITAILLFIYSKLISIYRSNHVLDSCGHAVLITGCDSGFGHHLARCLDQKGFVVFAGCLSPGGAGAQSLVRQSSSNLKILKLDVTRDEDLQQAKKMVQENLPEKGLWAVVNNAGISDWAEIEWNTIEDFQNMVDVNLFGSIRTSIAFLPLVRASKGRMVFVSSIFSFFYCLNMGAYSVSKRGLEAFADCLRVEMASFGVKVSIIQPGNFGQATNILKRKTALDIWNKLDDEKKRIFGREYIDLANDYFTSTCRVGFKDADPVIAAMLHAVTSARPKHRYLLVSTMEMFFFSIFPFLPTLLADAVFSLSSMYTCRKEMLYAKRDPQNSYL*

>AhR2.ENSMZET00005019826.1

?ITIFFLIEMVSIIPITAILLFIYSKLISIYRSNHVLDSCGHAVLITGCDSGFGHHLARCLDQKGFVVFAGCLSPGGAGAQSLVRQSSSNLKILKLDVTRDEDLQQAKKMVQENLPEKGLWAVVNNAGISDWAEIEWNTIEDFQNMVDVNLFGSIRTSIAFLPLVRASKGRMVFVSSIFSFFYCLNMGAYSVSKRGLEAFADCLRVEMASFGVKVSIIQPGNFGQATNILKRKTALDIWNKLDDEKKRIFGREYIDLANDYFTSTCRVGFKDADPVIAAMLHAVTSARPKHRYLLVSTMEMFFFSIFPFLPTLLADAVFSLSSMYTCRKEMLYAKRDPQNSYL*

>AhR3.ENSMZET00005019826.1

???IFFLIEMVSIIPITAILLFIYSKLISIYRSNHVLDSCGHAVLITGCDSGFGHHLARCLDQKGFVVFAGCLSPGGAGAQSLVRQSSSNLKILKLDVTRDEDLQQAKKMVQENLPEKGLWAVVNNAGISDWAEIEWNTIEDFQNMVDVNLFGSIRTSIAFLPLVRASKGRMVFVSSIFSFFYCLNMGAYSVSKRGLEAFADCLRVEMASFGVKVSIIQPGNFGQATNILKRKTALDIWNKLDDEKKRIFGREYIDLANDYFTSTCRVGFKDADPVIAAMLHAVTSARPKHRYLLVSTMEMFFFSIFPFLPTLLADAVFSLSSMYTCRKEMLYAKRDPQNSYL*

>AhR4.ENSMZET00005019826.1

?ITIFFLIEMVSIIPITAILLFIYSKLISIYRSNHVLDSCGHAVLITGCDSGFGHHLARCLDQKGFVVFAGCLSPGGAGAQSLVRQSSSNLKILKLDVTRDEDLQQAKKMVQENLPEKGLWAVVNNAGISDWAEIEWNTIEDFQNMVDVNLFGSIRTSIAFLPLVRASKGRMVFVSSIFSFFYCLNMGAYSVSKRGLEAFADCLRVEMASFGVKVSIIQPGNFGQATNILKRKTALDIWNKLDDEKKRIFGREYIDLANDYFTSTCRVGFKDADPVIAAMLHAVTSARPKHRYLLVSTMEMFFFSIFPFLPTLLADAVFSLSSMYTCRKEMLYAKRDPQNS

>AhR5.ENSMZET00005019826.1

?????????????IPITAILLFIYSKLISIYRSNHVLDSCGHAVLITGCDSGFG???ARCLDQKGFVVFAGCLSPGGAGAQSLVRQSSSNLKILKLDVTRDEDLQQAKKMVQENLPEKGLWAVVNNAGISDWAEIEWNTIEDFQNMVDVNLFGSIR????????????KGRMVFVSSIFSFFYCLNMGAYSVSKRGLEAFADCLRVEMASFGVKVSIIQPGNFGQATNILKRKTALDIWNKLDDEKKRIFGREYIDLANDYFTSTCRVGFKDADPVIAAMLHAVTSARPKHRYLLVSTMEMFFFSIFPFLPTLLADAVFSLSSMYTCRKEMLYAKRD

>AhR6.ENSMZET00005019826.1

?ITIFFLIEMVSIIPITAILLFIYSKLISIYRSNHVLDSCGHAVLITGCDSGFGHHLARCLDQKGFVVFAGCLSPGGAGAQSLVRQSSSNLKILKLDVTRDEDLQQAKKMVQENLPEKGLWAVVNNAGISDWAEIEWNTIEDFQNMVDVNLFGSIRTSIAFLPLVRASKGRMVFVSSIFSFFYCLNMGAYSVSKRGLEAFADCLRVEMASFGVKVSIIQPGNFGQATNILKRKTALDIWNKLDDEKKRIFGREYIDLANDYFTSTCRVGFKDADPVIAAMLHAVTSARPKHRYLLVSTMEMFFFSIFPFLPTLLADAVFSLSSMYTCRKEMLYAKRDPQNSYL*

>AbY3.ENSMZET00005019826.1

MITIFFLIEMVSIIPITAILLFIYSKLISIYRSNHVLDSCGHAVLITGCDSGFGHHLARCLDQKGFVVFAGCLSPGGAGAQSLVRQSSSNLKILKLDVTRDEDLQQAKKMVQENLPEKGLWAVVNNAGISDWAEIEWNTIEDFQNMVDVNLFGSIRTSIAFLPLVRASKGRMVFVSSIFSFFYCLNMGAYSVSKRGLEAFADCLRVEMASFGVKVSIIQPGNFGQATNILKRKTALDIWNKLDDEKKRIFGREYIDLANDYFTSTCRVGFKDADPVIAAMLHAVTSARPKHRYLLVSTMEMFFFSIFPFLPTLLADAVFSLSSMYTCRKEMLYAKRDPQ

>AbY4.ENSMZET00005019826.1

MITIFFLIEMVSIIPITAILLFIYSKLISIYRSNHVLDSCGHAVLITGCDSGFGHHLARCLDQKGFVVFAGCLSPGGAGAQSLVRQSSSNLKILKLDVTRDEDLQQAKKMVQENLPEKGLWAVVNNAGISDWAEIEWNTIEDFQNMVDVNLFGSIRTSIAFLPLVRASKGRMVFVSSIFSFFYCLNMGAYSVSKRGLEAFADCLRVEMASFGVKVSIIQPGNFGQATNILKRKTALDIWNKLDDEKKRIFGREYIDLANDYFTSTCRVGFKDADPVIAAMLHAVTSARPKHRYLLVSTMEMFFFSIFPFLPTLLADAVFSLSSMYTCRKEMLYAKRDPQN

>TbR1.TM1 G0000031228 cds

MITIFFLIEMVSIIPITAILLFIYSKLISIYRSNHVLDSCGHAVLITGCDSGFGHHLACCLDQKGFVVFAGCLSPGGAGAQSLVRQSSSNLKILKLDVTRDEDLQQAKKMVQENLPEKGLWAVVNNAGISDWAEIEWSTIEDFQNMVDVNLFGSIRTSIAFLPLVRASKGRMVFVSSIFSFFYCLNMGAYSVSKRGLEAFADCLRVEMASFGVKVSIIQPGNFGQATNILKRKTALDIWNKLDDEKKRIFGREYIDLANDYFTSTCRVGFKDADPVIAAMLHAVTSARPKHRYLLVSTMEMFFFSIFPFLPTLLADAVFSLSSMYTCRKEMLYAKRDPQNSYL

>TbR2.TM1 G0000031228 cds

MITIFFLIEMVSIIPITAILLFIYSKLISIYRSNHVLDSCGHAVLITGCDSGFGHHLACCLDQKGFVVFAGCLSPGGAGAQSLVRQSSSNLKILKLDVTRDEDLQQAKKMVQENLPEKGLWAVVNNAGISDWAEIEWSTIEDFQNMVDVNLFGSIRTSIAFLPLVRASKGRMVFVSSIFSFFYCLNMGAYSVSKRGLEAFADCLRVEMASFGVKVSIIQPGNFGQATNILKRKTALDIWNKLDDEKKRIFGREYIDLANDYFTSTCRVGFKDADPVIAAMLHAVTSARPKHRYLLVSTMEMFFFSIFPFLPTLLADAVFSLSSMYTCRKEMLYAKRDPQNSYL

>TbR3.TM1 G0000031228 cds

MITIFFLIEMVSIIPITAILLFIYSKLISIYRSNHVLDSCGHAVLITGCDSGFGHHLACCLDQKGFVVFAGCLSPGGAGAQSLVRQSSSNLKILKLDVTRDEDLQQAKKMVQENLPEKGLWAVVNNAGISDWAEIEWSTIEDFQNMVDVNLFGSIRTSIAFLPLVRASKGRMVFVSSIFSFFYCLNMGAYSVSKRGLEAFADCLRVEMASFGVKVSIIQPGNFGQATNILKRKTALDIWNKLDDEKKRIFGREYIDLANDYFTSTCRVGFKDADPVIAAMLHAVTSARPKHRYLLVSTMEMFFFSIFPFLPTLLADAVFSLSSMYTCRKEMLYAKRDPQNSYL

>TbR4.TM1 G0000031228 cds

MITIFFLIEMVSIIPITAILLFIYSKLISIYRSNHVLDSCGHAVLITGCDSGFGHHLACCLDQKGFVVFAGCLSPGGAGAQSLVRQSSSNLKILKLDVTRDEDLQQAKKMVQENLPEKGLWAVVNNAGISDWAEIEWSTIEDFQNMVDVNLFGSIRTSIAFLPLVRASKGRMVFVSSIFSFFYCLNMGAYSVSKRGLEAFADCLRVEMASFGVKVSIIQPGNFGQATNILKRKTALDIWNKLDDEKKRIFGREYIDLANDYFTSTCRVGFKDADPVIAAMLHAVTSARPKHRYLLVSTMEMFFFSIFPFLPTLLADAVFSLSSMYTCRKEMLYAKRDPQNSYL

>TbR5.TM1 G0000031228 cds

MITIFFLIEMVSIIPITAILLFIYSKLISIYRSNHVLDSCGHAVLITGCDSGFGHHLACCLDQKGFVVFAGCLSPGGAGAQSLVRQSSSNLKILKLDVTRDEDLQQAKKMVQENLPEKGLWAVVNNAGISDWAEIEWSTIEDFQNMVDVNLFGSIRTSIAFLPLVRASKGRMVFVSSIFSFFYCLNMGAYSVSKRGLEAFADCLRVEMASFGVKVSIIQPGNFGQATNILKRKTALDIWNKLDDEKKRIFGREYIDLANDYFTSTCRVGFKDADPVIAAMLHAVTSARPKHRYLLVSTMEMFFFSIFPFLPTLLADAVFSLSSMYTCRKEMLYAKRDPQNSYL

>TbR6.TM1 G0000031228 cds

MITIFFLIEMVSIIPITAILLFIYSKLISIYRSNHVLDSCGHAVLITGCDSGFGHHLACCLDQKGFVVFAGCLSPGGAGAQSLVRQSSSNLKILKLDVTRDEDLQQAKKMVQENLPEKGLWAVVNNAGISDWAEIEWSTIEDFQNMVDVNLFGSIRTSIAFLPLVRASKGRMVFVSSIFSFFYCLNMGAYSVSKRGLEAFADCLRVEMASFGVKVSIIQPGNFGQATNILKRKTALDIWNKLDDEKKRIFGREYIDLANDYFTSTCRVGFKDADPVIAAMLHAVTSARPKHRYLLVSTMEMFFFSIFPFLPTLLADAVFSLSSMYTCRKEMLYAKRDPQNSYL

>TbY1.TM1 G0000031228 cds

MITIFFLIEMVSIIPITAILLFIYSKLISIYRSNHVLDSCGHAVLITG????????????????????????????????????????????????????????????????????????????NNAGISDWAEIEWSTIEDFQNMVDVNLFGSIRTSIAFLPLVRASKGRMVFVSSIFSFFYCLNMGAYSVSKRGLEAFADCLRVEMASFGVKVSIIQPGNFGQATNILK?KTALDIWNKLDDEKKRIFGREYIDLANDYFTSTCRVGFKDADPVIAAMLHAVTSARPKHRYLLVSTMEMFFFSIFPFLPTLLADAVFSLSSMYTCRKEMLYAKRDPQNSYL

>TbY2.TM1 G0000031228 cds

??????????????????????????????????????????????????????????????????????????????????????????????????????????????????????????????????????????????????????????????????????????????????????????????????????????????????????????????????????????????????????????????????????????????????????????????????????????MEMFFFSIFPFLPTLLADAVFSLSSMYTCRKEMLYAKRD

>TbY3.TM1 G0000031228 cds

MITIFFLIEMVSIIPITAILLFIYSKLISIYRSNHVLDSCGHAVLITGC????????????????FVVFAGCLSPGGAGAQSLVRQSSSNLKILKLD?TRDEDLQQAKKMVQENLPEKGLWAVVNNAGISDWAEIEW?????????????????????????????ASKGRMVFVSSIFSFFYCLNMGAYSVSKRGLEAFADCLRVEMASFGVKVSIIQPGNFGQATNILKKKTALDIWNKLDDEKKRIFGREYIDLANDYFTSTCRVGFKDADPVIAAMLHAVTSARPKHRYLLVSTMEMFFFSIFPFLPTLLADAVFSLSSMYTCRKEMLYAKRDPQNSYL

>TbY4.TM1 G0000031228 cds

???????IEMVSIIPITAILLFIYSKLISIYRSNHVLDSCGHAVLITGCDSGFGHHLACCLD???????????????????????????????????????????????????NLPEKGLWAVVNNAGISDWAEIEWSTIEDFQNMVDVNLFGSIRTSIAFLPLVRASKGRMVFVSSIFSFFYCLNMGAYSVSKRGLEAFADCLRVE

>TbY5.TM1 G0000031228 cds

??????????????????????????????YRSNHVLDSCGHAVLITGCDSGFGHHLACCLDQKGFVVFAGCLSPGGAGAQSLVRQSSSNLKILKLDVTRDEDLQQAKKMVQENLPEKGLWAVVNNAGISDWAEIEWSTIEDFQNMVD?NLFGSIRTSIAFLPLVRASKGRMVFVSSIFSFFYCLNMGAYSVSKRGLEAFADCLRVEMASFGVKVSIIQPGNFGQATNILK?KTALDIWNKLDDEKKRIFGREYIDLANDYFTSTCRVGFKDADPVIAAMLHAVTSARPKHRYLLVSTMEMFFFSIFPFLPTLLADAVFSLSSMYTCRKEMLYAKREP

>TbY6.TM1 G0000031228 cds

?????????????????????????????????????????????????????????????????????????????????????????????????????????????????????????????????????????????????????????????????????????????????????????????????KRGLEAFADCLRVEMASFGVKVSIIQPGNFGQATNILKRKTALDIWNKLDDEKKRIFG????????????????????????????????????????????????????????PFLPTLLADAVFSLSSMYTCRKEMLYAKRDPQNSYL

>TmR1.TM1 G0000031228 cds

MITIFFLIEMVSIIPITAILLFIYSKLISIYRSNHVLDSCGHAVLITGCDSGFGH?LACCLDQKGFVVFAGCLSPGGAGAQSLVRQSSSNLKILKLDVTRDEDLQQAKKMVQENLPEKGLWAVVNNAGISDWAEIEWSTIEDFQNMVDVNLFGSIRTSIAFLPLVRASKGRMVFVSSIFSFFYCLNMGAYSVSKRGLEAFADCLRVEMASFGVKVSIIQPGNFGQATNILKRKTALDIWNKLDDEKKRIFGREYIDLANDYFTSTCRVGFKDADPVIAAMLHAVTSARPKHRYLLVSTMEMFFFSIFPFLPTLLADAVFSLSSMYTCRKEMLYAKRDPQNSYL

>TmR2.TM1 G0000031228 cds

MITIFFLIEMVSIIPITAILLFIYSKLISIYRSNHVLDSCGHAVLITGCDSGFGHHLACCLDQKGFVVFAGCLSPGGAGAQSLVRQSSSNLKILKLDVTRDEDLQQAKKMVQENLPEKGLWAVVNNAGISDWAEIEWSTIEDFQNMVDVNLFGSIRTSIAFLPLVRASKGRMVFVSSIFSFFYCLNMGAYSVSKRGLEAFADCLRVEMASFGVKVSIIQPGNFGQATNILKRKTALDIWNKLDDEKKRIFGREYIDLANDYFTSTCRVGFKDADPVIAAMLHAVTSARPKHRYLLVSTMEMFFFSIFPFLPTLLADAVFSLSSMYTCRKEMLYAKRDPQNSYL

>TmR3.TM1 G0000031228 cds

MITIFFLIEMVSIIPITAILLFIYSKLISIYRSNHVLDSCGHAVLITGCDSGFGHHLACCLDQKGFVVFAGCLSPGGAGAQSLVRQSSSNLKILKLDVTRDEDLQQAKKMVQENLPEKGLWAVVNNAGISDWAEIEWSTIEDFQNMVDVNLFGSIRTSIAFLPLVRASKGRMVFVSSIFSFFYCLNMGAYSVSKRGLEAFADCLRVEMASFGVKVSIIQPGNFGQATNILKRKTALDIWNKLDDEKKRIFGREYIDLANDYFTSTCRVGFKDADPVIAAMLHAVTSARPKHRYLLVSTMEMFFFSIFPFLPTLLADAVFSLSSMYTCRKEMLYAKRDPQNSYL

>TmR4.TM1 G0000031228 cds

MITIFFLIEMVSIIPITAILLFIYSKLISIYRSNHVLDSCGHAVLITGCDSGFGH?LACCLDQKGFVVFAGCLSPGGAGAQSLVRQSSSNLKILKLDVTRDEDLQQAKKMVQENLPEKGLWAVVNNAGISDWAEIEWSTIEDFQNMVDVNLFGSIRTSIAFLPLVRASKGRMVFVSSIFSFFYCLNMGAYSVSKRGLEAFADCLRVEMASFGVKVSIIQPGNFGQATNILKRKTALDIWNKLDDEKKRIFGREYIDLANDYFTSTCRVGFKDADPVIAAMLHAVTSARPKHRYLLVSTMEMFFFSIFPFLPTLLADAVFSLSSMYTCRKEMLYAKRDPQNSYL

>TmR5.TM1 G0000031228 cds

MITIFFLIEMVSIIPITAILLFIYSKLISIYRSNHVLDSCGHAVLITGCDSGFGHHLACCLDQKGFVVFAGCLSPGGAGAQSLVRQSSSNLKILKLDVTRDEDLQQAKKMVQENLPEKGLWAVVNNAGISDWAEIEWSTIEDFQNMVDVNLFGSIRTSIAFLPLVRASKGRMVFVSSIFSFFYCLNMGAYSVSKRGLEAFADCLRVEMASFGVKVSIIQPGNFGQATNILKRKTALDIWNKLDDEKKRIFGREYIDLANDYFTSTCRVGFKDADPVIAAMLHAVTSARPKHRYLLVSTMEMFFFSIFPFLPTLLADAVFSLSSMYTCRKEMLYAKRDPQNSYL

>TmR6.TM1 G0000031228 cds

MITIFFLIEMVSIIPITAILLFIYSKLISIYRSNHVLDSCGHAVLITGCDSGFGHHLACCLDQKGFVVFAGCLSPGGAGAQSLVRQSSSNLKILKLDVTRDEDLQQAKKMVQENLPEKGLWAVVNNAGISDWAEIEWSTIEDFQNMVDVNLFGSIRTSIAFLPLVRASKGRMVFVSSIFSFFYCLNMGAYSVSKRGLEAFADCLRVEMASFGVKVSIIQPGNFGQATNILKRKTALDIWNKLDDEKKRIFGREYIDLANDYFTSTCRVGFKDADPVIAAMLHAVTSARPKHRYLLVSTMEMFFFSIFPFLPTLLADAVFSLSSMYTCRKEMLYAKRDPQNSYL

>TmY1.TM1 G0000031228 cds

????????????????????????????????????LDSCGHAVLITGCDSGFGHHLACCLDQKGFVVFAGCLSPGGAGAQSLVR??????????????????????????????????????????????WAEIEWSTIEDFQNMVDVNLFGSIRTSIAFLPLVRASKGRMVFVSSIFSFFYCLNMGAY??????????????????????????????????????????????????????????????????????????????????????????LHAVTSARPKHRYLLVSTMEMFFFSIFPFLPTLLADAVFSLSSMYTCRKEMLYAKRDPQNSYL

>TmY2.TM1 G0000031228 cds

MITIFFLIEMVSIIPITAILLFIYSKLISIYRSNHVLDSCGHAVLITGCDSGFGHHLACCLDQKGFVVFAGCLSPGGAGAQSLVRQSSSNLKILKLDVTRDEDLQQAKKMVQENLPEKGLWAVVNNAGISDWAEIEWSTIEDFQNMVDVNLFGSIRTSIAFLPLVRASKGRMVFVSSIFSFFYCLNMGAYSVSKRGLEAFADCLRVEMASFGVKVSIIQPGNFGQATNILKRKTALDIWNKLDDEKKRIFGREYIDLANDYFTSTCRVGFKDADPVIAAMLHAVTSARPKHRYLLVSTMEMFFFSIFPFLPTLLADAVFSLSSMYTCRKEMLYAKRDPQNSYL

>TmY3.TM1 G0000031228 cds

?ITIFFLIEMVSIIPITAILLFIYSKLISIYRSNHVLDSCGHAVLITGCDSGF????????????????????????????????QSSSNLKILKLDVTRDEDLQQAKKMVQENLPEKGLWAVVNNAGISDWAEIEWSTIEDFQNMVDVNLFGSIRTSI????????????????????????????????VSKRGLEAFADCLRVEMASFGVKVSIIQPGNFGQATNILKRKTALDIWNKLDDEKKRIFGREYID?ANDYFTSTCRVGFKDADPVIAAMLHAVTSARPKHRYLLVSTMEMFFFSIFPFLPTLLADAVFSLSSMYTCRKEMLYAKRDPQNS

>TmY4.TM1 G0000031228 cds

?ITIFFLIEMVSIIPITAILLFIYSKLISIYRSNHVLDSCGHAVLITGCDSGFGHHLACCLDQKGFVVFAGCLSPGGAGAQSLVRQSSSNLKILKLDVTRDEDLQQAKKMVQENLPEKGLWAVVNNAGISDWAEIEWSTIEDFQNMVDVNLFGSIRTSIAFLPLVRASKGRMVFVSSIFSFFYCLNMGAYSVSKRGLEAFADCLRVEMASFGVKVSIIQPGNFGQATNILKRKTALDIWNKLDDEKKRIFGREYIDLANDYFTSTCRVGFKDADPVIAAMLHAVTSARPKHRYLLVSTMEMFFFSIFPFLPTLLADAVFSLSSMYTCRKEMLYAKRDPQNSYL

>TmY5.TM1 G0000031228 cds

?ITIFFLIEMVSIIPITAILLFIYSKLISIYRSNHVLDSCGHAVLITGCDSGFGHHLACCLDQKGFVVFAGCLSPGGAGAQSLVRQSSSNLKILKLDVTRDEDLQQAKKMVQENLPEKGLWAVVNNAGISDWAEIEWSTIEDFQNMVDVNLFGSIRTSIAFLPLVRASKGRMVFVSSIFSFFYCLNMGAYSVSKRGLEAFADCLRVEMASFGVKVSIIQPGNFGQATNILKRKTALDIWNKLDDEKKRIFGREYIDLANDYFTSTCRVGFKDADPVIAAMLHAVTSARPKHRYLLVSTMEMFFFSIFPFLPTLLADAVFSLSSMYTCRKEMLYAKRDPQNSYL

>TmY6.TM1 G0000031228 cds

MITIFFLIEMVSIIPITAILLFIYSKLISIYRSNHVLD???????????????????????????????????????????????????????????VTRDEDLQHAKKMVQENLPEKGLWAVVNNAGISDWAEIEWSTIEDFQNMVDVNLFGSI???????????????????????????????????????????????????????????????????????????????????????????????????????????????????????????????????????HRYLLVSTMEMFFFSIFPFLPTLLADAVFSLSSMYTCRKEMLYAKRDPQ

***bdh1l*, DNA sequence alignment in fasta format**

assembly failed for AbY1, AbY2, AbY5, AbY6 due to low transcript number

>TM1_G0000031228_Tropheus moorii

ATGATCACCATTTTCTTCCTCATCGAAATGGTCAGCATCATCCCCATCACTGCCATCCTCCTCTTCATCTATTCCAAGCTTATTTCCATCTACCGCTCCAACCATGTGTTGGACAGCTGTGGCCATGCTGTGCTGATAACAGGCTGTGACAGTGGCTTCGGGCACCACCTGGCTTGCTGTTTGGACCAAAAGGGGTTTGTGGTCTTTGCTGGGTGTTTGTCTCCAGGAGGAGCTGGAGCCCAGAGCCTGGTCAGACAGAGCTCCAGTAATCTGAAAATCCTCAAGTTGGATGTCACAAGGGATGAAGACTTGCAACAGGCAAAGAAAATGGTGCAGGAGAACCTGCCAGAGAAAGGTCTGTGGGCAGTTGTGAACAATGCTGGGATCTCAGACTGGGCCGAGATCGAATGGAGCACTATTGAAGATTTCCAAAACATGGTGGATGTTAACCTGTTTGGATCCATTAGGACATCTATCGCTTTCCTACCGCTGGTTCGTGCCAGCAAAGGTCGGATGGTTTTTGTGTCAAGCATCTTTTCCTTCTTCTACTGCTTGAACATGGGAGCATACAGTGTGTCAAAGAGAGGACTGGAGGCATTTGCTGACTGCTTAAGAGTGGAAATGGCTAGTTTTGGCGTGAAGGTCAGCATCATTCAGCCAGGTAATTTTGGCCAAGCCACTAACATCCTGAAGAGGAAAACTGCTTTGGACATCTGGAACAAACTGGATGATGAGAAAAAACGAATCTTTGGCAGAGAGTACATTGATCTGGCCAATGACTACTTCACGTCAACATGTAGGGTGGGATTCAAGGACGCCGATCCGGTCATCGCAGCAATGCTGCACGCGGTCACATCTGCTCGGCCGAAACACAGATACCTGCTGGTCTCCACCATGGAGATGTTTTTCTTCAGCATCTTCCCATTTCTGCCAACCCTCCTGGCTGATGCTGTGTTTTCTCTCAGCTCAATGTACACATGCAGAAAAGAAATGCTTTATGCTAAGCGAGACCCACAAAATAGCTATCTGT--

>ENSMZET00005019826.1_Metriaclima zebra

ATGATCACCATTTTCTTCCTCATCGAAATGGTCAGCATCATCCCCATCACTGCCATCCTCCTCTTCATCTATTCCAAGCTTATTTCCATCTACCGCTCCAACCATGTGTTGGACAGCTGTGGCCATGCTGTGCTGATAACAGGCTGTGACAGTGGCTTCGGGCACCACCTGGCTCGCTGTTTGGACCAAAAGGGGTTTGTGGTCTTTGCTGGGTGTTTGTCTCCAGGAGGAGCTGGAGCCCAGAGCCTGGTCAGACAGAGTTCCAGTAATCTAAAAATCCTCAAGTTGGATGTCACAAGGGATGAAGACTTGCAGCAGGCAAAGAAAATGGTGCAGGAGAACCTGCCAGAGAAAGGTCTGTGGGCAGTTGTGAACAATGCTGGGATCTCAGACTGGGCCGAGATCGAATGGAACACTATTGAAGATTTCCAAAACATGGTGGATGTTAACCTGTTTGGATCCATTAGGACATCTATCGCTTTCCTACCGCTGGTTCGTGCCAGCAAAGGTCGGATGGTTTTTGTGTCAAGTATCTTTTCCTTCTTCTACTGCTTGAACATGGGAGCATACAGTGTGTCAAAGAGAGGACTGGAGGCATTTGCTGACTGCTTAAGAGTGGAAATGGCTAGTTTTGGCGTGAAGGTCAGCATCATTCAGCCAGGTAATTTTGGCCAAGCCACTAACATCCTGAAGAGGAAAACTGCTTTGGACATCTGGAACAAACTGGATGATGAGAAAAAACGAATCTTTGGCAGAGAGTACATTGATCTGGCCAATGACTACTTCACGTCAACATGTAGGGTGGGATTCAAGGACGCCGATCCGGTCATCGCAGCAATGCTGCACGCGGTCACATCTGCTCGGCCGAAACACAGATACCTGCTGGTCTCCACCATGGAGATGTTTTTCTTCAGCATCTTCCCATTTCTGCCAACCCTCCTGGCTGATGCTGTGTTTTCTCTCAGCTCAATGTACACATGCAGAAAAGAAATGCTTTATGCTAAGCGAGACCCACAAAATAGCTATCTGTAA

>AhR1.ENSMZET00005019826.1

nnnnnnnnnnnnnnnnnnnnnnncgaaatggtcaGCATCATCCCCATCACTGCCATCCTCCTCTTCATCTATTCCAAGCTTATTTCCATCTACCGCTCCAACCATGTGTTGGACAGCTGTGGCCATGCTGTGCTGATAACAGGCTGTGACAGTGGCTTCGGGCACCACCTGGCTCGCTGTTTGGACCAAAAGGGGTTTGTGGTCTTTGCTGGGTGTTTGTCTCCAGGAGGAGCTGGAGCCCAGAGCCTGGTCAGACAGAGTTCCAGTAATCTAAAAATCCTCAAGTTGGATGTCACAAGGGATGAAGACTTGCAGCAGGCAAAGAAAATGGTGCAGGAGAACCTGCCAGAGAAAGGTCTGTGGGCAGTTGTGAACAATGCTGGGATCTCAGACTGGGCCGAGATCGAATGGAACACTATTGAAGATTTCCAAAACATGGTGGATGTTAACCTGTTTGGATCCATTAGGACATCTATCGCTTTCCTACCGCTGGTTCGTGCCAGCAAAGGTCGGATGGTTTTTGTGTCAAGTATCTTTTCCTTCTTCTACTGCTTGAACATGGGAGCATACAGTGTGTCAAAGAGAGGACTGGAGGCATTTGCTGACTGCTTAAGAGTGgaaatggctagttttggcgtgaaggtcagcatcattcagccaggtaattttGGCCAAGCCACTAACATCCTGAAGAGGAAAACTGCTTTGGACATCTGGAACAAACTGGATGATGAGAAAAAACGAATCTTTGGCAGAGAGTACATTGATCTGGCCAATGACTACTTCACGTCAACATGTAGGGTGGGATTCAAGGACGCCGATCCGGTCATCGCAGCAATGCTGCACGCGGTCACATCTGCTCGGCCGAAACACAGATACCTGCTGGTCTCCACCATGGAGATGTTTTTCTTCAGCATCTTCCCATTTCTGCCAACCCTCCTGGCTGATGCTGTGTTTTCTCTCAGCTCAATGTACACATGCAGAAAAGAAATGCTTTATGCTAAGCGAGACCCACAAAATAGCTATCTGtaa

>AhR2.ENSMZET00005019826.1

ntGATCACCATTTTCTTCCTCATCGAAATGGTCAGCATCATCCCCATCACTGCCATCCTCCTCTTCATCTATTCCAAGCTTATTTCCATCTACCGCTCCAACCATGTGTTGGACAGCTGTGGCCATGCTGTGCTGATAACAGGCTGTGACAGTGGCTTCGGGCACCACCTGGCTCGCTGTTTGGACCAAAAGGGGTTTGTGGTCTTTGCTGGGTGTTTGTCTCCAGGAGGAGCTGGAGCCCAGAGCCTGGTCAGACAGAGTTCCAGTAATCTAAAAATCCTCAAGTTGGATGTCACAAGGGATGAAGACTTGCAGCAGGCAAAGAAAATGGTGCAGGAGAACCTGCCAGAGAAAGGTCTGTGGGCAGTTGTGAACAATGCTGGGATCTCAGACTGGGCCGAGATCGAATGGAACACTATTGAAGATTTCCAAAACATGGTGGATGTTAACCTGTTTGGATCCATTAGGACATCTATCGCTTTCCTACCGCTGGTTCGTGCCAGCAAAGGTCGGATGGTTTTTGTGTCAAGTATCTTTTCCTTCTTCTACTGCTTGAACATGGGAGCATACAGTGTGTCAAAGAGAGGACTGGAGGCATTTGCTGACTGCTTAAGAGTGGAAATGGCTAGTTTTGGCGTGAAGGTCAGCATCATTCAGCCAGGTAATTTTGGCCAAGCCACTAACATCCTGAAGAGGAAAACTGCTTTGGACATCTGGAACAAACTGGATGATGAGAAAAAACGAATCTTTGGCAGAGAGTACATTGATCTGGCCAATGACTACTTCACGTCAACATGTAGGGTGGGATTCAAGGACGCCGATCCGGTCATCGCAGCAATGCTGCACGCGGTCACATCTGCTCGGCCGAAACACAGATACCTGCTGGTCTCCACCATGGAGATGTTTTTCTTCAGCATCTTCCCATTTCTGCCAACCCTCCTGGCTGATGCTGTGTTTTCTCTCAGCTCAATGTACACATGCAGAAAAGAAATGCTTTATGCTAAGCGAGACCCACAAAATAGCTATCtgtaa

>AhR3.ENSMZET00005019826.1

nnnnnnnnnattttcttcctcatcgaaatggtcagcatcatccccatcactgccatcctccTCTTCATCTATTCCAAGCTTATTTCCATCTACCGCTCCAACCATGTGTTGGACAGCTGTGGCCATGCTGTGCTGATAACAGGCTGTGACAGTGGCTTCGGGCACCACCTGGCTCGCTGTTTGGACCAAAAGGGGTTTGTGGTCTTTGCTGGGTGTTTGTCTCCAGGAGGAGCTGGAGCCCAGAGCCTGGTCAGACAGAGTTCCAGTAATCTAAAAATCCTCAAGTTGGATGTCACAAGGGATGAAGACTTGCAGCAGGCAAAGAAAATGGTGCAGGAGAACCTGCCAGAGAAAGGTCTGTGGGCAGTTGTGAACAATGCTGGGATCTCAGACTGGGCCGAGATCGAATGGAACACTATTGAAGATTTCCAAAACATGGTGGATGTTAACCTGTTTGGATCCATTAGGACATCTATCGCTTTCCTACCGCTGGTTCGTGCCAGCAAAGGTCGGATGGTTTTTGTGTCAAGTATCTTTTCCTTCTTCTACTGCTTGAACATGGGAGCATACAGTGTGTCAAAGAGAGGACTGGAGGCATTTGCTGACTGCTTAAGAGTGGAAATGGCTAGTTTTGGCGTGAAGGTCAGCATCATTCAGCCAGGTAATTTTGGCCAAGCCACTAACATCCTGAAGAGGAAAACTGCTTTGGACATCTGGAACAAACTGGATGATGAGAAAAAACGAATCTTTGGCAGAGAGTACATTGATCTGGCCAATGACTACTTCACGTCAACATGTAGGGTGGGATTCAAGGACGCCGATCCGGTCATCGCAGCAATGCTGCACGCGGTCACATCTGCTCGGCCGAAACACAGATACCTGCTGGTCTCCACCATGGAGATGTTTTTCTTCAGCATCTTCCCATTTCTGCCAACCCTCCTGGCTGATGCTGTGTTTTCTCTCAGCTCAATGTACACATGCAGAAAAGAAATGCTTTATGCTAAGCGAGACCCACAAAATAGCTATCtgtaa

>AhR4.ENSMZET00005019826.1

ntgatcaccatttTCTTCCTCATCGAAATGGTCAGCATCATCCCCATCACTGCCATCCTCCTCTTCATCTATTCCAAGCTTATTTCCATCTACCGCTCCAACCATGTGTTGGACAGCTGTGGCCATGCTGTGCTGATAACAGGCTGTGACAGTGGCTTCGGGCACCACCTGGCTCGCTGTTTGGACCAAAAGGGGTTTGTGGTCTTTGCTGGGTGTTTGTCTCCAGGAGGAGCTGGAGCCCAGAGCCTGGTCAGACAGAGTTCCAGTAATCTAAAAATCCTCAAGTTGGATGTCACAAGGGATGAAGACTTGCAGCAGGCAAAGAAAATGGTGCAGGAGAACCTGCCAGAGAAAGGTCTGTGGGCAGTTGTGAACAATGCTGGGATCTCAGACTGGGCCGAGATCGAATGGAACACTATTGAAGATTTCCAAAACATGGTGGATGTTAACCTGTTTGGATCCATTAGGACATCTATCGCTTTCCTACCGCTGGTTCGTGCCAGCAAAGGTCGGATGGTTTTTGTGTCAAGTATCTTTTCCTTCTTCTACTGCTTGAACATGGGAGCATACAGTGTGTCAAAGAGAGGACTGGAGGCATTTGCTGACTGCTTAAGAGTGGAAATGGCTAGTTTTGGCGTGAAGGTCAGCATCATTCAGCCAGGTAATTTTGGCCAAGCCACTAACATCCTGAAGAGGAAAACTGCTTTGGACATCTGGAACAAACTGGATGATGAGAAAAAACGAATCTTTGGCAGAGAGTACATTGATCTGGCCAATGACTACTTCACGTCAACATGTAGGGTGGGATTCAAGGACGCCGATCCGGTCATCGCAGCAATGCTGCACGCGGTCACATCTGCTCGGCCGAAACACAGATACCTGCTGGTCTCCACCATGGAGATGTTTTTCTTCAGCATCTTCCCATTTCTGCCAACCCTCCTGGCTGATGCTGTGTTTTCTCTCAGCTCAATGTACACATGCAGAAAAGAAATGCTTTATGCTAAGCGAGACCCACaaaatagc---------

>AhR5.ENSMZET00005019826.1

nnnnnnnnnnnnnnnnnnnnnnnnnnnnnnnnnnnnnncatccccatcactgccatcctcctcttcatctattccaagcttatttccatctaccgctccaaccatgtgttggacagctgtggccatgctgtgctgataacaggctgtgacagtggcttcgggcnnnnnnnngctcgctgtttggaccaaaaGGGGTTTGTGGTCTTTGCTGGGTGTTTGTCTCCAGGAGGAGCTGGAGCCCAGAGCCTGGTCAGACAGAGTTCCAGTAATCTAAAAATCCTCAAGTTGGATGTCACAAGGGATGAAGACTTGCAGCAGGCAAAGAAAATGGTGCAGGAGAACCTGCCAGAGAAAGGTCTGTGGGCAGTTGTGAACAATGCTGGGATCTCAGACTGGGCCGAGATCGAATGGAACACtattgaagatttccaaaacatggtggatgttaacctgtttggatccattaggacnnnnnnnnnnnnnnnnnnnnnnnnnnnnnnnnncaaaggtcggatggtttttgtgtcaagtatcttttccttcttctactgcttgaacatgggagcatacagtgtgtcaaagagaggactggaggcatttgctgactgcttaagaGTGGAAATGGCTAGTTTTGGCGTGAAGGTCAGCATCATTCAGCcaggtaattttggccaagccactaacatcctgaagaggaaaactgctttggacatctggaacaaaCTGGATGATGAGAAAAAacgaatctttggcagagagtacattgatctggccaatgactacttcacgtcaacatgtagggtgggaTTCAAGGACGCCGATCCGGTCATCGCAGCAATGCTGCACGCGGTCACATCTGCTCGGCCGAAACACAGATACCTGCTGGTCTCCACCATGGAGATGTTTTTCTTCAGCATCTTCCCATTTCTGCCAACCCTCCTGGCTGATGCTGTGTTTTCTCTCAGCTCAATGTACACATGCAGAAAAGAAATGCTTTATGCTAAGCGAGACc--------------------

>AhR6.ENSMZET00005019826.1

nnnatcaccatTTTCTTCCtCATCGAAATGGTCAGCATCATCCCCATCACTGCCATCCTCCTCTTCATCTATTCCAAGCTTATTTCCATCTACCGCTCCAACCATGTGTTGGACAGCTGTGGCCATGCTGTGCTGATAACAGGCTGTGACAGTGGCTTCGGGCACCACCTGGCTCGCTGTTTGGACCAAAAGGGGTTTGTGGTCTTTGCTGGGTGTTTGTCTCCAGGAGGAGCTGGAGCCCAGAGCCTGGTCAGACAGAGTTCCAGTAATCTAAAAATCCTCAAGTTGGATGTCACAAGGGATGAAGACTTGCAGCAGGCAAAGAAAATGGTGCAGGAGAACCTGCCAGAGAAAGGTCTGTGGGCAGTTGTGAACAATGCTGGGATCTCAGACTGGGCCGAGATCGAATGGAACACTATTGAAGATTTCCAAAACATGGTGGATGTTAACCTGTTTGGATCCATTAGGACATCTATCGCTTTCCTACCGCTGGTTCGTGCCAGCAAAGGTCGGATGGTTTTTGTGTCAAGTATCTTTTCCTTCTTCTACTGCTTGAACATGGGAGCATACAGTGTGTCAAAGAGAGGACTGGAGGCATTTGCTGACTGCTTAAGAGTGGAAATGGCTAGTTTTGGCGTGAAGGTCAGCATCATTCAGCCAGGTAATTTTGGCCAAGCCACTAACATCCTGAAGAGGAAAACTGCTTTGGACATCTGGAACAAACTGGATGATGAGAAAAAACGAATCTTTGGCAGAGAGTACATTGATCTGGCCAATGACTACTTCACGTCAACATGTAGGGTGGGATTCAAGGACGCCGATCCGGTCATCGCAGCAATGCTGCACGCGGTCACATCTGCTCGGCCGAAACACAGATACCTGCTGGTCTCCACCATGGAGATGTTTTTCTTCAGCATCTTCCCATTTCTGCCAACCCTCCTGGCTGATGCTGTGTTTTCTCTCAGCTCAATGTACACATGCAGAAAAGAAATGCTTTATGCTAAGCGAGACCCACAAAATAGCtatctgtaa

>AbY3.ENSMZET00005019826.1

atgatcaccATTTTCTTCCTCATCGAAATGGTCAGCATCATCCCCATCACTGCCATCCTCCTCTTCATCTATTCCAAGCTTATTTCCATCTACCGCTCCAACCATGTGTTGGACAGCTGTGGCCATGCTGTGCTGATAACAGGCTGTGACAGTGGCTTCGGGCACCACCTgGCTCGCTGTTTGgaccaaaaggggtttGTGGTCTTTGCTGGGTGTTTGTCTCCAGGAGGAGCTGGAGCCCAGAGCCtggtcagacagagttccagtaatcTAAAAATCCTCAAGTTGGATGTCACaagggatgaagacttgcagcaggcaaagaaaatggtgcaggagaacctgccagagaaaggtctgtgggcagttgtgaacaatgctgggatctcagactgggccgagatcgaatggaacactattgaagatttccaaaacatggtggatgttaacctgtttggatccattaggacatctatcgctttcctaccgctgGTTCGTGCCAGCAAAGGTCGGATGGTTTTTGTGTCAAGTATCTTTTCCTTCTTCTACTGCTTGAACATGGGAGCATACAGTGTGTCAAAGAGAGGACTGGAGGCATTTGCTGACTGCTTAAGAGTGgaaatggctagttttggcgtgaaggtcagcatcattcagccaggtaattttggccaagccaCTAACATCCTGAAGAGGAAAACTGCTTTGGACATCTGGAACAAACTGGATGATGAGAAAAAACGAATCTTTGGCAGAGAGTACATTGATCTGGCCAATGACTACTTCACGTCAACATGTAGGGTGGGATTCAAGgacgccgatccggtcatcgcagcaatgctgcacgcggtcacatctgctcggccgaaacacagatacctgCTGGTCTCCACCATGGAGATGTTTTTCTTCAGCATCTTCCCATTTCTGCCAACCCTCCTGGCTGATGCTGTGTTTTCTCTCAGCTCAATGTACACATGCAGAAAAGAAATgCTTTAtgctaagcgagacccaca----------------

>AbY4.ENSMZET00005019826.1

atgatcaccaTTTTCTTCCTCATCGAAATGGTCAGCATCATCCCCATCACTGCCATCCTCCTCTTCATCTATTCCAAGCTTATTTCCATCTACCGCTCCAACCATGTGTTGGACAGCTGTGGCCATGCTGTGCTGATAACAGGCTGTGACAGTGGCTTCGGGCACCACCTGGCTCGCTGTTTGGACCAAAAGGGGTTTGTGGTCTTTGCTGGGTGTTTGTCTCCAGGAGGAGCTGGAGCCCAGAGCCTGGTCAGACAGAGTTCCAGTAATCTAAAAATCCTCAAGTTGGATGTCACAAGGGATGAAGACTTGCAGCAGGCAAAGAAAATGGTGCAGGAGAACCTGCCAGAGAAAGGTCTGTGGGCAGTTGTGAACAATGCTGGGATCTCAGACTGGGCCGAGATCGAATGGAACACTATTGAAGATTTCCAAAACATGGTGGATGTTAACCTGTTTGGATCCATTAGGACATCTATCGCTTTCCTACCGCTGGTTCGTGCCAGCAAAGGTCGGATGGTTTTTGTGTCAAGTATCTTTTCCTTCTTCTACTGCTTGAACATGGGAGCATACAGTGTGTCAAAGAGAGGACTGGAGGCATTTGCTGACTGCTTAAGAGTGGAAATGGCTAGTTTTGGCGTGAAGGTCAGCATCATTCAGCCAGGTAATTTTGGCCAAGCCACTAACATCCTGAAGAGGAAAACTGCTTTGGACATCTGGAACAAACTGGATGATGAGAAAAAACGAATCTTTGGCAGAGAGTACATTGATCTGGCCAATGACTACTTCACGTCAACATGTAGGGTGGGATTCAAGGACGCCGATCCGGTCATCGCAGCAATGCTGCACGCGGTCACATCTGCTCGGCCGAAACACAGATACCTGCTGGTCTCCACCATGGAGATGTTTTTCTTCAGCATCTTCCCATTTCTGCCAACCCTCCTGGCTGATGCTGTGTTTTCTCTCAGCTCAATGTACACATGCAGAAAAGAAATGCTTTATGCTAAGCGAGACCCACaaaatag----------

>TbR1.TM1_G0000031228_cds

atgatcaccatTTTCTTCCTCATCGAAATGGTCAGCATCATCCCCATCACTGCCATCCTCCTCTTCATCTATTCCAAGCTTATTTCCATCTACCGCTCCAACCATGTGTTGGACAGCTGTGGCCATGCTGTGCTGATAACAGGCTGTGACAGTGGCTTCGGGCACCACCTGGCTTGCTGTTTGGACCAAAAGGGGTTTGTGGTCTTTGCTGGGTGTTTGTCTCCAGGAGGAGCTGGAGCCCAGAGCCTGGTCAGACAGAGCTCCAGTAATCTGAAAATCCTCAAGTTGGATGTCACAAGGGATGAAGACTTGCAACAGGCAAAGAAAATGGTGCAGGAGAACCTGCCAGAGAAAGGTCTGTGGGCAGTTGTGAACAATGCTGGGATCTCAGACTGGGCCGAGATCGAATGGAGCACTATTGAAGATTTCCAAAACATGGTGGATGTTAACCTGTTTGGATCCATTAGGACATCTATCGCTTTCCTACCGCTGGTTCGTGCCAGCAAAGGTCGGATGGTTTTTGTGTCAAGCATCTTTTCCTTCTTCTACTGCTTGAACATGGGAGCATACAGTGTGTCAAAGAGAGGACTGGAGGCATTTGCTGACTGCTTAAGAGTGGAAATGGCTAGTTTTGGCGTGAAGGTCAGCATCATTCAGCCAGGTAATTTTGGCCAAGCCACTAACATCCTGAAGAGGAAAACTGCTTTGGACATCTGGAACAAACTGGATGATGAGAAAAAACGAATCTTTGGCAGAGAGTACATTGATCTGGCCAATGACTACTTCACGTCAACATGTAGGGTGGGATTCAAGGACGCCGATCCGGTCATCGCAGCAATGCTGCACGCGGTCACATCTGCTCGGCCGAAACACAGATACCTGCTGGTCTCCACCATGGAGATGTTTTTCTTCAGCATCTTCCCATTTCTGCCAACCCTCCTGGCTGATGCTGTGTTTTCTCTCAGCTCAATGTACACATGCAGAAAAGAAATGCTTTATGCTAAGCGAGACCCACAAAATAGCTATCtgt--

>TbR2.TM1_G0000031228_cds

atgatcaccatttTCTTCCTCATCGAAATGGTCAGCATCATCCCCATCACTGCCATCCTCCTCTTCATCTATTCCAAGCTTATTTCCATCTACCGCTCCAACCATGTGTTGGACAGCTGTGGCCATGCTGTGCTGATAACAGGCTGTGACAGTGGCTTCGGGCACCACCTGGCTTGCTGTTTGGACCAAAAGGGGTTTGTGGTCTTTGCTGGGTGTTTGTCTCCAGGAGGAGCTGGAGCCCAGAGCCTGGTCAGACAGAGCTCCAGTAATCTGAAAATCCTCAAGTTGGATGTCACAAGGGATGAAGACTTGCAACAGGCAAAGAAAATGGTGCAGGAGAACCTGCCAGAGAAAGGTCTGTGGGCAGTTGTGAACAATGCTGGGATCTCAGACTGGGCCGAGATCGAATGGAGCACTATTGAAGATTTCCAAAACATGGTGGATGTTAACCTGTTTGGATCCATTAGGACATCTATCGCTTTCCTACCGCTGGTTCGTGCCAGCAAAGGTCGGATGGTTTTTGTGTCAAGCATCTTTTCCTTCTTCTACTGCTTGAACATGGGAGCATACAGTGTGTCAAAGAGAGGACTGGAGGCATTTGCTGACTGCTTAAGAGTGGAAATGGCTAGTTTTGGCGTGAAGGTCAGCATCATTCAGCCAGGTAATTTTGGCCAAGCCACTAACATCCTGAAGAGGAAAACTGCTTTGGACATCTGGAACAAACTGGATGATGAGAAAAAACGAATCTTTGGCAGAGAGTACATTGATCTGGCCAATGACTACTTCACGTCAACATGTAGGGTGGGATTCAAGGACGCCGATCCGGTCATCGCAGCAATGCTGCACGCGGTCACATCTGCTCGGCCGAAACACAGATACCTGCTGGTCTCCACCATGGAGATGTTTTTCTTCAGCATCTTCCCATTTCTGCCAACCCTCCTGGCTGATGCTGTGTTTTCTCTCAGCTCAATGTACACATGCAGAAAAGAAATGCTTTATGCTAAGCGAGACCCACAAAATAgctatctgt--

>TbR3.TM1_G0000031228_cds

atgatcaccatttTCTTCCTCATCGAAATGGTCAGCATCATCCCCATCACTGCCATCCTCCTCTTCATCTATTCCAAGCTTATTTCCATCTACCGCTCCAACCATGTGTTGGACAGCTGTGGCCATGCTGTGCTGATAACAGGCTGTGACAGTGGCTTCGGGCACCACCTGGCTTGCTGTTTGGACCAAAAGGGGTTTGTGGTCTTTGCTGGGTGTTTGTCTCCAGGAGGAGCTGGAGCCCAGAGCCTGGTCAGACAGAGCTCCAGTAATCTGAAAATCCTCAAGTTGGATGTCACAAGGGATGAAGACTTGCAACAGGCAAAGAAAATGGTGCAGGAGAACCTGCCAGAGAAAGGTCTGTGGGCAGTTGTGAACAATGCTGGGATCTCAGACTGGGCCGAGATCGAATGGAGCACTATTGAAGATTTCCAAAACATGGTGGATGTTAACCTGTTTGGATCCATTAGGACATCTATCGCTTTCCTACCGCTGGTTCGTGCCAGCAAAGGTCGGATGGTTTTTGTGTCAAGCATCTTTTCCTTCTTCTACTGCTTGAACATGGGAGCATACAGTGTGTCAAAGAGAGGACTGGAGGCATTTGCTGACTGCTTAAGAGTGGAAATGGCTAGTTTTGGCGTGAAGGTCAGCATCATTCAGCCAGGTAATTTTGGCCAAGCCACTAACATCCTGAAGAGGAAAACTGCTTTGGACATCTGGAACAAACTGGATGATGAGAAAAAACGAATCTTTGGCAGAGAGTACATTGATCTGGCCAATGACTACTTCACGTCAACATGTAGGGTGGGATTCAAGGACGCCGATCCGGTCATCGCAGCAATGCTGCACGCGGTCACATCTGCTCGGCCGAAACACAGATACCTGCTGGTCTCCACCATGGAGATGTTTTTCTTCAGCATCTTCCCATTTCTGCCAACCCTCCTGGCTGATGCTGTGTTTTCTCTCAGCTCAATGTACACATGCAGAAAAGAAATGCTTTATGCTAAGCGAGACCCACAAAAtagctatctgt--

>TbR4.TM1_G0000031228_cds

atgatcaccatttTCTTCCTCATCGAAATGGTCAGCATCATCCCCATCACTGCCATCCTCCTCTTCATCTATTCCAAGCTTATTTCCATCTACCGCTCCAACCATGTGTTGGACAGCTGTGGCCATGCTGTGCTGATAACAGGCTGTGACAGTGGCTTCGGGCACCACCTGGCTTGCTGTTTGGACCAAAAGGGGTTTGTGGTCTTTGCTGGGTGTTTGTCTCCAGGAGGAGCTGGAGCCCAGAGCCTGGTCAGACAGAGCTCCAGTAATCTGAAAATCCTCAAGTTGGATGTCACAAGGGATGAAGACTTGCAACAGGCAAAGAAAATGGTGCAGGAGAACCTGCCAGAGAAAGGTCTGTGGGCAGTTGTGAACAATGCTGGGATCTCAGACTGGGCCGAGATCGAATGGAGCACTATTGAAGATTTCCAAAACATGGTGGATGTTAACCTGTTTGGATCCATTAGGACATCTATCGCTTTCCTACCGCTGGTTCGTGCCAGCAAAGGTCGGATGGTTTTTGTGTCAAGCATCTTTTCCTTCTTCTACTGCTTGAACATGGGAGCATACAGTGTGTCAAAGAGAGGACTGGAGGCATTTGCTGACTGCTTAAGAGTGGAAATGGCTAGTTTTGGCGTGAAGGTCAGCATCATTCAGCCAGGTAATTTTGGCCAAGCCACTAACATCCTGAAGAGGAAAACTGCTTTGGACATCTGGAACAAACTGGATGATGAGAAAAAACGAATCTTTGGCAGAGAGTACATTGATCTGGCCAATGACTACTTCACGTCAACATGTAGGGTGGGATTCAAGGACGCCGATCCGGTCATCGCAGCAATGCTGCACGCGGTCACATCTGCTCGGCCGAAACACAGATACCTGCTGGTCTCCACCATGGAGATGTTTTTCTTCAGCATCTTCCCATTTCTGCCAACCCTCCTGGCTGATGCTGTGTTTTCTCTCAGCTCAATGTACACATGCAGAAAAGAAATGCTTTATGCTAAGCGAGACCCACAAAatagctatctgt--

>TbR5.TM1_G0000031228_cds

atgatcaccatTTTCTTCCTCATCGAAATGGTCAGCATCATCCCCATCACTGCCATCCTCCTCTTCATCTATTCCAAGCTTATTTCCATCTACCGCTCCAACCATGTGTTGGACAGCTGTGGCCATGCTGTGCTGATAACAGGCTGTGACAGTGGCTTCGGGCACCACCTGGCTTGCTGTTTGGACCAAAAGGGGTTTGTGGTCTTTGCTGGGTGTTTGTCTCCAGGAGGAGCTGGAGCCCAGAGCCTGGTCAGACAGAGCTCCAGTAATCTGAAAATCCTCAAGTTGGATGTCACAAGGGATGAAGACTTGCAACAGGCAAAGAAAATGGTGCAGGAGAACCTGCCAGAGAAAGGTCTGTGGGCAGTTGTGAACAATGCTGGGATCTCAGACTGGGCCGAGATCGAATGGAGCACTATTGAAGATTTCCAAAACATGGTGGATGTTAACCTGTTTGGATCCATTAGGACATCTATCGCTTTCCTACCGCTGGTTCGTGCCAGCAAAGGTCGGATGGTTTTTGTGTCAAGCATCTTTTCCTTCTTCTACTGCTTGAACATGGGAGCATACAGTGTGTCAAAGAGAGGACTGGAGGCATTTGCTGACTGCTTAAGAGTGGAAATGGCTAGTTTTGGCGTGAAGGTCAGCATCATTCAGCCAGGTAATTTTGGCCAAGCCACTAACATCCTGAAGAGGAAAACTGCTTTGGACATCTGGAACAAACTGGATGATGAGAAAAAACGAATCTTTGGCAGAGAGTACATTGATCTGGCCAATGACTACTTCACGTCAACATGTAGGGTGGGATTCAAGGACGCCGATCCGGTCATCGCAGCAATGCTGCACGCGGTCACATCTGCTCGGCCGAAACACAGATACCTGCTGGTCTCCACCATGGAGATGTTTTTCTTCAGCATCTTCCCATTTCTGCCAACCCTCCTGGCTGATGCTGTGTTTTCTCTCAGCTCAATGTACACATGCAGAAAAGAAATGCTTTATGCTAAGCGAGACCCACAAAATAGCTATCTGt--

>TbR6.TM1_G0000031228_cds

atgatcaccatttTCTTCCTCATCGAAATGGTCAGCATCATCCCCATCACTGCCATCCTCCTCTTCATCTATTCCAAGCTTATTTCCATCTACCGCTCCAACCATGTGTTGGACAGCTGTGGCCATGCTGTGCTGATAACAGGCTGTGACAGTGGCTTCGGGCACCACCTGGCTTGCTGTTTGGACCAAAAGGGGTTTGTGGTCTTTGCTGGGTGTTTGTCTCCAGGAGGAGCTGGAGCCCAGAGCCTGGTCAGACAGAGCTCCAGTAATCTGAAAATCCTCAAGTTGGATGTCACAAGGGATGAAGACTTGCAACAGGCAAAGAAAATGGTGCAGGAGAACCTGCCAGAGAAAGGTCTGTGGGCAGTTGTGAACAATGCTGGGATCTCAGACTGGGCCGAGATCGAATGGAGCACTATTGAAGATTTCCAAAACATGGTGGATGTTAACCTGTTTGGATCCATTAGGACATCTATCGCTTTCCTACCGCTGGTTCGTGCCAGCAAAGGTCGGATGGTTTTTGTGTCAAGCATCTTTTCCTTCTTCTACTGCTTGAACATGGGAGCATACAGTGTGTCAAAGAGAGGACTGGAGGCATTTGCTGACTGCTTAAGAGTGGAAATGGCTAGTTTTGGCGTGAAGGTCAGCATCATTCAGCCAGGTAATTTTGGCCAAGCCACTAACATCCTGAAGAGGAAAACTGCTTTGGACATCTGGAACAAACTGGATGATGAGAAAAAACGAATCTTTGGCAGAGAGTACATTGATCTGGCCAATGACTACTTCACGTCAACATGTAGGGTGGGATTCAAGGACGCCGATCCGGTCATCGCAGCAATGCTGCACGCGGTCACATCTGCTCGGCCGAAACACAGATACCTGCTGGTCTCCACCATGGAGATGTTTTTCTTCAGCATCTTCCCATTTCTGCCAACCCTCCTGGCTGATGCTGTGTTTTCTCTCAGCTCAATGTACACATGCAGAAAAGAAATGCTTTATGCTAAGCGAGACCCACAAAatagctatctgt--

>TbY1.TM1_G0000031228_cds

atgatcaccattttcttcctcatcgaaatggtcagcatcatccccatcactgccatcctcctcttcatctattccaagcttatttccatctaccgctccaaccatgtgttggacagctgtggccatgctgtgctgataacaggcnnnnnnnnnnnnnnnnnnnnnnnnnnnnnnnnnnnnnnnnnnnnnnnnnnnnnnnnnnnnnnnnnnnnnnnnnnnnnnnnnnnnnnnnnnnnnnnnnnnnnnnnnnnnnnnnnnnnnnnnnnnnnnnnnnnnnnnnnnnnnnnnnnnnnnnnnnnnnnnnnnnnnnnnnnnnnnnnnnnnnnnnnnnnnnnnnnnnnnnnnnnnnnnnnnnnnnnnnnnnnnnnnnngaacaatgctgggatctcagactgggccgagaTCGAATGGAGCACTATTGAAGATTTCCAAAACATGGTGGATGTTAACTTGTTTGGATCCATTAGGACATCTATCGCTTTCCTACCGCTGGTTCGTGCCAGCAAAGGTCGGATGGTTTTTGTGTCAAGCATCTTTTCCTTCTTCTACTGCTTGAACATGGGAGCATACAGTGTGTCAAAGAGAGGACTGGAGGCATTTGCTGACTGCTTAAGAGTGGAAATGGCTAGTTTTGGCGTGAAGGTCAGCATCATTCAGCCAGGTAATTTTGGCCAAGCCACTAACATCCTGAAGARGAAAACTGCTTTGGACATCTGGAACAAACTGGATGATGAGAAAAAACGAATCTTTGGCAGAGAGTACATTGATCTGGCCAATGACTACTTCACGTCAACATGTAGGGTGGGATTCAAGGACGCCGATCCGGTCATCGCAGCAATGCTGCACGCGGTCACATCTGCTCGGCCGAAACACAGATACCtgctggtctccaccatggagatgtttttcttcagCATCTTcCCatttctgccaaccctcctggctgatgctgtgttttctctcagctcaatgtacacatgcagaaaagaaatgctttatgctaagcgagacccacaaaatagctatctgt--

>TbY2.TM1_G0000031228_cds

nnnnnnnnnnnnnnnnnnnnnnnnnnnnnnnnnnnnnnnnnnnnnnnnnnnnnnnnnnnnnnnnnnnnnnnnnnnnnnnnnnnnnnnnnnnnnnnnnnnnnnnnnnnnnnnnnnnnnnnnnnnnnnnnnnnnnnnnnnnnnnnnnnnnnnnnnnnnnnnnnnnnnnnnnnnnnnnnnnnnnnnnnnnnnnnnnnnnnnnnnnnnnnnnnnnnnnnnnnnnnnnnnnnnnnnnnnnnnnnnnnnnnnnnnnnnnnnnnnnnnnnnnnnnnnnnnnnnnnnnnnnnnnnnnnnnnnnnnnnnnnnnnnnnnnnnnnnnnnnnnnnnnnnnnnnnnnnnnnnnnnnnnnnnnnnnnnnnnnnnnnnnnnnnnnnnnnnnnnnnnnnnnnnnnnnnnnnnnnnnnnnnnnnnnnnnnnnnnnnnnnnnnnnnnnnnnnnnnnnnnnnnnnnnnnnnnnnnnnnnnnnnnnnnnnnnnnnnnnnnnnnnnnnnnnnnnnnnnnnnnnnnnnnnnnnnnnnnnnnnnnnnnnnnnnnnnnnnnnnnnnnnnnnnnnnnnnnnnnnnnnnnnnnnnnnnnnnnnnnnnnnnnnnnnnnnnnnnnnnnnnnnnnnnnnnnnnnnnnnnnnnnnnnnnnnnnnnnnnnnnnnnnnnnnnnnnnnnnnnnnnnnnnnnnnnnnnnnnnnnnnnnnnnnnnnnnnnnnnnnnnnnnnnnnnnnnnnnnnnnnnnnnnnnnnnnnnnnnnnnnnnnnnnnnnnnnnnnnnnnnnnnnnnnnnnnnnnnnnnnnnnnnnnnnnnnnnnnnnnnnnnnnnnnnnnnnnnnnnnnnnnnnnnnnnnnnnnnnnnnnnnnnnnnnnnnnnnnnnnnnnnnnnnnnnnnnnnnnnnnnnnnnnnnnnnnncatggagatgtttttcttcagcatcttcccatttctgccaaccctcctggctgatgctgtgttttctctcagctcaatgtacacatgcagaaaagaaatgctttatgctaagcgagac---------------------

>TbY3.TM1_G0000031228_cds

atgatcaccattttcttccTCATCGAAATGGTCAGCATCATCCCCATCACTGCCATCCTCCTCTTCATCTATTCCAAGCTTATTTCCATCTACCGCTCCAACCATGTGTTGGACAGCTGTGGCCATGCTGTGCTGATAACAGGCtgtgnnnnnnnnnnnnnnnnnnnnnnnnnnnnnnnnnnnnnnnnnnnnnggtttGTGGTCTTTGCTGGGTGTTTGTCTCCAGGAGGAGCTGGAGCCCAGAGCCTGGTCAGACAGAGCTCCAGTAATCTGAAAATCCTCAAGTTGGATGNCACAAGGGATGAAGACTTGCAACAGGCAAAGAAaatggtgcaggagaacctgccagagaaaggtctgtgggcagttgtgaacaatgctgggatctcagactgggccgagatcgaatggagnnnnnnnnnnnnnnnnnnnnnnnnnnnnnnnnnnnnnnnnnnnnnnnnnnnnnnnnnnnnnnnnnnnnnnnnnnnnnnnnnnnnngccagcaaaggtcggatggtttttgtgtCAAGCATCTTTTCCTTCTTCTACTGCTTGAACATGGGAGCATACAGTGTGTCAAAGAGAGGACTGGAGGCATTTGCTGACTGCTTAAGAGTGGAAATGGctagttttggcgtgaaggtcagcatcattcagccaggtaattttggccaagccactaACATCCTGAAGAAGAAAACTGCTTTGGACATCTGGAACAAACTGGATGATGAGAAAAAACGAATCTTTGGCAGAGAGTACATTGATCTGGCCAATGACTACTTCACGTCAACATGTAGGGTGGGATTCAAGGACGCCGATCCGGTCATCGCAGCAATGCTGCACGCGGTCACATCTGCTCGGCCGAAACACAGATACCTGCTGGTCTCCACCATGGAGATGTTTTTCTTCAGCATCTTCCCATTTCTGCCAACCCTCCTGGCTGATGCTGTGTTTTCTCTCAGCTCAATGTACACATGCAGAAAAGAAATGCTTTATGCTAAGCGAGACCCacaaaatagctatctgt--

>TbY4.TM1_G0000031228_cds

nnnnnnnnnnnnnnnnnnnnnatcgaaatggtcagcatcatccccatcactgccatcctcctcttcatctattccaagcttatttccatctaccgctccaaccatgtgttggacagctgtggccatgctgtgctgataacaggctgtgacagtggcttcgggcaccacctggcttgctgtttggacnnnnnnnnnnnnnnnnnnnnnnnnnnnnnnnnnnnnnnnnnnnnnnnnnnnnnnnnnnnnnnnnnnnnnnnnnnnnnnnnnnnnnnnnnnnnnnnnnnnnnnnnnnnnnnnnnnnnnnnnnnnnnnnnnnnnnnnnnnnnnnnnnnnnnnnngaacctgccagagaaaggtctgtgggcagttgtgaacaatgctgggatctcagactgggccgagatcgaatggagcactattgaagatttccaaaacatggtggatgttaacttgtttggatccattaggacatctatcgctttcctaccgctggttcgtgccagcaaaggtcggatggtttttgtgtcaagcatcttttccttcttctactgcttgaacatgggagcatacagtgtgtcaaagagaggactggaggcatttgctgactgcttaagagtgga----------------------------------------------------------------------------------------------------------------------------------------------------------------------------------------------------------------------------------------------------------------------------------------------------------------------------------------------------------------------------------------------------------------------------

>TbY5.TM1_G0000031228_cds

nnnnnnnnnnnnnnnnnnnnnnnnnnnnnnnnnnnnnnnnnnnnnnnnnnnnnnnnnnnnnnnnnnnnnnnnnnnnnnnnnnnnnnnnnctaccgctccaaccatgtgttggacagctgtggccatgctgtgctgataacaggctgtgacagtggcttcggGCACCACCTGGCTTGCTGTTTGGACCAAAAGGGGTTTGTGGTCTTTGCTGGGTGTTTGTCTCCAGGAGGAGCTGGAGCCCAGAGCCTGGTCAGACAGAGctccagtaatctgaaaatcctcaagttggatgtcacaagggatgaagacttgcaacaggcaaagaaaatggtgcaggagaacctgccagagaaaggtctgtgggcagttGTGAACAATGCTGGGATCTCAGACTGGGCCGAGATCGAATGGAGCACtattgaagattTCCAaaacatggtGGATNTTAACTTGTTTGGATCCATTAGGACATCTATCGCTTTCCTACCGCTGGTTCGTGCCAGCAAAGGTCGGATGGTTTTTGTGTCAAGCATCTTTTCCTTCTTCTACTGCTTGAACATGGGAGCATACAGTGTGTCAAAGAGAGGACTGGAGGCATTTGCTGACTGCTTAAGAGTGGAAATGGCTAGTTTTGGCGTGAAGGTCAGCATCATTCAGCCAGGTAATTTTGGCCAAGCCACTAACATCCTGAAGARGAAAACTGCTTTGGACATCTGGAACAAACTGGATGATGAGAAAAAACGAATCTTTGGCAGAGAGTACATTGATCTGGCCAATGACTACTTCACgtcaacatgtagggtgggattcaaggacgccgatccggtcatcgcagcaatgctgcacgcggtcacATCTGCtcggccgaaacacagatacctgctggtctccaccatggagatgtttttcttcagcatcttcccatttctgccaaccctcctggctgatgctgtgttttctctcagctcaatgtacacatgcagaaaagaaatgctttatgctaagcgagagccac-----------------

>TbY6.TM1_G0000031228_cds

nnnnnnnnnnnnnnnnnnnnnnnnnnnnnnnnnnnnnnnnnnnnnnnnnnnnnnnnnnnnnnnnnnnnnnnnnnnnnnnnnnnnnnnnnnnnnnnnnnnnnnnnnnnnnnnnnnnnnnnnnnnnnnnnnnnnnnnnnnnnnnnnnnnnnnnnnnnnnnnnnnnnnnnnnnnnnnnnnnnnnnnnnnnnnnnnnnnnnnnnnnnnnnnnnnnnnnnnnnnnnnnnnnnnnnnnnnnnnnnnnnnnnnnnnnnnnnnnnnnnnnnnnnnnnnnnnnnnnnnnnnnnnnnnnnnnnnnnnnnnnnnnnnnnnnnnnnnnnnnnnnnnnnnnnnnnnnnnnnnnnnnnnnnnnnnnnnnnnnnnnnnnnnnnnnnnnnnnnnnnnnnnnnnnnnnnnnnnnnnnnnnnnnnnnnnnnnnnnnnnnnnnnnnnnnnnnnnnnnnnnnnnnnnnnnnnnnnnnnnnnnnnnnnnnnnnnnnnnnnnnnnnnnnnnnnnnnnnnnnnnnnnnnnnnnnnnnnnnnnnnnnnnnnnnnnnnnnnnnnnnnnnnnnnnnnnnnnnnnnnnnnnnnnnnnnnnnnnncaaagagaggactggaggcatttgctgactgcttaagagtggaaatggctagttttggcgtgaaggtcagcatcattcagccaggtaattttggccaagccactaacatcctgaagaggaaaactgctttggacatctggaacaaactggatgatgagaaaaaacgaatctttggcnnnnnnnnnnnnnnnnnnnnnnnnnnnnnnnnnnnnnnnnnnnnnnnnnnnnnnnnnnnnnnnnnnnnnnnnnnnnnnnnnnnnnnnnnnnnnnnnnnnnnnnnnnnnnnnnnnnnnnnnnnnnnnnnnnnnnnnnnnnnnnnnnnnnnnnnnnnnnnnnnnnnnnncccatttctgccaaccctcctggctgatgctgtgttttctctcagctcaatgtacacatgcagaaaagaaatgctttatgctaagcgagacccacaaaatagctatctgt--

>TmR1.TM1_G0000031228_cds

atgatcaccatttTCTTCCTCATCGAAATGGTCAGCATCATCCCCATCACTGCCATCCTCCTCTTCATCTATTCCAAGCTTATTTCCATCTACCGCTCCAACCATGTGTTGGACAGCTGTGGCCATGCTGTGCTGATAACAGGCTGTGACAGTGGCTTCGGGCACCRCCTGGCTTGCTGTTTGGACCAAAAGGGGTTTGTGGTCTTTGCTGGGTGTTTGTCTCCAGGAGGAGCTGGAGCCCAGAGCCTGGTCAGACAGAGCTCCAGTAATCTGAAAATCCTCAAGTTGGATGTCACAAGGGATGAAGACTTGCAACAGGCAAAGAAAATGGTGCAGGAGAACCTGCCAGAGAAAGGTCTGTGGGCAGTTGTGAACAATGCTGGGATCTCAGACTGGGCTGAGATCGAATGGAGCACTATTGAAGATTTCCAAAACATGGTGGATGTTAACCTGTTTGGATCCATTAGGACATCTATCGCTTTCCTACCGCTGGTTCGTGCCAGCAAAGGTCGGATGGTTTTTGTGTCAAGCATCTTTTCCTTCTTCTACTGCTTGAACATGGGAGCATACAGTGTGTCAAAGAGAGGACTGGAGGCATTTGCTGACTGCTTAAGAGTGGAAATGGCTAGTTTTGGCGTGAAGGTCAGCATCATTCAGCCAGGTAATTTTGGCCAAGCCACTAACATCCTGAAGAGGAAAACTGCTTTGGACATCTGGAACAAACTGGATGATGAGAAAAAACGAATCTTTGGCAGAGAGTACATTGATCTGGCCAATGACTACTTCACGTCAACATGTAGGGTGGGATTCAAGGACGCCGATCCGGTCATCGCAGCAATGCTGCACGCGGTCACATCTGCTCGGCCGAAACACAGATACCTGCTGGTCTCCACCATGGAGATGTTTTTCTTCAGCATCTTCCCATTTCTGCCAACCCTCCTGGCTGATGCTGTGTTTTCTCTCAGCTCAATGTACACATGCAGAAAAGAAATGCTTTATGCTAAGCGAGACCCACAAAATAGCTATCtgt--

>TmR2.TM1_G0000031228_cds

atgatcaccatTTTCTTCCTCATCGAAATGGTCAGCATCATCCCCATCACTGCCATCCTCCTCTTCATCTATTCCAAGCTTATTTCCATCTACCGCTCCAACCATGTGTTGGACAGCTGTGGCCATGCTGTGCTGATAACAGGCTGTGACAGTGGCTTCGGGCACCACCTGGCTTGCTGTTTGGACCAAAAGGGGTTTGTGGTCTTTGCTGGGTGTTTGTCTCCAGGAGGAGCTGGAGCCCAGAGCCTGGTCAGACAGAGCTCCAGTAATCTGAAAATCCTCAAGTTGGATGTCACAAGGGATGAAGACTTGCAACAGGCAAAGAAAATGGTGCAGGAGAACCTGCCAGAGAAAGGTCTGTGGGCAGTTGTGAACAATGCTGGGATCTCAGACTGGGCTGAGATCGAATGGAGCACTATTGAAGATTTCCAAAACATGGTGGATGTTAACCTGTTTGGATCCATTAGGACATCTATCGCTTTCCTACCGCTGGTTCGTGCCAGCAAAGGTCGGATGGTTTTTGTGTCAAGCATCTTTTCCTTCTTCTACTGCTTGAACATGGGAGCATACAGTGTGTCAAAGAGAGGACTGGAGGCATTTGCTGACTGCTTAAGAGTGGAAATGGCTAGTTTTGGCGTGAAGGTCAGCATCATTCAGCCAGGTAATTTTGGCCAAGCCACTAACATCCTGAAGAGGAAAACTGCTTTGGACATCTGGAACAAACTGGATGATGAGAAAAAACGAATCTTTGGCAGAGAGTACATTGATCTGGCCAATGACTACTTCACGTCAACATGTAGGGTGGGATTCAAGGACGCCGATCCGGTCATCGCAGCAATGCTGCACGCGGTCACATCTGCTCGGCCGAAACACAGATACCTGCTGGTCTCCACCATGGAGATGTTTTTCTTCAGCATCTTCCCATTTCTGCCAACCCTCCTGGCTGATGCTGTGTTTTCTCTCAGCTCAATGTACACATGCAGAAAAGAAATGCTTTATGCTAAGCGAGACCCACAAAATAGCTAtctgt--

>TmR3.TM1_G0000031228_cds

atgatcaccatttTCTTCCTCATCGAAATGGTCAGCATCATCCCCATCACTGCCATCCTCCTCTTCATCTATTCCAAGCTTATTTCCATCTACCGCTCCAACCATGTGTTGGACAGCTGTGGCCATGCTGTGCTGATAACAGGCTGTGACAGTGGCTTCGGGCACCACCTGGCTTGCTGTTTGGACCAAAAGGGGTTTGTGGTCTTTGCTGGGTGTTTGTCTCCAGGAGGAGCTGGAGCCCAGAGCCTGGTCAGACAGAGCTCCAGTAATCTGAAAATCCTCAAGTTGGATGTCACAAGGGATGAAGACTTGCAACAGGCAAAGAAAATGGTGCAGGAGAACCTGCCAGAGAAAGGTCTGTGGGCAGTTGTGAACAATGCTGGGATCTCAGACTGGGCTGAGATCGAATGGAGCACTATTGAAGATTTCCAAAACATGGTGGATGTTAACCTGTTTGGATCCATTAGGACATCTATCGCTTTCCTACCGCTGGTTCGTGCCAGCAAAGGTCGGATGGTTTTTGTGTCAAGCATCTTTTCCTTCTTCTACTGCTTGAACATGGGAGCATACAGTGTGTCAAAGAGAGGACTGGAGGCATTTGCTGACTGCTTAAGAGTGGAAATGGCTAGTTTTGGCGTGAAGGTCAGCATCATTCAGCCAGGTAATTTTGGCCAAGCCACTAACATCCTGAAGAGGAAAACTGCTTTGGACATCTGGAACAAACTGGATGATGAGAAAAAACGAATCTTTGGCAGAGAGTACATTGATCTGGCCAATGACTACTTCACGTCAACATGTAGGGTGGGATTCAAGGACGCCGATCCGGTCATCGCAGCAATGCTGCACGCGGTCACATCTGCTCGGCCGAAACACAGATACCTGCTGGTCTCCACCATGGAGATGTTTTTCTTCAGCATCTTCCCATTTCTGCCAACCCTCCTGGCTGATGCTGTGTTTTCTCTCAGCTCAATGTACACATGCAGAAAAGAAATGCTTTATGCTAAGCGAGACCCACAAAATagctatctgt--

>TmR4.TM1_G0000031228_cds

atgatcaccatttTCTTCCTCATCGAAATGGTCAGCATCATCCCCATCACTGCCATCCTCCTCTTCATCTATTCCAAGCTTATTTCCATCTACCGCTCCAACCATGTGTTGGACAGCTGTGGCCATGCTGTGCTGATAACAGGCTGTGACAGTGGCTTCGGGCACCRCCTGGCTTGCTGTTTGGACCAAAAGGGGTTTGTGGTCTTTGCTGGGTGTTTGTCTCCAGGAGGAGCTGGAGCCCAGAGCCTGGTCAGACAGAGCTCCAGTAATCTGAAAATCCTCAAGTTGGATGTCACAAGGGATGAAGACTTGCAACAGGCAAAGAAAATGGTGCAGGAGAACCTGCCAGAGAAAGGTCTGTGGGCAGTTGTGAACAATGCTGGGATCTCAGACTGGGCTGAGATCGAATGGAGCACTATTGAAGATTTCCAAAACATGGTGGATGTTAACCTGTTTGGATCCATTAGGACATCTATCGCTTTCCTACCGCTGGTTCGTGCCAGCAAAGGTCGGATGGTTTTTGTGTCAAGCATCTTTTCCTTCTTCTACTGCTTGAACATGGGAGCATACAGTGTGTCAAAGAGAGGACTGGAGGCATTTGCTGACTGCTTAAGAGTGGAAATGGCTAGTTTTGGCGTGAAGGTCAGCATCATTCAGCCAGGTAATTTTGGCCAAGCCACTAACATCCTGAAGAGGAAAACTGCTTTGGACATCTGGAACAAACTGGATGATGAGAAAAAACGAATCTTTGGCAGAGAGTACATTGATCTGGCCAATGACTACTTCACGTCAACATGTAGGGTGGGATTCAAGGACGCCGATCCGGTCATCGCAGCAATGCTGCACGCGGTCACATCTGCTCGGCCGAAACACAGATACCTGCTGGTCTCCACCATGGAGATGTTTTTCTTCAGCATCTTCCCATTTCTGCCAACCCTCCTGGCTGATGCTGTGTTTTCTCTCAGCTCAATGTACACATGCAGAAAAGAAATGCTTTATGCTAAGCGAGACCCACAAAATAGCTATCtgt--

>TmR5.TM1_G0000031228_cds

atgatcaccatTTTCTTCCTCATCGAAATGGTCAGCATCATCCCCATCACTGCCATCCTCCTCTTCATCTATTCCAAGCTTATTTCCATCTACCGCTCCAACCATGTGTTGGACAGCTGTGGCCATGCTGTGCTGATAACAGGCTGTGACAGTGGCTTCGGGCACCACCTGGCTTGCTGTTTGGACCAAAAGGGGTTTGTGGTCTTTGCTGGGTGTTTGTCTCCAGGAGGAGCTGGAGCCCAGAGCCTGGTCAGACAGAGCTCCAGTAATCTGAAAATCCTCAAGTTGGATGTCACAAGGGATGAAGACTTGCAACAGGCAAAGAAAATGGTGCAGGAGAACCTGCCAGAGAAAGGTCTGTGGGCAGTTGTGAACAATGCTGGGATCTCAGACTGGGCTGAGATCGAATGGAGCACTATTGAAGATTTCCAAAACATGGTGGATGTTAACCTGTTTGGATCCATTAGGACATCTATCGCTTTCCTACCGCTGGTTCGTGCCAGCAAAGGTCGGATGGTTTTTGTGTCAAGCATCTTTTCCTTCTTCTACTGCTTGAACATGGGAGCATACAGTGTGTCAAAGAGAGGACTGGAGGCATTTGCTGACTGCTTAAGAGTGGAAATGGCTAGTTTTGGCGTGAAGGTCAGCATCATTCAGCCAGGTAATTTTGGCCAAGCCACTAACATCCTGAAGAGGAAAACTGCTTTGGACATCTGGAACAAACTGGATGATGAGAAAAAACGAATCTTTGGCAGAGAGTACATTGATCTGGCCAATGACTACTTCACGTCAACATGTAGGGTGGGATTCAAGGACGCCGATCCGGTCATCGCAGCAATGCTGCACGCGGTCACATCTGCTCGGCCGAAACACAGATACCTGCTGGTCTCCACCATGGAGATGTTTTTCTTCAGCATCTTCCCATTTCTGCCAACCCTCCTGGCTGATGCTGTGTTTTCTCTCAGCTCAATGTACACATGCAGAAAAGAAATGCTTTATGCTAAGCGAGACCCACAAAATAGCTATCtgt--

>TmR6.TM1_G0000031228_cds

atgatcaccatttTCTTCCTCATCGAAATGGTCAGCATCATCCCCATCACTGCCATCCTCCTCTTCATCTATTCCAAGCTTATTTCCATCTACCGCTCCAACCATGTGTTGGACAGCTGTGGCCATGCTGTGCTGATAACAGGCTGTGACAGTGGCTTCGGGCACCACCTGGCTTGCTGTTTGGACCAAAAGGGGTTTGTGGTCTTTGCTGGGTGTTTGTCTCCAGGAGGAGCTGGAGCCCAGAGCCTGGTCAGACAGAGCTCCAGTAATCTGAAAATCCTCAAGTTGGATGTCACAAGGGATGAAGACTTGCAACAGGCAAAGAAAATGGTGCAGGAGAACCTGCCAGAGAAAGGTCTGTGGGCAGTTGTGAACAATGCTGGGATCTCAGACTGGGCTGAGATCGAATGGAGCACTATTGAAGATTTCCAAAACATGGTGGATGTTAACCTGTTTGGATCCATTAGGACATCTATCGCTTTCCTACCGCTGGTTCGTGCCAGCAAAGGTCGGATGGTTTTTGTGTCAAGCATCTTTTCCTTCTTCTACTGCTTGAACATGGGAGCATACAGTGTGTCAAAGAGAGGACTGGAGGCATTTGCTGACTGCTTAAGAGTGGAAATGGCTAGTTTTGGCGTGAAGGTCAGCATCATTCAGCCAGGTAATTTTGGCCAAGCCACTAACATCCTGAAGAGGAAAACTGCTTTGGACATCTGGAACAAACTGGATGATGAGAAAAAACGAATCTTTGGCAGAGAGTACATTGATCTGGCCAATGACTACTTCACGTCAACATGTAGGGTGGGATTCAAGGACGCCGATCCGGTCATCGCAGCAATGCTGCACGCGGTCACATCTGCTCGGCCGAAACACAGATACCTGCTGGTCTCCACCATGGAGATGTTTTTCTTCAGCATCTTCCCATTTCTGCCAACCCTCCTGGCTGATGCTGTGTTTTCTCTCAGCTCAATGTACACATGCAGAAAAGAAATGCTTTATGCTAAGCGAGACCCACAAAATAGCTATCTgt--

>TmY1.TM1_G0000031228_cds

nnnnnnnnnnnnnnnnnnnnnnnnnnnnnnnnnnnnnnnnnnnnnnnnnnnnnnnnnnnnnnnnnnnnnnnnnnnnnnnnnnnnnnnnnnnnnnnnnnnnnnnnnnngttggacagctgtggccatgctgtgctgataacaggctgtgacagtggcttcgggcaccacctggcttgctgtttggaccaaaaggggtttgtggtctttgctgggtgtttgtctccaggaggagctggagcccagagcctggtcagacnnnnnnnnnnnnnnnnnnnnnnnnnnnnnnnnnnnnnnnnnnnnnnnnnnnnnnnnnnnnnnnnnnnnnnnnnnnnnnnnnnnnnnnnnnnnnnnnnnnnnnnnnnnnnnnnnnnnnnnnnnnnnnnnnnnnnnnnctgggccgagatcgaatggagcactattgaagatttccaaaacatggtggatgTTAACCTGTTTGGATCCATTAGGACATCTATCGCTTTCCTACCGCTGGTTCGTGCCAGCAAAGGTCGGATGGtttttgtgtcaagcatcttttccttcttctactgcttgaacatgggagcatacnnnnnnnnnnnnnnnnnnnnnnnnnnnnnnnnnnnnnnnnnnnnnnnnnnnnnnnnnnnnnnnnnnnnnnnnnnnnnnnnnnnnnnnnnnnnnnnnnnnnnnnnnnnnnnnnnnnnnnnnnnnnnnnnnnnnnnnnnnnnnnnnnnnnnnnnnnnnnnnnnnnnnnnnnnnnnnnnnnnnnnnnnnnnnnnnnnnnnnnnnnnnnnnnnnnnnnnnnnnnnnnnnnnnnnnnnnnnnnnnnnnnnnnnnnnnnnnnnnnnnnnnnnnnngctgcacgcggTCACATCTGCTCGGCCGAAACACAGATACCTGCTGGTCTCCACCATGGAGATGTTTTTCTTCAGCATCTTCCCATTTCTGCCAACCCTCCTGGCTGATGCTGTGTTTTCTCTCAGctcaatgtacacatgcagaaaagaaatgctttatgctaagcgagacccacaaaatagctatctg---

>TmY2.TM1_G0000031228_cds

atgatcaccattttcttcctcatcgaaaTGGTCAGCATCATCCCCATCACTGCCATCCTCCTCTTCATCTATTCCAAGCTTATTTCCATCTACCGCTCCAACCATGTGTTGGACAGCTGTGGCCATGCTGTGCTGATAACAGGCTGTGACAGTGGCTTCGGGCACCACCTGGCTTGCTGTTTGGACCAAAAGGGGTTTGTGGTCTTTGCTGGGTGTTTGTCTCCAGGAGGAGCTGGAGCCCAGAGCCTGGTCAGACAGAGCTCCAGTAATCTGAAAATCCTCAAGTTGGATGTCACAAGGGATGAAGACTTGCAACAGGCAAAGAAAATGGTGCAGGAGAACCTGCCAGAGAAAGGTCTGTGGGCAGTTGTGAACAATGCTGGGATCTCAGACTGGGCCGAGATCGAATGGAGCACTATTGAAGATTTCCAAAACATGGTGGATGTTAACCTGTTTGGATCCATTAGGACATCTATCGCTTTCCTACCGCTGGTTCGTGCCAGCAAAGGTCGGATGGTTTTTGTGTCAAGCATCTTTTCCTTCTTCTACTGCTTGAACATGGGAGCATACAGTGTGTCAAAGAGAGGACTGGAGGCATTTGCTGACTGCTTAAGAGTGGAAATGGCTAGTTTTGGCGTGAAGGTCAGCATCATTCAGCCAGGTAATTTTGGCCAAGCCACTAACATCCTGAAGAGGAAAACTGCTTTGGACATCTGGAACAAACTGGATGATGAGAAAAAACGAATCTTTGGCAGAGAGTACATTGATCTGGCCAATGACTACTTCACGTCAACATGTAGGGTGGGATTCAAGGACGCCGATCCGGTCATCGCAGCAATGCTGCACGCGGTCACATCTGCTCGGCCGAAACACAGATACCTGCTGGTCTCCACCATGGAGATGTTTTTCTTCAGCATCTTCCCATTTCTGCCAACCCTCCTGGCTGATGCTGTGTTTTCTCTCAGCTCAATGTACACATGCAGAAAAGAAATGCTTTATGCTAAGCGAGacccacaaaatagctatctgt--

>TmY3.TM1_G0000031228_cds

nngatcaccattttcttcctcatcgaaatggtcagcatcatccccatcactgccatcctcctcttcatctattccaagcttatttccatctaccgctccaaccatgtgttggacagctgtggccatgctgtgctgataacaggctgtgacagtggcttcggnnnnnnnnnnnnnnnnnnnnnnnnnnnnnnnnnnnnnnnnnnnnnnnnnnnnnnnnnnnnnnnnnnnnnnnnnnnnnnnnnnnnnnnnnnnnnncagagctccagtaatctgaaaatcctcaagttgGATGTCACAAGGGATGAAGACTTGCAACAGGCAAAGAAAATGGTGCAGGAGAACCTGCCAGAGAAAGGTCTGTGGGCAGTTGTGAACAATGCTGGGATCTCAGACTGGGCCGAGATCGAATggagcactattgaagatttccaaaacatggtggatgttaacctgtttggatccattaggacatctatcgcnnnnnnnnnnnnnnnnnnnnnnnnnnnnnnnnnnnnnnnnnnnnnnnnnnnnnnnnnnnnnnnnnnnnnnnnnnnnnnnnnnnnnnnnnnnngtgtgtcaaagagaggactggaggcatttgctgactgcttaagagtggaaatggctagttttggcgtgaaggtcagCATCATTCAGCCAGGTAATTTTGGCCAAGCCACTAACATCCTGAAGAGGAAAACTGCTTTGGACATCTGGAACAAACTGGATGATGAGAAAAAACGAATCTTTGGCAGAGAGTACATTGATCWGGccaatgactacttcacgtcaacatgtagggTGGGATTCAAGGacgccgatccggtcatcgcagCAATGCTGCACGCGGTCACATCTGCTCGGCCGAAACACAGATACCTGCTGGTCTCCACCATGGAGATGTTTTTCTTCAGCATCTTCCCATTTCTGCCAACCCTCCTGGCTGATGCTGTGTTTTCTCTCAGCTCAATGTACAcatgcagaaaagaaatgctttatgctaagcgagacccacaaaatagct--------

>TmY4.TM1_G0000031228_cds

ntgatcaccattttcttCCTCATCGAAATGGTCAGCATCATCCCCATCACTGCCATCCTCCTCTTCATCTATTCCAAGCTTATTTCCATCTACCGCTCCAACCATGTGTTGGACAGCTGTGGCCATGCTGTGCTGATAACAGGCTGTGACAGTGGCTTCGGGCACCACCTGGCTTGCTGTTTGGACCAAAAGGGGTTTGTGGTCTTTGCTGGGTGTTTGTCTCCAGGAGGAGCTGGAGCCCAGAGCCTGGTCAGACAGAGCTCCAGTAATCTGAAAATCCTCAAGTTGGATGTCACAAGGGATGAAGACTTGCAACAGGCAAAGAAAATGGTGCAGGAGAACCTGCCAGAGAAAGGTCTGTGGGCAGTTGTGAACAATGCTGGGATCTCAGACTGGGCCGAGATCGAATGGAGCACTATTGAAGATTTCCAAAACATGGTGGATGTTAACCTGTTTGGATCCATTAGGACATCTATCGCTTTCCTACCGCTGGTTCGTGCCAGCAAAGGTCGGATGGTTTTTGTGTCAAGCATCTTTTCCTTCTTCTACTGCTTGAACATGGGAGCATACAGTGTGTCAAAGAGAGGACTGGAGGCATTTGCTGACTGCTTAAGAGTGGAAATGGCTAGTTTTGGCGTGAAGGTCAGCATCATTCAGCCAGGTAATTTTGGCCAAGCCACTAACATCCTGAAGAGGAAAACTGCTTTGGACATCTGGAACAAACTGGATGATGAGAAAAAACGAATCTTTGGCAGAGAGTACATTGATCTGGCCAATGACTACTTCACGTCAACATGTAGGGTGGGATTCAAGGACGCCGATCCGGTCATCGCAGCAATGCTGCACGCGGTCACATCTGCTCGGCCGAAACACAGATACCTGCTGGTCTCCACCATGGAGATGTTTTTCTTCAGCATCTTCCCATTTCTGCCAACCCTCCTGGCTGATGCTGTGTTTTCTCTCAGCTCAATGTACACATGCAGAAAAGAAATGCTTTATGCTAAGCGAGACCCACAAAATAGctatctg---

>TmY5.TM1_G0000031228_cds

ntgatcaccatTTTCTTCCTCATCGAAATGGTCAGCATCATCCCCATCACTGCCATCCTCCTCTTCATCTATTCCAAGCTTATTTCCATCTACCGCTCCAACCATGTGTTGGACAGCTGTGGCCATGCTGTGCTGATAACAGGCTGTGACAGTGGCTTCGGGCACCACCTGGCTTGCTGTTTGGACCAAAAGGGGTTTGTGGTCTTTGCTGGGTGTTTGTCTCCAGGAGGAGCTGGAGCCCAGAGCCTGGTCAGACAGAGCTCCAGTAATCTGAAAATCCTCAAGTTGGATGTCACAAGGGATGAAGACTTGCAACAGGCAAAGAAAATGGTGCAGGAGAACCTGCCAGAGAAAGGTCTGTGGGCAGTTGTGAACAATGCTGGGATCTCAGACTGGGCCGAGATCGAATGGAGCACTATTGAAGATTTCCAAAACATGGTGGATGTTAACCTGTTTGGATCCATTAGGACATCTATCGCTTTCCTACCGCTGGTTCGTGCCAGCAAAGGTCGGATGGTTTTTGTGTCAAGCATCTTTTCCTTCTTCTACTGCTTGAACATGGGAGCATACAGTGTGTCAAAGAGAGGACTGGAGGCATTTGCTGACTGCTTAAGAGTGGAAATGGCTAGTTTTGGCGTGAAGGTCAGCATCATTCAGCCAGGTAATTTTGGCCAAGCCACTAACATCCTGAAGAGGAAAACTGCTTTGGACATCTGGAACAAACTGGATGATGAGAAAAAACGAATCTTTGGCAGAGAGTACATTGATCTGGCCAATGACTACTTCACGTCAACATGTAGGGTGGGATTCAAGGACGCCGATCCGGTCATCGCAGCAATGCTGCACGCGGTCACATCTGCTCGGCCGAAACACAGATACCTGCTGGTCTCCACCATGGAGATGTTTTTCTTCAGCATCTTCCCATTTCTGCCAACCCTCCTGGCTGATGCTGTGTTTTCTCTCAGCTCAATGTACACATGCAGAAAAGAAATGCTTTATGCTAAGCGAGACCCACAAAATAGCTATCtgt--

>TmY6.TM1_G0000031228_cds

atgatcaccattttcttcctcatcgaaatggtcagcatcatccccatcactgccatcctcctcttcatctattccaagcttatttccatctaccgctccaaccatgtgttggacnnnnnnnnnnnnnnnnnnnnnnnnnnnnnnnnnnnnnnnnnnnnnnnnnnnnnnnnnnnnnnnnnnnnnnnnnnnnnnnnnnnnnnnnnnnnnnnnnnnnnnnnnnnnnnnnnnnnnnnnnnnnnnnnnnnnnnnnnnnnnnnnnnnnnnnnnnnnnnnnnnnnnnnnnnnnnnnnngtcacaagggatgaagacttgcaacacgcaaagaaaatggtgcaggagaacctgccagagaaaggtctgtgggcagttgtgaacaatgctgggatctcagactgggccgagatcgaatggagcactattgaagatttccaaaacatggtggatgttaacctgtttggatccattagnnnnnnnnnnnnnnnnnnnnnnnnnnnnnnnnnnnnnnnnnnnnnnnnnnnnnnnnnnnnnnnnnnnnnnnnnnnnnnnnnnnnnnnnnnnnnnnnnnnnnnnnnnnnnnnnnnnnnnnnnnnnnnnnnnnnnnnnnnnnnnnnnnnnnnnnnnnnnnnnnnnnnnnnnnnnnnnnnnnnnnnnnnnnnnnnnnnnnnnnnnnnnnnnnnnnnnnnnnnnnnnnnnnnnnnnnnnnnnnnnnnnnnnnnnnnnnnnnnnnnnnnnnnnnnnnnnnnnnnnnnnnnnnnnnnnnnnnnnnnnnnnnnnnnnnnnnnnnnnnnnnnnnnnnnnnnnnnnnnnnnnnnnnnnnnnnnnnnnnnnnnnnnnnnnnnnnnnnnnnnnnnnnnnnnnnnnnnnnnnnaacacagatacctgctggtctccacCATGGAGATGTTTTTCTTCAGCATCTTCCCATTTCTGCCAACCCTCCTGGCTGATGCTGTGTTTTCTCTCAGCTCAATGTACACATGCAGAAAAGAAATGctttatgctaagcgagacccacaaa--------------

**Figure S8.** DNA and amino acid variation of *ttc39b.* Screenshots of colored amino acid sequence alignments, as well as amino acid and DNA sequence alignments in fasta format are provided.

The first two samples are the *T. moorii* reference (TM1 T0000032630-R1) and the *Metriaclima zebra* reference (ENSMZET00005027059).

***ttc39b*, amino acid alignment**

**
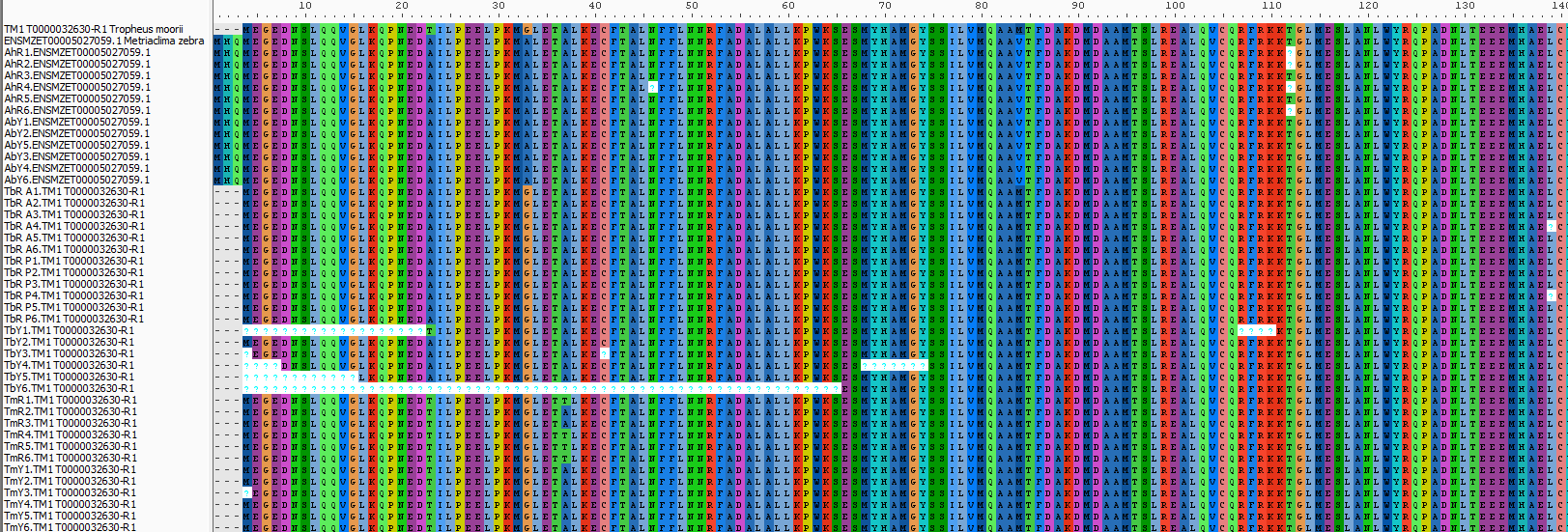
**


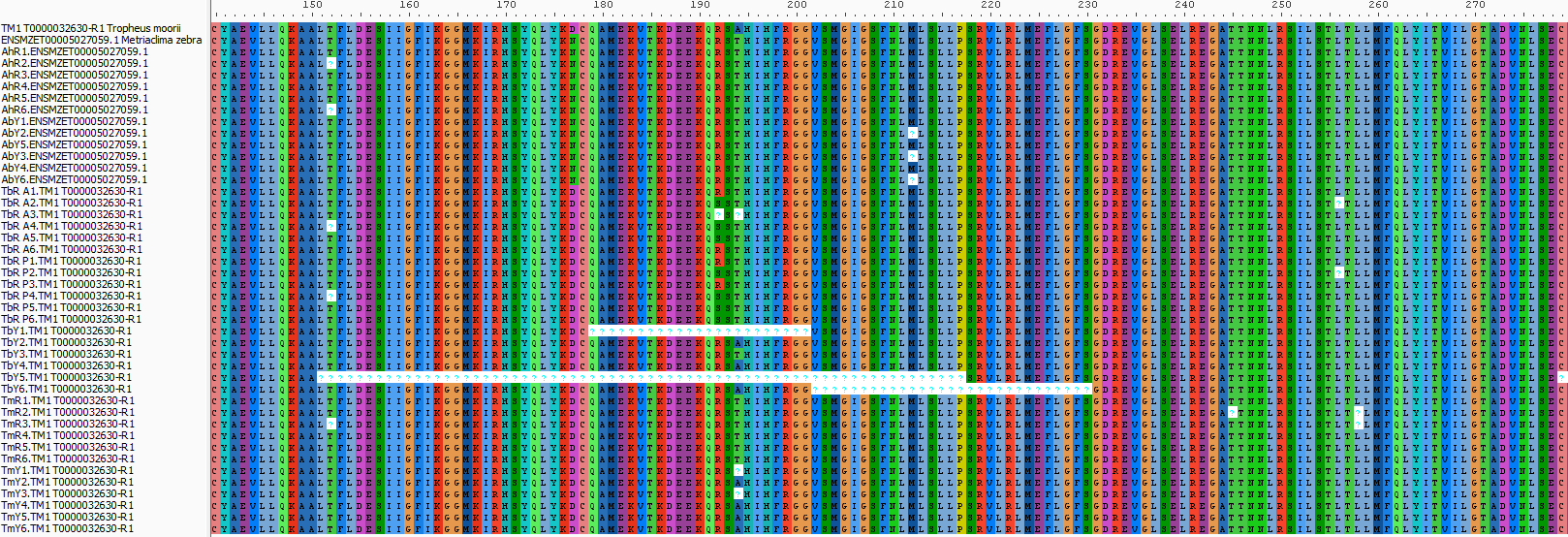


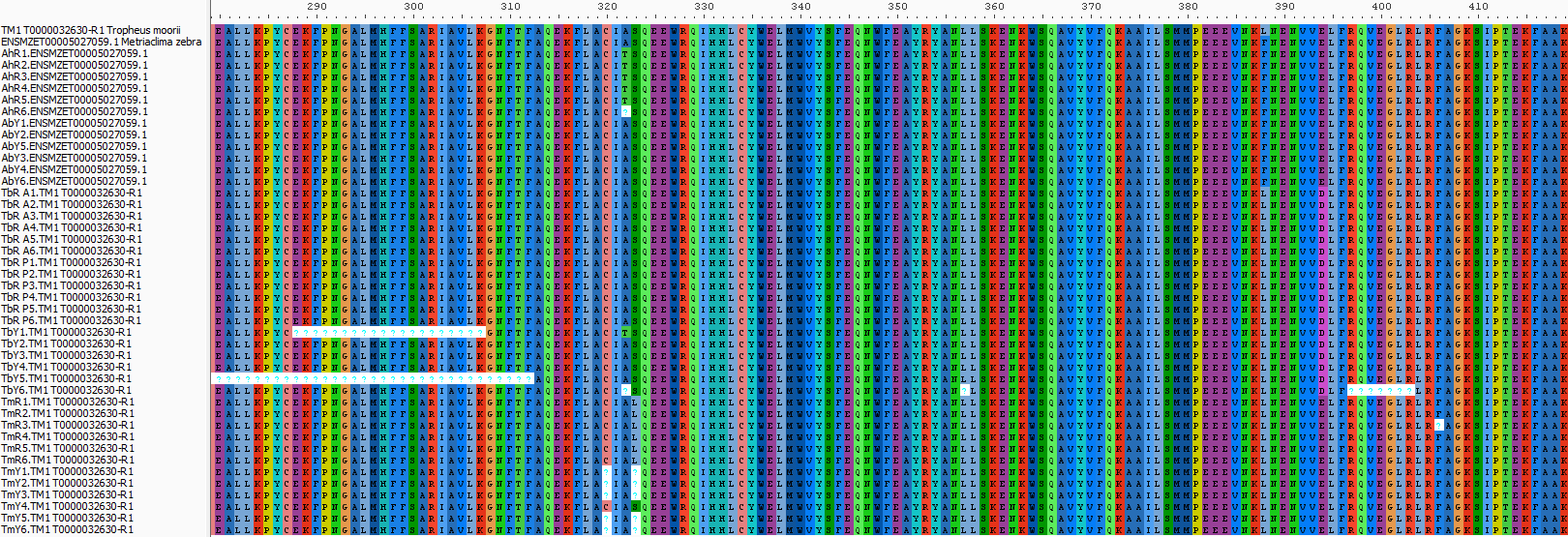


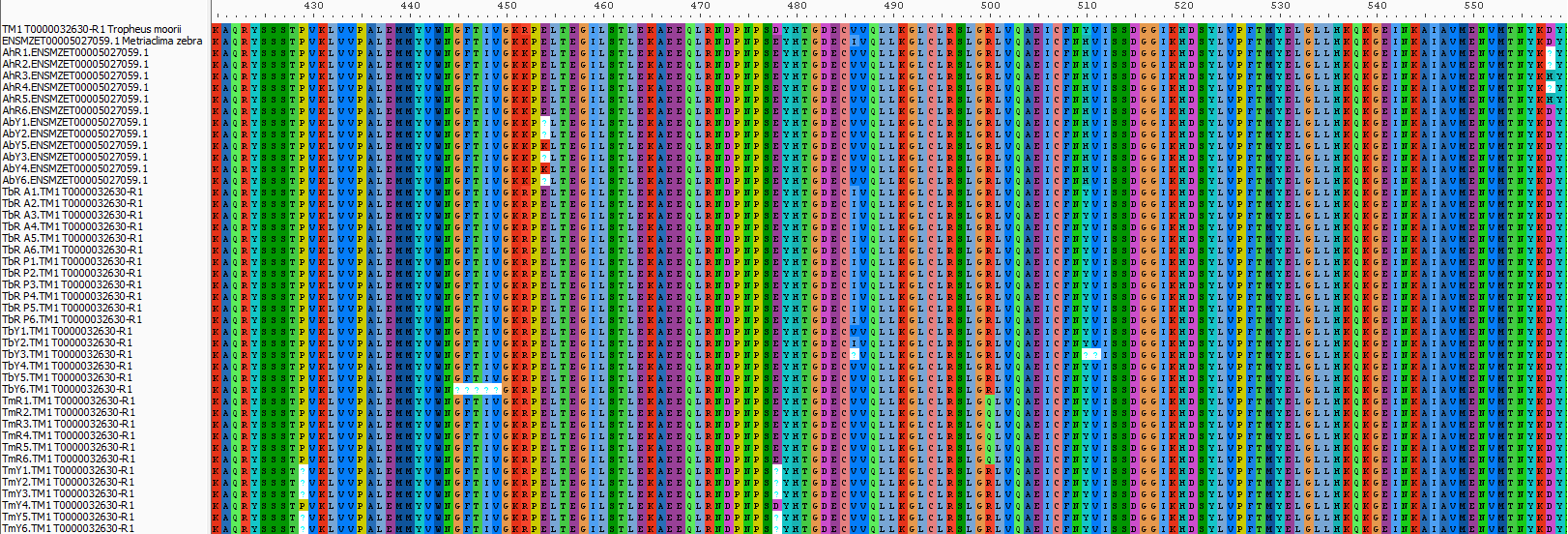


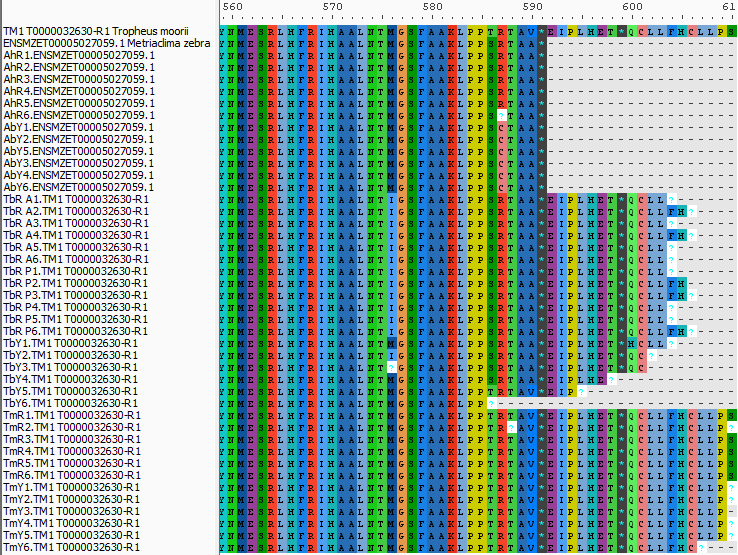


***ttc39b*, amino acid alignment in fasta format**

>TM1 T0000032630-R1 Tropheus moorii

---MEGEDNSLQQVGLKQPNEDTILPEELPKMGLETALKECFTALNFFLNNRFADALALLKPWKSESMYHAMGYSSILVMQAAMTFDAKDMDAAMTSLREALQVCQRFRKKTGLMESLANLWYRQPADNLTEEEMHAELCYAEVLLQKAALTFLDESIIGFIKGGMKIRHSYQLYKDCQAMEKVTKDEEKQRSAHIHFRGGVSMGIGSFNLMLSLLPSRVLRLMEFLGFSGDREVGLSELREGATTNNLRSILSTLTLLMFQLYITVILGTADVNLSECEALLKPYCEKFPNGALMHFFSARIAVLKGNFTFAQEKFLACIASQEEWRQIHHLCYWELMWVYSFEQNWFEAYRYANLLSKENKWSQAVYVFQKAAILSMMPEEEVNKLNENVVELFRQVEGLRLRFAGKSIPTEKFAAKKAQRYSSSTPVKLVVPALEMMYVWNGFTIVGKRPELTEGILSTLEKAEEQLRNDPNPSDYHTGDECVVQLLKGLCLRSLGRLVQAEICFNYVISSDGGIKHDSYLVPFTMYELGLLHKQKGEINKAIAVMENVMTNYKDYNMESRLHFRIHAALNTMGSFAAKLPPTRTAV*EIPLHET*QCLLFHCLLPS

>ENSMZET00005027059.1 Metriaclima zebra

MHQMEGEDNSLQQVGLKQPNEDAILPEELPKMALETALKECFTALNFFLNNRFADALALLKPWKSESMYHAMGYSSILVMQAAVTFDAKDMDAAMTSLREALQVCQRFRKKTGLMESLANLWYRQPADNLTEEEMHAELCYAEVLLQKAALTFLDESIIGFIKGGMKIRHSYQLYKNCQAMEKVTKDEEKQRSTHIHFRGGVSMGIGSFNLMLSLLPSRVLRLMEFLGFSGDREVGLSELREGATTNNLRSILSTLTLLMFQLYITVILGTADVNLSECEALLKPYCEKFPNGALMHFFSARIAVLKGNFTFAQEKFLACIASQEEWRQIHHLCYWELMWVYSFEQNWFEAYRYANLLSKENKWSQAVYVFQKAAILSMMPEEEVNKFNENVVELFRQVEGLRLRFAGKSIPTEKFAAKKAQRYSSSTPVKLVVPALEMMYVWNGFTIVGKKPELTEGILSTLEKAEEQLRNDPNPSEYHTGDECIVQLLKGLCLRSLGRLVQAEICFNHVISSDGGIKHDSYLVPFTMYELGLLHKQKGEINKAIAVMENVMTNYKDYNMESRLHFRIHAALNTMGSFAAKLPPSRTAA*-------------------

>AhR1.ENSMZET00005027059.1

MHQMEGEDNSLQQVGLKQPNEDAILPEELPKMALETALKECFTALNFFLNNRFADALALLKPWKSESMYHAMGYSSILVMQAAVTFDAKDMDAAMTSLREALQVCQRFRKK?GLMESLANLWYRQPADNLTEEEMHAELCYAEVLLQKAALTFLDESIIGFIKGGMKIRHSYQLYKNCQAMEKVTKDEEKQRSTHIHFRGGVSMGIGSFNLMLSLLPSRVLRLMEFLGFSGDREVGLSELREGATTNNLRSILSTLTLLMFQLYITVILGTADVNLSECEALLKPYCEKFPNGALMHFFSARIAVLKGNFTFAQEKFLACITSQEEWRQIHHLCYWELMWVYSFEQNWFEAYRYANLLSKENKWSQAVYVFQKAAILSMMPEEEVNKFNENVVELFRQVEGLRLRFAGKSIPTEKFAAKKAQRYSSSTPVKLVVPALEMMYVWNGFTIVGKKPELTEGILSTLEKAEEQLRNDPNPSEYHTGDECVVQLLKGLCLRSLGRLVQAEICFNHVISSDGGIKHDSYLVPFTMYELGLLHKQKGEINKAIAVMENVMTNYK?YNMESRLHFRIHAALNTMGSFAAKLPPSRTAA*-------------------

>AhR2.ENSMZET00005027059.1

MHQMEGEDNSLQQVGLKQPNEDAILPEELPKMALETALKECFTALNFFLNNRFADALALLKPWKSESMYHAMGYSSILVMQAAVTFDAKDMDAAMTSLREALQVCQRFRKK?GLMESLANLWYRQPADNLTEEEMHAELCYAEVLLQKAAL?FLDESIIGFIKGGMKIRHSYQLYKNCQAMEKVTKDEEKQRSTHIHFRGGVSMGIGSFNLMLSLLPSRVLRLMEFLGFSGDREVGLSELREGATTNNLRSILSTLTLLMFQLYITVILGTADVNLSECEALLKPYCEKFPNGALMHFFSARIAVLKGNFTFAQEKFLACITSQEEWRQIHHLCYWELMWVYSFEQNWFEAYRYANLLSKENKWSQAVYVFQKAAILSMMPEEEVNKFNENVVELFRQVEGLRLRFAGKSIPTEKFAAKKAQRYSSSTPVKLVVPALEMMYVWNGFTIVGKKPELTEGILSTLEKAEEQLRNDPNPSEYHTGDECVVQLLKGLCLRSLGRLVQAEICFNHVISSDGGIKHDSYLVPFTMYELGLLHKQKGEINKAIAVMENVMTNYK?YNMESRLHFRIHAALNTMGSFAAKLPPSRTAA*-------------------

>AhR3.ENSMZET00005027059.1

MHQMEGEDNSLQQVGLKQPNEDAILPEELPKMALETALKECFTALNFFLNNRFADALALLKPWKSESMYHAMGYSSILVMQAAVTFDAKDMDAAMTSLREALQVCQRFRKKTGLMESLANLWYRQPADNLTEEEMHAELCYAEVLLQKAALTFLDESIIGFIKGGMKIRHSYQLYKNCQAMEKVTKDEEKQRSTHIHFRGGVSMGIGSFNLMLSLLPSRVLRLMEFLGFSGDREVGLSELREGATTNNLRSILSTLTLLMFQLYITVILGTADVNLSECEALLKPYCEKFPNGALMHFFSARIAVLKGNFTFAQEKFLACITSQEEWRQIHHLCYWELMWVYSFEQNWFEAYRYANLLSKENKWSQAVYVFQKAAILSMMPEEEVNKFNENVVELFRQVEGLRLRFAGKSIPTEKFAAKKAQRYSSSTPVKLVVPALEMMYVWNGFTIVGKKPELTEGILSTLEKAEEQLRNDPNPSEYHTGDECVVQLLKGLCLRSLGRLVQAEICFNHVISSDGGIKHDSYLVPFTMYELGLLHKQKGEINKAIAVMENVMTNYKHYNMESRLHFRIHAALNTMGSFAAKLPPSRTAA*-------------------

>AhR4.ENSMZET00005027059.1

MHQMEGEDNSLQQVGLKQPNEDAILPEELPKMALETALKECFTAL?FFLNNRFADALALLKPWKSESMYHAMGYSSILVMQAAVTFDAKDMDAAMTSLREALQVCQRFRKK?GLMESLANLWYRQPADNLTEEEMHAELCYAEVLLQKAALTFLDESIIGFIKGGMKIRHSYQLYKNCQAMEKVTKDEEKQRSTHIHFRGGVSMGIGSFNLMLSLLPSRVLRLMEFLGFSGDREVGLSELREGATTNNLRSILSTLTLLMFQLYITVILGTADVNLSECEALLKPYCEKFPNGALMHFFSARIAVLKGNFTFAQEKFLACITSQEEWRQIHHLCYWELMWVYSFEQNWFEAYRYANLLSKENKWSQAVYVFQKAAILSMMPEEEVNKFNENVVELFRQVEGLRLRFAGKSIPTEKFAAKKAQRYSSSTPVKLVVPALEMMYVWNGFTIVGKKPELTEGILSTLEKAEEQLRNDPNPSEYHTGDECVVQLLKGLCLRSLGRLVQAEICFNHVISSDGGIKHDSYLVPFTMYELGLLHKQKGEINKAIAVMENVMTNYK?YNMESRLHFRIHAALNTMGSFAAKLPPSRTAA*-------------------

>AhR5.ENSMZET00005027059.1

MHQMEGEDNSLQQVGLKQPNEDAILPEELPKMALETALKECFTALNFFLNNRFADALALLKPWKSESMYHAMGYSSILVMQAAVTFDAKDMDAAMTSLREALQVCQRFRKKTGLMESLANLWYRQPADNLTEEEMHAELCYAEVLLQKAALTFLDESIIGFIKGGMKIRHSYQLYKNCQAMEKVTKDEEKQRSTHIHFRGGVSMGIGSFNLMLSLLPSRVLRLMEFLGFSGDREVGLSELREGATTNNLRSILSTLTLLMFQLYITVILGTADVNLSECEALLKPYCEKFPNGALMHFFSARIAVLKGNFTFAQEKFLACITSQEEWRQIHHLCYWELMWVYSFEQNWFEAYRYANLLSKENKWSQAVYVFQKAAILSMMPEEEVNKFNENVVELFRQVEGLRLRFAGKSIPTEKFAAKKAQRYSSSTPVKLVVPALEMMYVWNGFTIVGKKPELTEGILSTLEKAEEQLRNDPNPSEYHTGDECVVQLLKGLCLRSLGRLVQAEICFNHVISSDGGIKHDSYLVPFTMYELGLLHKQKGEINKAIAVMENVMTNYKHYNMESRLHFRIHAALNTMGSFAAKLPPSRTAA*-------------------

>AhR6.ENSMZET00005027059.1

MHQMEGEDNSLQQVGLKQPNEDAILPEELPKMALETALKECFTALNFFLNNRFADALALLKPWKSESMYHAMGYSSILVMQAAVTFDAKDMDAAMTSLREALQVCQRFRKK?GLMESLANLWYRQPADNLTEEEMHAELCYAEVLLQKAAL?FLDESIIGFIKGGMKIRHSYQLYKNCQAMEKVTKDEEKQRSTHIHFRGGVSMGIGSFNLMLSLLPSRVLRLMEFLGFSGDREVGLSELREGATTNNLRSILSTLTLLMFQLYITVILGTADVNLSECEALLKPYCEKFPNGALMHFFSARIAVLKGNFTFAQEKFLACI?SQEEWRQIHHLCYWELMWVYSFEQNWFEAYRYANLLSKENKWSQAVYVFQKAAILSMMPEEEVNKFNENVVELFRQVEGLRLRFAGKSIPTEKFAAKKAQRYSSSTPVKLVVPALEMMYVWNGFTIVGKKPELTEGILSTLEKAEEQLRNDPNPSEYHTGDECVVQLLKGLCLRSLGRLVQAEICFNHVISSDGGIKHDSYLVPFTMYELGLLHKQKGEINKAIAVMENVMTNYKDYNMESRLHFRIHAALNTMGSFAAKLPPS?TAA*-------------------

>AbY1.ENSMZET00005027059.1

MHQMEGEDNSLQQVGLKQPNEDAILPEELPKMALETALKECFTALNFFLNNRFADALALLKPWKSESMYHAMGYSSILVMQAAVTFDAKDMDAAMTSLREALQVCQRFRKKTGLMESLANLWYRQPADNLTEEEMHAELCYAEVLLQKAALTFLDESIIGFIKGGMKIRHSYQLYKNCQAMEKVTKDEEKQRSTHIHFRGGVSMGIGSFNLMLSLLPSRVLRLMEFLGFSGDREVGLSELREGATTNNLRSILSTLTLLMFQLYITVILGTADVNLSECEALLKPYCEKFPNGALMHFFSARIAVLKGNFTFAQEKFLACIASQEEWRQIHHLCYWELMWVYSFEQNWFEAYRYANLLSKENKWSQAVYVFQKAAILSMMPEEEVNKFNENVVELFRQVEGLRLRFAGKSIPTEKFAAKKAQRYSSSTPVKLVVPALEMMYVWNGFTIVGKKP?LTEGILSTLEKAEEQLRNDPNPSEYHTGDECVVQLLKGLCLRSLGRLVQAEICFNHVISSDGGIKHDSYLVPFTMYELGLLHKQKGEINKAIAVMENVMTNYKDYNMESRLHFRIHAALNTMGSFAAKLPPSCTAA*-------------------

>AbY2.ENSMZET00005027059.1

MHQMEGEDNSLQQVGLKQPNEDAILPEELPKMALETALKECFTALNFFLNNRFADALALLKPWKSESMYHAMGYSSILVMQAAVTFDAKDMDAAMTSLREALQVCQRFRKKTGLMESLANLWYRQPADNLTEEEMHAELCYAEVLLQKAALTFLDESIIGFIKGGMKIRHSYQLYKNCQAMEKVTKDEEKQRSTHIHFRGGVSMGIGSFNL?LSLLPSRVLRLMEFLGFSGDREVGLSELREGATTNNLRSILSTLTLLMFQLYITVILGTADVNLSECEALLKPYCEKFPNGALMHFFSARIAVLKGNFTFAQEKFLACIASQEEWRQIHHLCYWELMWVYSFEQNWFEAYRYANLLSKENKWSQAVYVFQKAAILSMMPEEEVNKFNENVVELFRQVEGLRLRFAGKSIPTEKFAAKKAQRYSSSTPVKLVVPALEMMYVWNGFTIVGKKP?LTEGILSTLEKAEEQLRNDPNPSEYHTGDECVVQLLKGLCLRSLGRLVQAEICFNHVISSDGGIKHDSYLVPFTMYELGLLHKQKGEINKAIAVMENVMTNYKDYNMESRLHFRIHAALNTMGSFAAKLPPSCTAA*-------------------

>AbY5.ENSMZET00005027059.1

MHQMEGEDNSLQQVGLKQPNEDAILPEELPKMALETALKECFTALNFFLNNRFADALALLKPWKSESMYHAMGYSSILVMQAAVTFDAKDMDAAMTSLREALQVCQRFRKKTGLMESLANLWYRQPADNLTEEEMHAELCYAEVLLQKAALTFLDESIIGFIKGGMKIRHSYQLYKNCQAMEKVTKDEEKQRSTHIHFRGGVSMGIGSFNLMLSLLPSRVLRLMEFLGFSGDREVGLSELREGATTNNLRSILSTLTLLMFQLYITVILGTADVNLSECEALLKPYCEKFPNGALMHFFSARIAVLKGNFTFAQEKFLACIASQEEWRQIHHLCYWELMWVYSFEQNWFEAYRYANLLSKENKWSQAVYVFQKAAILSMMPEEEVNKFNENVVELFRQVEGLRLRFAGKSIPTEKFAAKKAQRYSSSTPVKLVVPALEMMYVWNGFTIVGKKPKLTEGILSTLEKAEEQLRNDPNPSEYHTGDECVVQLLKGLCLRSLGRLVQAEICFNHVISSDGGIKHDSYLVPFTMYELGLLHKQKGEINKAIAVMENVMTNYKDYNMESRLHFRIHAALNTMGSFAAKLPPSCTAA*-------------------

>AbY3.ENSMZET00005027059.1

MHQMEGEDNSLQQVGLKQPNEDAILPEELPKMALETALKECFTALNFFLNNRFADALALLKPWKSESMYHAMGYSSILVMQAAVTFDAKDMDAAMTSLREALQVCQRFRKKTGLMESLANLWYRQPADNLTEEEMHAELCYAEVLLQKAALTFLDESIIGFIKGGMKIRHSYQLYKNCQAMEKVTKDEEKQRSTHIHFRGGVSMGIGSFNL?LSLLPSRVLRLMEFLGFSGDREVGLSELREGATTNNLRSILSTLTLLMFQLYITVILGTADVNLSECEALLKPYCEKFPNGALMHFFSARIAVLKGNFTFAQEKFLACIASQEEWRQIHHLCYWELMWVYSFEQNWFEAYRYANLLSKENKWSQAVYVFQKAAILSMMPEEEVNKFNENVVELFRQVEGLRLRFAGKSIPTEKFAAKKAQRYSSSTPVKLVVPALEMMYVWNGFTIVGKKP?LTEGILSTLEKAEEQLRNDPNPSEYHTGDECVVQLLKGLCLRSLGRLVQAEICFNHVISSDGGIKHDSYLVPFTMYELGLLHKQKGEINKAIAVMENVMTNYKDYNMESRLHFRIHAALNTMGSFAAKLPPSCTAA*-------------------

>AbY4.ENSMZET00005027059.1

MHQMEGEDNSLQQVGLKQPNEDAILPEELPKMALETALKECFTALNFFLNNRFADALALLKPWKSESMYHAMGYSSILVMQAAVTFDAKDMDAAMTSLREALQVCQRFRKKTGLMESLANLWYRQPADNLTEEEMHAELCYAEVLLQKAALTFLDESIIGFIKGGMKIRHSYQLYKNCQAMEKVTKDEEKQRSTHIHFRGGVSMGIGSFNLMLSLLPSRVLRLMEFLGFSGDREVGLSELREGATTNNLRSILSTLTLLMFQLYITVILGTADVNLSECEALLKPYCEKFPNGALMHFFSARIAVLKGNFTFAQEKFLACIASQEEWRQIHHLCYWELMWVYSFEQNWFEAYRYANLLSKENKWSQAVYVFQKAAILSMMPEEEVNKFNENVVELFRQVEGLRLRFAGKSIPTEKFAAKKAQRYSSSTPVKLVVPALEMMYVWNGFTIVGKKPKLTEGILSTLEKAEEQLRNDPNPSEYHTGDECVVQLLKGLCLRSLGRLVQAEICFNHVISSDGGIKHDSYLVPFTMYELGLLHKQKGEINKAIAVMENVMTNYKDYNMESRLHFRIHAALNTMGSFAAKLPPSCTAA*-------------------

>AbY6.ENSMZET00005027059.1

MHQMEGEDNSLQQVGLKQPNEDAILPEELPKMALETALKECFTALNFFLNNRFADALALLKPWKSESMYHAMGYSSILVMQAAVTFDAKDMDAAMTSLREALQVCQRFRKKTGLMESLANLWYRQPADNLTEEEMHAELCYAEVLLQKAALTFLDESIIGFIKGGMKIRHSYQLYKNCQAMEKVTKDEEKQRSTHIHFRGGVSMGIGSFNL?LSLLPSRVLRLMEFLGFSGDREVGLSELREGATTNNLRSILSTLTLLMFQLYITVILGTADVNLSECEALLKPYCEKFPNGALMHFFSARIAVLKGNFTFAQEKFLACIASQEEWRQIHHLCYWELMWVYSFEQNWFEAYRYANLLSKENKWSQAVYVFQKAAILSMMPEEEVNKFNENVVELFRQVEGLRLRFAGKSIPTEKFAAKKAQRYSSSTPVKLVVPALEMMYVWNGFTIVGKKP?LTEGILSTLEKAEEQLRNDPNPSEYHTGDECVVQLLKGLCLRSLGRLVQAEICFNHVISSDGGIKHDSYLVPFTMYELGLLHKQKGEINKAIAVMENVMTNYKDYNMESRLHFRIHAALNTMGSFAAKLPPSCTAA*-------------------

>TbR1.TM1 T0000032630-R1

---MEGEDNSLQQVGLKQPNEDAILPEELPKMGLETALKECFTALNFFLNNRFADALALLKPWKSESMYHAMGYSSILVMQAAMTFDAKDMDAAMTSLREALQVCQRFRKKTGLMESLANLWYRQPADNLTEEEMHAELCYAEVLLQKAALTFLDESIIGFIKGGMKIRHSYQLYKDCQAMEKVTKDEEKQRSTHIHFRGGVSMGIGSFNLMLSLLPSRVLRLMEFLGFSGDREVGLSELREGATTNNLRSILSTLTLLMFQLYITVILGTADVNLSECEALLKPYCEKFPNGALMHFFSARIAVLKGNFTFAQEKFLACIASQEEWRQIHHLCYWELMWVYSFEQNWFEAYRYANLLSKENKWSQAVYVFQKAAILSMMPEEEVNKLNENVVDLFRQVEGLRLRFAGKSIPTEKFAAKKAQRYSSSTPVKLVVPALEMMYVWNGFTIVGKRPELTEGILSTLEKAEEQLRNDPNPSEYHTGDECIVQLLKGLCLRSLGRLVQAEICFNYVISSDGGIKHDSYLVPFTMYELGLLHKQKGEINKAIAVMENVMTNYKDYNMESRLHFRIHAALNTIGSFAAKLPPSRTAA*EIPLHET*QCLL?------

>TbR2.TM1 T0000032630-R1

---MEGEDNSLQQVGLKQPNEDAILPEELPKMGLETALKECFTALNFFLNNRFADALALLKPWKSESMYHAMGYSSILVMQAAMTFDAKDMDAAMTSLREALQVCQRFRKKTGLMESLANLWYRQPADNLTEEEMHAELCYAEVLLQKAALTFLDESIIGFIKGGMKIRHSYQLYKDCQAMEKVTKDEEKQSSTHIHFRGGVSMGIGSFNLMLSLLPSRVLRLMEFLGFSGDREVGLSELREGATTNNLRSILST?TLLMFQLYITVILGTADVNLSECEALLKPYCEKFPNGALMHFFSARIAVLKGNFTFAQEKFLACIASQEEWRQIHHLCYWELMWVYSFEQNWFEAYRYANLLSKENKWSQAVYVFQKAAILSMMPEEEVNKLNENVVDLFRQVEGLRLRFAGKSIPTEKFAAKKAQRYSSSTPVKLVVPALEMMYVWNGFTIVGKRPELTEGILSTLEKAEEQLRNDPNPSEYHTGDECIVQLLKGLCLRSLGRLVQAEICFNYVISSDGGIKHDSYLVPFTMYELGLLHKQKGEINKAIAVMENVMTNYKDYNMESRLHFRIHAALNTIGSFAAKLPPSRTAA*EIPLHET*QCLLFH?----

>TbR3.TM1 T0000032630-R1

---MEGEDNSLQQVGLKQPNEDAILPEELPKMGLETALKECFTALNFFLNNRFADALALLKPWKSESMYHAMGYSSILVMQAAMTFDAKDMDAAMTSLREALQVCQRFRKKTGLMESLANLWYRQPADNLTEEEMHAELCYAEVLLQKAALTFLDESIIGFIKGGMKIRHSYQLYKDCQAMEKVTKDEEKQ?S?HIHFRGGVSMGIGSFNLMLSLLPSRVLRLMEFLGFSGDREVGLSELREGATTNNLRSILSTLTLLMFQLYITVILGTADVNLSECEALLKPYCEKFPNGALMHFFSARIAVLKGNFTFAQEKFLACIASQEEWRQIHHLCYWELMWVYSFEQNWFEAYRYANLLSKENKWSQAVYVFQKAAILSMMPEEEVNKLNENVVDLFRQVEGLRLRFAGKSIPTEKFAAKKAQRYSSSTPVKLVVPALEMMYVWNGFTIVGKRPELTEGILSTLEKAEEQLRNDPNPSEYHTGDECIVQLLKGLCLRSLGRLVQAEICFNYVISSDGGIKHDSYLVPFTMYELGLLHKQKGEINKAIAVMENVMTNYKDYNMESRLHFRIHAALNTIGSFAAKLPPSRTAA*EIPLHET*QCLL?------

>TbR4.TM1 T0000032630-R1

---MEGEDNSLQQVGLKQPNEDAILPEELPKMGLETALKECFTALNFFLNNRFADALALLKPWKSESMYHAMGYSSILVMQAAMTFDAKDMDAAMTSLREALQVCQRFRKKTGLMESLANLWYRQPADNLTEEEMHAE?CYAEVLLQKAAL?FLDESIIGFIKGGMKIRHSYQLYKDCQAMEKVTKDEEKQSSTHIHFRGGVSMGIGSFNLMLSLLPSRVLRLMEFLGFSGDREVGLSELREGATTNNLRSILSTLTLLMFQLYITVILGTADVNLSECEALLKPYCEKFPNGALMHFFSARIAVLKGNFTFAQEKFLACIASQEEWRQIHHLCYWELMWVYSFEQNWFEAYRYANLLSKENKWSQAVYVFQKAAILSMMPEEEVNKLNENVVDLFRQVEGLRLRFAGKSIPTEKFAAKKAQRYSSSTPVKLVVPALEMMYVWNGFTIVGKRPELTEGILSTLEKAEEQLRNDPNPSEYHTGDECIVQLLKGLCLRSLGRLVQAEICFNYVISSDGGIKHDSYLVPFTMYELGLLHKQKGEINKAIAVMENVMTNYKDYNMESRLHFRIHAALNTIGSFAAKLPPSRTAA*EIPLHET*QCLLFH?----

>TbR5.TM1 T0000032630-R1

---MEGEDNSLQQVGLKQPNEDAILPEELPKMGLETALKECFTALNFFLNNRFADALALLKPWKSESMYHAMGYSSILVMQAAMTFDAKDMDAAMTSLREALQVCQRFRKKTGLMESLANLWYRQPADNLTEEEMHAELCYAEVLLQKAALTFLDESIIGFIKGGMKIRHSYQLYKDCQAMEKVTKDEEKQSSTHIHFRGGVSMGIGSFNLMLSLLPSRVLRLMEFLGFSGDREVGLSELREGATTNNLRSILSTLTLLMFQLYITVILGTADVNLSECEALLKPYCEKFPNGALMHFFSARIAVLKGNFTFAQEKFLACIASQEEWRQIHHLCYWELMWVYSFEQNWFEAYRYANLLSKENKWSQAVYVFQKAAILSMMPEEEVNKLNENVVDLFRQVEGLRLRFAGKSIPTEKFAAKKAQRYSSSTPVKLVVPALEMMYVWNGFTIVGKRPELTEGILSTLEKAEEQLRNDPNPSEYHTGDECIVQLLKGLCLRSLGRLVQAEICFNYVISSDGGIKHDSYLVPFTMYELGLLHKQKGEINKAIAVMENVMTNYKDYNMESRLHFRIHAALNTIGSFAAKLPPSRTAA*EIPLHET*QCLL?------

>TbR6.TM1 T0000032630-R1

---MEGEDNSLQQVGLKQPNEDAILPEELPKMGLETALKECFTALNFFLNNRFADALALLKPWKSESMYHAMGYSSILVMQAAMTFDAKDMDAAMTSLREALQVCQRFRKKTGLMESLANLWYRQPADNLTEEEMHAELCYAEVLLQKAALTFLDESIIGFIKGGMKIRHSYQLYKDCQAMEKVTKDEEKQRSTHIHFRGGVSMGIGSFNLMLSLLPSRVLRLMEFLGFSGDREVGLSELREGATTNNLRSILSTLTLLMFQLYITVILGTADVNLSECEALLKPYCEKFPNGALMHFFSARIAVLKGNFTFAQEKFLACIASQEEWRQIHHLCYWELMWVYSFEQNWFEAYRYANLLSKENKWSQAVYVFQKAAILSMMPEEEVNKLNENVVDLFRQVEGLRLRFAGKSIPTEKFAAKKAQRYSSSTPVKLVVPALEMMYVWNGFTIVGKRPELTEGILSTLEKAEEQLRNDPNPSEYHTGDECIVQLLKGLCLRSLGRLVQAEICFNYVISSDGGIKHDSYLVPFTMYELGLLHKQKGEINKAIAVMENVMTNYKDYNMESRLHFRIHAALNTIGSFAAKLPPSRTAA*EIPLHET*QCLL?------

>TbY1.TM1 T0000032630-R1

---???????????????????TILPEELPKMGLETALKECFTALNFFLNNRFADALALLKPWKSESMYHAMGYSSILVMQAAMTFDAKDMDAAMTSLREALQVCQ????KTGLMESLANLWYRQPADNLTEEEMHAELCYAEVLLQKAALTFLDESIIGFIKGGMKIRHSYQLYKDC???????????????????????VSMGIGSFNLMLSLLPSRVLRLMEFLGFSGDREVGLSELREGATTNNLRSILSTLTLLMFQLYITVILGTADVNLSECEALLKPYC????????????????????GNFTFAQEKFLACITSQEEWRQIHHLCYWELMWVYSFEQNWFEAYRYANLLSKENKWSQAVYVFQKAAILSMMPEEEVNKLNENVVDLFRQVEGLRLRFAGKSIPTEKFAAKKAQRYSSSTPVKLVVPALEMMYVWNGFTIVGKRPELTEGILSTLEKAEEQLRNDPNPSEYHTGDECVVQLLKGLCLRSLGRLVQAEICFNYVISSDGGIKHDSYLVPFTMYELGLLHKQKGEINKAIAVMENVMTNYKDYNMESRLHFRIHAALNTMGSFAAKLPPSRTAA*EIPLHET*HCLL?------

>TbY2.TM1 T0000032630-R1

---MEGEDNSLQQVGLKQPNEDAILPEELPKMGLETALKECFTALNFFLNNRFADALALLKPWKSESMYHAMGYSSILVMQAAMTFDAKDMDAAMTSLREALQVCQRFRKKTGLMESLANLWYRQPADNLTEEEMHAELCYAEVLLQKAALTFLDESIIGFIKGGMKIRHSYQLYKDCQAMEKVTKDEEKQRSAHIHFRGGVSMGIGSFNLMLSLLPSRVLRLMEFLGFSGDREVGLSELREGATTNNLRSILSTLTLLMFQLYITVILGTADVNLSECEALLKPYCEKFPNGALMHFFSARIAVLKGNFTFAQEKFLACIASQEEWRQIHHLCYWELMWVYSFEQNWFEAYRYANLLSKENKWSQAVYVFQKAAILSMMPEEEVNKLNENVVDLFRQVEGLRLRFAGKSIPTEKFAAKKAQRYSSSTPVKLVVPALEMMYVWNGFTIVGKRPELTEGILSTLEKAEEQLRNDPNPSEYHTGDECIVQLLKGLCLRSLGRLVQAEICFNYVISSDGGIKHDSYLVPFTMYELGLLHKQKGEINKAIAVMENVMTNYKDYNMESRLHFRIHAALNTIGSFAAKLPPSRTAA*EIPLHET*QC?--------

>TbY3.TM1 T0000032630-R1

---?EGEDNSLQQVGLKQPNEDAILPEELPKMGLETALKE?FTALNFFLNNRFADALALLKPWKSESMYHAMGYSSILVMQAAMTFDAKDMDAAMTSLREALQVCQRFRKKTGLMESLANLWYRQPADNLTEEEMHAELCYAEVLLQKAALTFLDESIIGFIKGGMKIRHSYQLYKDCQAMEKVTKDEEKQRSTHIHFRGGVSMGIGSFNLMLSLLPSRVLRLMEFLGFSGDREVGLSELREGATTNNLRSILSTLTLLMFQLYITVILGTADVNLSECEALLKPYCEKFPNGALMHFFSARIAVLKGNFTFAQEKFLACIASQEEWRQIHHLCYWELMWVYSFEQNWFEAYRYANLLSKENKWSQAVYVFQKAAILSMMPEEEVNKLNENVVDLFRQVEGLRLRFAGKSIPTEKFAAKKAQRYSSSTPVKLVVPALEMMYVWNGFTIVGKRPELTEGILSTLEKAEEQLRNDPNPSEYHTGDEC?VQLLKGLCLRSLGRLVQAEICFN??ISSDGGIKHDSYLVPFTMYELGLLHKQKGEINKAIAVMENVMTNYKDYNMESRLHFRIHAALNT?GSFAAKLPPSRTAA*EIPLHET*QC---------

>TbY4.TM1 T0000032630-R1

---????DNSLQQVGLKQPNEDAILPEELPKMGLETALKECFTALNFFLNNRFADALALLKPWKSES???????SSILVMQAAMTFDAKDMDAAMTSLREALQVCQRFRKKTGLMESLANLWYRQPADNLTEEEMHAELCYAEVLLQKAALTFLDESIIGFIKGGMKIRHSYQLYKDCQAMEKVTKDEEKQRSAHIHFRGGVSMGIGSFNLMLSLLPSRVLRLMEFLGFSGDREVGLSELREGATTNNLRSILSTLTLLMFQLYITVILGTADVNLSECEALLKPYCEKFPNGALMHFFSARIAVLKGNFTFAQEKFLACIASQEEWRQIHHLCYWELMWVYSFEQNWFEAYRYANLLSKENKWSQAVYVFQKAAILSMMPEEEVNKLNENVVDLFRQVEGLRLRFAGKSIPTEKFAAKKAQRYSSSTPVKLVVPALEMMYVWNGFTIVGKRPELTEGILSTLEKAEEQLRNDPNPSEYHTGDECVVQLLKGLCLRSLGRLVQAEICFNYVISSDGGIKHDSYLVPFTMYELGLLHKQKGEINKAIAVMENVMTNYKDYNMESRLHFRIHAALNTMGSFAAKLPPSRTAA*EIPLHE?------------

>TbY5.TM1 T0000032630-R1

---????????????LKQPNEDAILPEELPKMGLETALKECFTALNFFLNNRFADALALLKPWKSESMYHAMGYSSILVMQAAMTFDAKDMDAAMTSLREALQVCQRFRKKTGLMESLANLWYRQPADNLTEEEMHAELCYAEVLLQKAA???????????????????????????????????????????????????????????????????SRVLRLMEFLGFSGDREVGLSELREGATTNNLRSILSTLTLLMFQLYITVILGTADVNLSE??????????????????????????????????AQEKFLACIASQEEWRQIHHLCYWELMWVYSFEQNWFEAYRYANLLSKENKWSQAVYVFQKAAILSMMPEEEVNKLNENVVDLFRQVEGLRLRFAGKSIPTEKFAAKKAQRYSSSTPVKLVVPALEMMYVWNGFTIVGKRPELTEGILSTLEKAEEQLRNDPNPSEYHTGDECVVQLLKGLCLRSLGRLVQAEICFNYVISSDGGIKHDSYLVPFTMYELGLLHKQKGEINKAIAVMENVMTNYKDYNMESRLHFRIHAALNTMGSFAAKLPPTRTAV*EIP?---------------

>TbY6.TM1 T0000032630-R1

---??????????????????????????????????????????????????????????????ESMYHAMGYSSILVMQAAMTFDAKDMDAAMTSLREALQVCQRFRKKTGLMESLANLWYRQPADNLTEEEMHAELCYAEVLLQKAALTFLDESIIGFIKGGMKIRHSYQLYKDCQAMEKVTKDEEKQRSAHIHFRGG?????????????????????????????GDREVGLSELREGATTNNLRSILSTLTLLMFQLYITVILGTADVNLSECEALLKPYCEKFPNGALMHFFSARIAVLKGNFTFAQEKFLACI?SQEEWRQIHHLCYWELMWVYSFEQNWFEAYRYAN?LSKENKWSQAVYVFQKAAILSMMPEEEVNKLNENVVDLF???????LRFAGKSIPTEKFAAKKAQRYSSSTPVKLVVPALEMMYVWN?????GKRPELTEGILSTLEKAEEQLRNDPNPSEYHTGDECVVQLLKGLCLRSLGRLVQAEICFNYVISSDGGIKHDSYLVPFTMYELGLLHKQKGEINKAIAVMENVMTNYKDYNMESRLHFRIHAALNTMGSFAAKLPP?------------------------

>TmR1.TM1 T0000032630-R1

---MEGEDNSLQQVGLKQPNEDTILPEELPKMGLETTLKECFTALNFFLNNRFADALALLKPWKSESMYHAMGYSSILVMQAAMTFDAKDMDAAMTSLREALQVCQRFRKKTGLMESLANLWYRQPADNLTEEEMHAELCYAEVLLQKAALTFLDESIIGFIKGGMKIRHSYQLYKDCQAMEKVTKDEEKQRSTHIHFRGGVSMGIGSFNLMLSLLPSRVLRLMEFLGFSGDREVGLSELREGATTNNLRSILSTLTLLMFQLYITVILGTADVNLSECEALLKPYCEKFPNGALMHFFSARIAVLKGNFTFAQEKFLACIALQEEWRQIHHLCYWELMWVYSFEQNWFEAYRYANLLSKENKWSQAVYVFQKAAILSMMPEEEVNKLNENVVELFRQVEGLRLRFAGKSIPTEKFAAKKAQRYSSSTPVKLVVPALEMMYVWNGFTIVGKRPELTEGILSTLEKAEEQLRNDPNPSEYHTGDECVVQLLKGLCLRSLGQLVQAEICFNYVISSDGGIKHDSYLVPFTMYELGLLHKQKGEINKAIAVMENVMTNYKDYNMESRLHFRIHAALNTMGSFAAKLPPTRTAV*EIPLHET*QCLLFHCLLPS

>TmR2.TM1 T0000032630-R1

---MEGEDNSLQQVGLKQPNEDTILPEELPKMGLETALKECFTALNFFLNNRFADALALLKPWKSESMYHAMGYSSILVMQAAMTFDAKDMDAAMTSLREALQVCQRFRKKTGLMESLANLWYRQPADNLTEEEMHAELCYAEVLLQKAALTFLDESIIGFIKGGMKIRHSYQLYKDCQAMEKVTKDEEKQRSTHIHFRGGVSMGIGSFNLMLSLLPSRVLRLMEFLGFSGDREVGLSELREGA?TNNLRSILSTLT?LMFQLYITVILGTADVNLSECEALLKPYCEKFPNGALMHFFSARIAVLKGNFTFAQEKFLACIALQEEWRQIHHLCYWELMWVYSFEQNWFEAYRYANLLSKENKWSQAVYVFQKAAILSMMPEEEVNKLNENVVELFRQVEGLRLRFAGKSIPTEKFAAKKAQRYSSSTPVKLVVPALEMMYVWNGFTIVGKRPELTEGILSTLEKAEEQLRNDPNPSEYHTGDECVVQLLKGLCLRSLGQLVQAEICFNYVISSDGGIKHDSYLVPFTMYELGLLHKQKGEINKAIAVMENVMTNYKDYNMESRLHFRIHAALNTMGSFAAKLPPTR?AV*EIPLHET*QCLLFHCLLP?

>TmR3.TM1 T0000032630-R1

---MEGEDNSLQQVGLKQPNEDTILPEELPKMGLETALKECFTALNFFLNNRFADALALLKPWKSESMYHAMGYSSILVMQAAMTFDAKDMDAAMTSLREALQVCQRFRKKTGLMESLANLWYRQPADNLTEEEMHAELCYAEVLLQKAAL?FLDESIIGFIKGGMKIRHSYQLYKDCQAMEKVTKDEEKQRSTHIHFRGGVSMGIGSFNLMLSLLPSRVLRLMEFLGFSGDREVGLSELREGATTNNLRSILSTLT?LMFQLYITVILGTADVNLSECEALLKPYCEKFPNGALMHFFSARIAVLKGNFTFAQEKFLACIALQEEWRQIHHLCYWELMWVYSFEQNWFEAYRYANLLSKENKWSQAVYVFQKAAILSMMPEEEVNKLNENVVELFRQVEGLRLR?AGKSIPTEKFAAKKAQRYSSSTPVKLVVPALEMMYVWNGFTIVGKRPELTEGILSTLEKAEEQLRNDPNPSEYHTGDECVVQLLKGLCLRSLGQLVQAEICFNYVISSDGGIKHDSYLVPFTMYELGLLHKQKGEINKAIAVMENVMTNYKDYNMESRLHFRIHAALNTMGSFAAKLPPTRTAV*EIPLHET*QCLLFHCLLPS

>TmR4.TM1 T0000032630-R1

---MEGEDNSLQQVGLKQPNEDTILPEELPKMGLETTLKECFTALNFFLNNRFADALALLKPWKSESMYHAMGYSSILVMQAAMTFDAKDMDAAMTSLREALQVCQRFRKKTGLMESLANLWYRQPADNLTEEEMHAELCYAEVLLQKAALTFLDESIIGFIKGGMKIRHSYQLYKDCQAMEKVTKDEEKQRSTHIHFRGGVSMGIGSFNLMLSLLPSRVLRLMEFLGFSGDREVGLSELREGATTNNLRSILSTLTLLMFQLYITVILGTADVNLSECEALLKPYCEKFPNGALMHFFSARIAVLKGNFTFAQEKFLACIALQEEWRQIHHLCYWELMWVYSFEQNWFEAYRYANLLSKENKWSQAVYVFQKAAILSMMPEEEVNKLNENVVELFRQVEGLRLRFAGKSIPTEKFAAKKAQRYSSSTPVKLVVPALEMMYVWNGFTIVGKRPELTEGILSTLEKAEEQLRNDPNPSEYHTGDECVVQLLKGLCLRSLGQLVQAEICFNYVISSDGGIKHDSYLVPFTMYELGLLHKQKGEINKAIAVMENVMTNYKDYNMESRLHFRIHAALNTMGSFAAKLPPTRTAV*EIPLHET*QCLLFHCLLPS

>TmR5.TM1 T0000032630-R1

---MEGEDNSLQQVGLKQPNEDTILPEELPKMGLETTLKECFTALNFFLNNRFADALALLKPWKSESMYHAMGYSSILVMQAAMTFDAKDMDAAMTSLREALQVCQRFRKKTGLMESLANLWYRQPADNLTEEEMHAELCYAEVLLQKAALTFLDESIIGFIKGGMKIRHSYQLYKDCQAMEKVTKDEEKQRSTHIHFRGGVSMGIGSFNLMLSLLPSRVLRLMEFLGFSGDREVGLSELREGATTNNLRSILSTLTLLMFQLYITVILGTADVNLSECEALLKPYCEKFPNGALMHFFSARIAVLKGNFTFAQEKFLACIALQEEWRQIHHLCYWELMWVYSFEQNWFEAYRYANLLSKENKWSQAVYVFQKAAILSMMPEEEVNKLNENVVELFRQVEGLRLRFAGKSIPTEKFAAKKAQRYSSSTPVKLVVPALEMMYVWNGFTIVGKRPELTEGILSTLEKAEEQLRNDPNPSEYHTGDECVVQLLKGLCLRSLGQLVQAEICFNYVISSDGGIKHDSYLVPFTMYELGLLHKQKGEINKAIAVMENVMTNYKDYNMESRLHFRIHAALNTMGSFAAKLPPTRTAV*EIPLHET*QCLLFHCLLPS

>TmR6.TM1 T0000032630-R1

---MEGEDNSLQQVGLKQPNEDTILPEELPKMGLETTLKECFTALNFFLNNRFADALALLKPWKSESMYHAMGYSSILVMQAAMTFDAKDMDAAMTSLREALQVCQRFRKKTGLMESLANLWYRQPADNLTEEEMHAELCYAEVLLQKAALTFLDESIIGFIKGGMKIRHSYQLYKDCQAMEKVTKDEEKQRSTHIHFRGGVSMGIGSFNLMLSLLPSRVLRLMEFLGFSGDREVGLSELREGATTNNLRSILSTLTLLMFQLYITVILGTADVNLSECEALLKPYCEKFPNGALMHFFSARIAVLKGNFTFAQEKFLACIALQEEWRQIHHLCYWELMWVYSFEQNWFEAYRYANLLSKENKWSQAVYVFQKAAILSMMPEEEVNKLNENVVELFRQVEGLRLRFAGKSIPTEKFAAKKAQRYSSSTPVKLVVPALEMMYVWNGFTIVGKRPELTEGILSTLEKAEEQLRNDPNPSEYHTGDECVVQLLKGLCLRSLGQLVQAEICFNYVISSDGGIKHDSYLVPFTMYELGLLHKQKGEINKAIAVMENVMTNYKDYNMESRLHFRIHAALNTMGSFAAKLPPTRTAV*EIPLHET*QCLLFHCLLPS

>TmY1.TM1 T0000032630-R1

---MEGEDNSLQQVGLKQPNEDTILPEELPKMGLETALKECFTALNFFLNNRFADALALLKPWKSESMYHAMGYSSILVMQAAMTFDAKDMDAAMTSLREALQVCQRFRKKTGLMESLANLWYRQPADNLTEEEMHAELCYAEVLLQKAALTFLDESIIGFIKGGMKIRHSYQLYKDCQAMEKVTKDEEKQRS?HIHFRGGVSMGIGSFNLMLSLLPSRVLRLMEFLGFSGDREVGLSELREGATTNNLRSILSTLTLLMFQLYITVILGTADVNLSECEALLKPYCEKFPNGALMHFFSARIAVLKGNFTFAQEKFLA?IA?QEEWRQIHHLCYWELMWVYSFEQNWFEAYRYANLLSKENKWSQAVYVFQKAAILSMMPEEEVNKLNENVVELFRQVEGLRLRFAGKSIPTEKFAAKKAQRYSSST?VKLVVPALEMMYVWNGFTIVGKRPELTEGILSTLEKAEEQLRNDPNPS?YHTGDECVVQLLKGLCLRSLGRLVQAEICFNYVISSDGGIKHDSYLVPFTMYELGLLHKQKGEINKAIAVMENVMTNYKDYNMESRLHFRIHAALNTMGSFAAKLPPTRTAV*EIPLHET*QCLLFHCLLP?

>TmY2.TM1 T0000032630-R1

---MEGEDNSLQQVGLKQPNEDTILPEELPKMGLETALKECFTALNFFLNNRFADALALLKPWKSESMYHAMGYSSILVMQAAMTFDAKDMDAAMTSLREALQVCQRFRKKTGLMESLANLWYRQPADNLTEEEMHAELCYAEVLLQKAALTFLDESIIGFIKGGMKIRHSYQLYKDCQAMEKVTKDEEKQRSAHIHFRGGVSMGIGSFNLMLSLLPSRVLRLMEFLGFSGDREVGLSELREGATTNNLRSILSTLTLLMFQLYITVILGTADVNLSECEALLKPYCEKFPNGALMHFFSARIAVLKGNFTFAQEKFLA?IA?QEEWRQIHHLCYWELMWVYSFEQNWFEAYRYANLLSKENKWSQAVYVFQKAAILSMMPEEEVNKLNENVVELFRQVEGLRLRFAGKSIPTEKFAAKKAQRYSSST?VKLVVPALEMMYVWNGFTIVGKRPELTEGILSTLEKAEEQLRNDPNPS?YHTGDECVVQLLKGLCLRSLGRLVQAEICFNYVISSDGGIKHDSYLVPFTMYELGLLHKQKGEINKAIAVMENVMTNYKDYNMESRLHFRIHAALNTMGSFAAKLPPTRTAV*EIPLHET*QCLLFHCLLP?

>TmY3.TM1 T0000032630-R1

---?EGEDNSLQQVGLKQPNEDTILPEELPKMGLETALKECFTALNFFLNNRFADALALLKPWKSESMYHAMGYSSILVMQAAMTFDAKDMDAAMTSLREALQVCQRFRKKTGLMESLANLWYRQPADNLTEEEMHAELCYAEVLLQKAALTFLDESIIGFIKGGMKIRHSYQLYKDCQAMEKVTKDEEKQRS?HIHFRGGVSMGIGSFNLMLSLLPSRVLRLMEFLGFSGDREVGLSELREGATTNNLRSILSTLTLLMFQLYITVILGTADVNLSECEALLKPYCEKFPNGALMHFFSARIAVLKGNFTFAQEKFLA?IA?QEEWRQIHHLCYWELMWVYSFEQNWFEAYRYANLLSKENKWSQAVYVFQKAAILSMMPEEEVNKLNENVVELFRQVEGLRLRFAGKSIPTEKFAAKKAQRYSSST?VKLVVPALEMMYVWNGFTIVGKRPELTEGILSTLEKAEEQLRNDPNPS?YHTGDECVVQLLKGLCLRSLGRLVQAEICFNYVISSDGGIKHDSYLVPFTMYELGLLHKQKGEINKAIAVMENVMTNYKDYNMESRLHFRIHAALNTMGSFAAKLPPTRTAV*EIPLHET*QCLLFHCLLP-

>TmY4.TM1 T0000032630-R1

---MEGEDNSLQQVGLKQPNEDTILPEELPKMGLETALKECFTALNFFLNNRFADALALLKPWKSESMYHAMGYSSILVMQAAMTFDAKDMDAAMTSLREALQVCQRFRKKTGLMESLANLWYRQPADNLTEEEMHAELCYAEVLLQKAALTFLDESIIGFIKGGMKIRHSYQLYKDCQAMEKVTKDEEKQRSAHIHFRGGVSMGIGSFNLMLSLLPSRVLRLMEFLGFSGDREVGLSELREGATTNNLRSILSTLTLLMFQLYITVILGTADVNLSECEALLKPYCEKFPNGALMHFFSARIAVLKGNFTFAQEKFLACIASQEEWRQIHHLCYWELMWVYSFEQNWFEAYRYANLLSKENKWSQAVYVFQKAAILSMMPEEEVNKLNENVVELFRQVEGLRLRFAGKSIPTEKFAAKKAQRYSSSTPVKLVVPALEMMYVWNGFTIVGKRPELTEGILSTLEKAEEQLRNDPNPSDYHTGDECVVQLLKGLCLRSLGRLVQAEICFNYVISSDGGIKHDSYLVPFTMYELGLLHKQKGEINKAIAVMENVMTNYKDYNMESRLHFRIHAALNTMGSFAAKLPPTRTAV*EIPLHET*QCLLFHCLLP?

>TmY5.TM1 T0000032630-R1

---MEGEDNSLQQVGLKQPNEDTILPEELPKMGLETALKECFTALNFFLNNRFADALALLKPWKSESMYHAMGYSSILVMQAAMTFDAKDMDAAMTSLREALQVCQRFRKKTGLMESLANLWYRQPADNLTEEEMHAELCYAEVLLQKAALTFLDESIIGFIKGGMKIRHSYQLYKDCQAMEKVTKDEEKQRSAHIHFRGGVSMGIGSFNLMLSLLPSRVLRLMEFLGFSGDREVGLSELREGATTNNLRSILSTLTLLMFQLYITVILGTADVNLSECEALLKPYCEKFPNGALMHFFSARIAVLKGNFTFAQEKFLA?IA?QEEWRQIHHLCYWELMWVYSFEQNWFEAYRYANLLSKENKWSQAVYVFQKAAILSMMPEEEVNKLNENVVELFRQVEGLRLRFAGKSIPTEKFAAKKAQRYSSST?VKLVVPALEMMYVWNGFTIVGKRPELTEGILSTLEKAEEQLRNDPNPS?YHTGDECVVQLLKGLCLRSLGRLVQAEICFNYVISSDGGIKHDSYLVPFTMYELGLLHKQKGEINKAIAVMENVMTNYKDYNMESRLHFRIHAALNTMGSFAAKLPPTRTAV*EIPLHET*QCLLFHCLLP?

>TmY6.TM1 T0000032630-R1

---MEGEDNSLQQVGLKQPNEDTILPEELPKMGLETALKECFTALNFFLNNRFADALALLKPWKSESMYHAMGYSSILVMQAAMTFDAKDMDAAMTSLREALQVCQRFRKKTGLMESLANLWYRQPADNLTEEEMHAELCYAEVLLQKAALTFLDESIIGFIKGGMKIRHSYQLYKDCQAMEKVTKDEEKQRSAHIHFRGGVSMGIGSFNLMLSLLPSRVLRLMEFLGFSGDREVGLSELREGATTNNLRSILSTLTLLMFQLYITVILGTADVNLSECEALLKPYCEKFPNGALMHFFSARIAVLKGNFTFAQEKFLA?IA?QEEWRQIHHLCYWELMWVYSFEQNWFEAYRYANLLSKENKWSQAVYVFQKAAILSMMPEEEVNKLNENVVELFRQVEGLRLRFAGKSIPTEKFAAKKAQRYSSST?VKLVVPALEMMYVWNGFTIVGKRPELTEGILSTLEKAEEQLRNDPNPS?YHTGDECVVQLLKGLCLRSLGRLVQAEICFNYVISSDGGIKHDSYLVPFTMYELGLLHKQKGEINKAIAVMENVMTNYKDYNMESRLHFRIHAALNTMGSFAAKLPPTRTAV*EIPLHET*QCLLFHC?---

***ttc39b*, DNA sequence alignment in fasta format**

>TM1 T0000032630-R1

---------ATGGAGGGTGAAGACAATTCACTACAACAGGTGGGTTTGAAGCAGCCCAATGAGGATACAATATTGCCTGAAGAATTGCCTAAAATGGGTTTGGAGACAGCATTGAAAGAATGCTTCACTGCCCTCAATTTCTTCCTGAACAACAGGTTTGCCGATGCATTGGCTCTTTTAAAACCCTGGAAGAGTGAAAGTATGTACCATGCGATGGGCTACAGTAGCATTCTGGTGATGCAGGCCGCCATGACTTTTGATGCAAAGGACATGGATGCTGCCATGACATCACTGAGAGAAGCCTTGCAGGTCTGCCAGAGATTTCGGAAGAAAACGGGACTAATGGAAAGCTTGGCTAACCTTTGGTACAGACAACCAGCTGACAATCTGACAGAAGAAGAGATGCATGCAGAGCTGTGCTATGCTGAAGTCCTGCTGCAGAAAGCTGCCCTCACGTTCTTGGATGAAAGCATAATAGGCTTCATCAAAGGAGGGATGAAAATTCGACACAGTTATCAGCTTTACAAGGATTGCCAGGCCATGGAAAAAGTCACAAAGGATGAGGAAAAACAGAGAAGCGCACACATTCATTTTAGGGGTGGGGTCAGCATGGGAATTGGATCATTCAATCTGATGCTGTCTCTGCTTCCGTCCAGAGTCCTTAGACTGATGGAGTTTTTGGGCTTCTCTGGAGACAGGGAAGTGGGTTTGTCAGAGTTGAGAGAGGGAGCAACTACCAACAACCTGCGCTCCATCCTCAGCACCCTCACTCTGCTGATGTTTCAGCTCTACATCACAGTGATACTTGGGACTGCTGATGTAAACCTAAGTGAATGTGAGGCTCTGCTGAAACCCTACTGTGAAAAGTTTCCTAATGGGGCTTTAATGCATTTCTTCAGTGCAAGGATTGCTGTGCTCAAAGGAAACTTCACATTTGCCCAAGAGAAGTTCTTGGCATGTATTGCATCGCAGGAAGAGTGGCGTCAGATTCACCACCTGTGCTACTGGGAGCTGATGTGGGTCTACTCCTTTGAACAAAACTGGTTTGAGGCCTATCGATACGCCAACCTCCTCAGCAAGGAGAACAAGTGGTCCCAGGCAGTCTATGTATTTCAGAAAGCTGCCATCTTGAGCATGATGCCAGAGGAAGAAGTGAACAAGCTCAATGAAAATGTGGTGGAATTATTCAGGCAGGTGGAGGGCCTCAGGCTGAGATTTGCTGGGAAGTCGATTCCAACGGAGAAGTTTGCAGCGAAGAAGGCCCAGAGATACTCCTCTTCCACCCCCGTGAAACTTGTCGTTCCTGCTTTGGAAATGATGTATGTGTGGAATGGCTTCACAATAGTAGGCAAAAGACCCGAGCTGACTGAAGGAATCCTGTCAACTTTAGAGAAAGCAGAAGAGCAGCTCAGAAATGATCCGAACCCATCAGATTACCACACGGGCGATGAGTGCGTTGTCCAGCTGCTGAAGGGTCTGTGCTTAAGAAGTCTGGGACGGCTGGTCCAGGCTGAGATCTGCTTCAATTATGTAATCTCCAGTGATGGTGGCATCAAGCATGATAGCTATCTGGTGCCCTTTACCATGTATGAGCTTGGCCTATTGCACAAGCAGAAAGGTGAAATCAACAAGGCCATTGCTGTAATGGAAAATGTCATGACGAACTACAAGGACTACAACATGGAGTCAAGGCTGCATTTCCGCATCCATGCAGCACTCAACACCATGGGCTCCTTTGCAGCCAAACTTCCACCAACACGCACGGCAGTTTAAGAGATCCCATTACATGAAACTTGACAGTGTTTACTGTTTCATTGTCTGTTGCCTTCA

>ENSMZET00005027059.1 cds primary assembly:M zebra UMD2a:LG6:16158408:16164946:1 gene:ENSMZEG00005019551.1 gene biotype:protein coding transcript biotype:protein coding gene symbol:TTC39B description:tetratricopeptide repeat protein 39B Source:NCBI gene Acc:101486776

ATGCATCAAATGGAGGGTGAAGACAATTCACTACAACAGGTGGGTTTGAAGCAGCCCAATGAGGATGCAATATTGCCTGAAGAATTGCCTAAAATGGCTTTGGAGACAGCATTGAAAGAATGCTTCACTGCCCTCAATTTCTTCCTGAACAACAGGTTTGCCGATGCACTGGCTCTTTTAAAACCCTGGAAGAGTGAAAGCATGTACCATGCGATGGGCTACAGTAGCATTCTGGTGATGCAGGCCGCTGTGACTTTTGATGCAAAGGACATGGATGCTGCCATGACATCACTGAGAGAAGCCTTGCAGGTCTGCCAGAGATTTCGGAAGAAAACGGGACTAATGGAAAGCTTGGCTAACCTTTGGTACAGACAACCAGCTGACAATCTGACAGAAGAAGAGATGCATGCAGAGCTGTGCTATGCTGAAGTCCTGCTGCAGAAAGCTGCCCTCACGTTCTTGGATGAAAGCATAATAGGCTTCATCAAAGGAGGGATGAAAATTCGACACAGTTATCAGCTTTACAAGAATTGCCAGGCCATGGAAAAAGTCACAAAGGATGAGGAAAAACAGAGAAGCACACACATTCATTTTAGGGGTGGGGTCAGCATGGGAATTGGATCATTCAATCTGATGCTGTCTCTGCTTCCATCCAGAGTCCTTAGACTGATGGAGTTTTTGGGCTTCTCTGGAGACAGGGAAGTGGGTTTGTCAGAGTTGAGAGAGGGAGCAACTACCAACAACCTGCGCTCCATCCTCAGCACCCTCACTCTGCTGATGTTTCAGCTCTACATCACAGTGATACTTGGGACTGCTGATGTAAACCTAAGTGAATGTGAGGCTCTGCTGAAACCCTACTGTGAAAAGTTTCCTAATGGGGCTTTAATGCATTTCTTCAGTGCAAGGATTGCTGTGCTCAAAGGAAACTTCACATTTGCCCAAGAGAAGTTCTTGGCATGTATTGCATCGCAGGAAGAGTGGCGTCAGATTCACCACCTGTGCTACTGGGAGCTGATGTGGGTCTACTCCTTTGAACAAAACTGGTTTGAGGCCTATCGATACGCCAACTTGCTCAGCAAGGAGAACAAGTGGTCCCAGGCAGTCTATGTATTTCAGAAAGCTGCCATCTTGAGCATGATGCCAGAGGAAGAAGTGAACAAGTTCAATGAAAATGTGGTGGAATTATTCAGGCAGGTGGAGGGCCTCAGGCTGAGATTTGCTGGGAAGTCGATTCCAACGGAGAAGTTTGCAGCGAAGAAGGCCCAGAGATACTCCTCTTCCACCCCCGTGAAACTTGTCGTTCCTGCTTTGGAAATGATGTATGTGTGGAATGGCTTCACAATAGTAGGCAAAAAACCCGAGCTGACTGAAGGAATCCTGTCAACTTTAGAGAAAGCAGAAGAGCAGCTCAGAAATGATCCAAACCCATCAGAGTACCACACAGGCGATGAGTGCATTGTCCAGCTGCTGAAGGGTCTGTGCTTAAGAAGTCTGGGACGGCTGGTCCAGGCTGAGATCTGCTTCAATCATGTCATCTCCAGTGATGGTGGCATCAAGCATGATAGCTATCTGGTGCCCTTTACCATGTATGAGCTTGGCCTATTGCACAAGCAGAAAGGTGAAATCAACAAGGCCATTGCTGTAATGGAAAATGTCATGACGAACTACAAGGACTACAACATGGAGTCAAGGCTGCATTTCCGCATCCATGCAGCACTCAACACCATGGGCTCCTTTGCAGCCAAACTTCCACCATCACGCACGGCAGCTTAA---------------------------------------------------------

>AhR1.ENSMZET00005027059.1

ATGCATCAAATGGAGGGTGAAGACAATTCACTACAACAGGTGGGTTTGAAGCAGCCCAATGAGGATGCAATATTGCCTGAAGAATTGCCTAAAATGGCTTTGGAGACAGCATTGAAAGAATGCTTCACTGCCCTCAATTTCTTCCTGAACAACAGGTTTGCCGATGCACTGGCTCTTTTAAAACCCTGGAAGAGTGAAAGCATGTACCATGCGATGGGCTACAGTAGCATTCTGGTGATGCAGGCCGCTGTGACTTTTGATGCAAAGGACATGGATGCTGCCATGACATCACTGAGAGAAGCCTTGCAGGTCTGCCAGAGATTTCGGAAGAAAACKGGACTAATGGAAAGCTTGGCTAACCTTTGGTACAGACAACCAGCTGACAATCTGACAGAAGAAGAGATGCATGCAGAGCTGTGCTATGCTGAAGTCCTGCTGCAGAAAGCTGCCCTCACGTTCTTGGATGAAAGCATAATAGGCTTCATCAAAGGAGGGATGAAAATTCGACACAGTTATCAGCTTTACAAGAATTGCCAGGCCATGGAAAAAGTCACAAAGGATGAGGAAAAACAGAGAAGCACACACATTCATTTTAGGGGTGGGGTCAGCATGGGAATTGGATCATTCAATCTGATGCTGTCTCTGCTTCCATCCAGAGTCCTTAGACTGATGGAGTTTTTGGGCTTCTCTGGAGACAGGGAAGTGGGTTTGTCAGAGTTGAGAGAGGGAGCAACTACCAACAACCTGCGCTCCATCCTCAGCACCCTCACTCTGCTGATGTTTCAGCTCTACATCACAGTGATACTTGGGACTGCTGATGTAAACCTAAGTGAATGTGAGGCTCTGCTGAAACCCTACTGTGAAAAGTTTCCTAATGGGGCTTTAATGCATTTCTTCAGTGCAAGGATTGCTGTGCTCAAAGGAAACTTCACATTTGCCCAAGAGAAGTTCTTGGCATGTATTACATCGCAGGAAGAGTGGCGTCAGATTCACCACCTGTGCTACTGGGAGCTGATGTGGGTCTACTCCTTTGAACAAAACTGGTTTGAGGCCTATCGATACGCCAACTTGCTCAGCAAGGAGAACAAGTGGTCCCAGGCAGTCTATGTATTTCAGAAAGCTGCCATCTTGAGCATGATGCCAGAGGAAGAAGTGAACAAGTTCAATGAAAATGTGGTGGAATTATTCAGGCAGGTGGAGGGCCTCAGGCTGAGATTTGCTGGGAAGTCGATTCCAACGGAGAAGTTTGCAGCGAAGAAGGCCCAGAGATACTCCTCTTCCACCCCCGTGAAACTTGTCGTTCCTGCTTTGGAAATGATGTATGTGTGGAATGGCTTCACAATAGTAGGCAAAAAACCCGAGCTGACTGAAGGAATCCTGTCAACTTTAGAGAAAGCAGAAGAGCAGCTCAGAAATGATCCAAACCCATCAGAGTACCACACAGGCGATGAGTGCGTTGTCCAGCTGCTGAAGGGTCTGTGCTTAAGAAGTCTGGGACGGCTGGTCCAGGCTGAGATCTGCTTCAATCATGTCATCTCCAGTGATGGTGGCATCAAGCATGATAGCTATCTGGTGCCCTTTACCATGTATGAGCTTGGCCTATTGCACAAGCAGAAAGGTGAAATCAACAAGGCCATTGCTGTAATGGAAAATGTCATGACGAACTACAAGSACTACAACATGGAGTCAAGGCTGCATTTCCGCATCCATGCAGCACTCAACACCATGGGCTCCTTTGCAGCCAAACTTCCACCATCACGCACGGCAGCTTAA---------------------------------------------------------

>AhR2.ENSMZET00005027059.1

ATGCATCAAATGGAGGGTGAAGACAATTCACTACAACAGGTGGGTTTGAAGCAGCCCAATGAGGATGCAATATTGCCTGAAGAATTGCCTAAAATGGCTTTGGAGACAGCATTGAAAGAATGCTTCACTGCCCTCAATTTCTTCCTGAACAACAGGTTTGCCGATGCACTGGCTCTTTTAAAACCCTGGAAGAGTGAAAGCATGTACCATGCGATGGGCTACAGTAGCATTCTGGTGATGCAGGCCGCTGTGACTTTTGATGCAAAGGACATGGATGCTGCCATGACATCACTGAGAGAAGCCTTGCAGGTCTGCCAGAGATTTCGGAAGAAAACKGGACTAATGGAAAGCTTGGCTAACCTTTGGTACAGACAACCAGCTGACAATCTGACAGAAGAAGAGATGCATGCAGAGCTGTGCTATGCTGAAGTCCTGCTGCAGAAAGCTGCCCTCRCGTTCTTGGATGAAAGCATAATAGGCTTCATCAAAGGAGGGATGAAAATTCGACACAGTTATCAGCTTTACAAGAATTGCCAGGCCATGGAAAAAGTCACAAAGGATGAGGAAAAACAGAGAAGCACACACATTCATTTTAGGGGTGGGGTCAGCATGGGAATTGGATCATTCAATCTGATGCTGTCTCTGCTTCCATCCAGAGTCCTTAGACTGATGGAGTTTTTGGGCTTCTCTGGAGACAGGGAAGTGGGTTTGTCAGAGTTGAGAGAGGGAGCAACTACCAACAACCTGCGCTCCATCCTCAGCACCCTCACTCTGCTGATGTTTCAGCTCTACATCACAGTGATACTTGGGACTGCTGATGTAAACCTAAGTGAATGTGAGGCTCTGCTGAAACCCTACTGTGAAAAGTTTCCTAATGGGGCTTTAATGCATTTCTTCAGTGCAAGGATTGCTGTGCTCAAAGGAAACTTCACATTTGCCCAAGAGAAGTTCTTGGCATGTATTACATCGCAGGAAGAGTGGCGTCAGATTCACCACCTGTGCTACTGGGAGCTGATGTGGGTCTACTCCTTTGAACAAAACTGGTTTGAGGCCTATCGATACGCCAACTTGCTCAGCAAGGAGAACAAGTGGTCCCAGGCAGTCTATGTATTTCAGAAAGCTGCCATCTTGAGCATGATGCCAGAGGAAGAAGTGAACAAGTTCAATGAAAATGTGGTGGAATTATTCAGGCAGGTGGAGGGCCTCAGGCTGAGATTTGCTGGGAAGTCGATTCCAACGGAGAAGTTTGCAGCGAAGAAGGCCCAGAGATACTCCTCTTCCACCCCCGTGAAACTTGTCGTTCCTGCTTTGGAAATGATGTATGTGTGGAATGGCTTCACAATAGTAGGCAAAAAACCCGAGCTGACTGAAGGAATCCTGTCAACTTTAGAGAAAGCAGAAGAGCAGCTCAGAAATGATCCAAACCCATCAGAGTACCACACAGGCGATGAGTGCGTTGTCCAGCTGCTGAAGGGTCTGTGCTTAAGAAGTCTGGGACGGCTGGTCCAGGCTGAGATCTGCTTCAATCATGTCATCTCCAGTGATGGTGGCATCAAGCATGATAGCTATCTGGTGCCCTTTACCATGTATGAGCTTGGCCTATTGCACAAGCAGAAAGGTGAAATCAACAAGGCCATTGCTGTAATGGAAAATGTCATGACGAACTACAAGSACTACAACATGGAGTCAAGGCTGCATTTCCGCATCCATGCAGCACTCAACACCATGGGCTCCTTTGCAGCCAAACTTCCACCATCACGCACGGCAGCTTAA---------------------------------------------------------

>AhR3.ENSMZET00005027059.1

ATGCATCAAATGGAGGGTGAAGACAATTCACTACAACAGGTGGGTTTGAAGCAGCCCAATGAGGATGCAATATTGCCTGAAGAATTGCCTAAAATGGCTTTGGAGACAGCATTGAAAGAATGCTTCACTGCCCTCAATTTCTTCCTGAACAACAGGTTTGCCGATGCACTGGCTCTTTTAAAACCCTGGAAGAGTGAAAGCATGTACCATGCGATGGGCTACAGTAGCATTCTGGTGATGCAGGCCGCTGTGACTTTTGATGCAAAGGACATGGATGCTGCCATGACATCACTGAGAGAAGCCTTGCAGGTCTGCCAGAGATTTCGGAAGAAAACGGGACTAATGGAAAGCTTGGCTAACCTTTGGTACAGACAACCAGCTGACAATCTGACAGAAGAAGAGATGCATGCAGAGCTGTGCTATGCTGAAGTCCTGCTGCAGAAAGCTGCCCTCACGTTCTTGGATGAAAGCATAATAGGCTTCATCAAAGGAGGGATGAAAATTCGACACAGTTATCAGCTTTACAAGAATTGCCAGGCCATGGAAAAAGTCACAAAGGATGAGGAAAAACAGAGAAGCACACACATTCATTTTAGGGGTGGGGTCAGCATGGGAATTGGATCATTCAATCTGATGCTGTCTCTGCTTCCATCCAGAGTCCTTAGACTGATGGAGTTTTTGGGCTTCTCTGGAGACAGGGAAGTGGGTTTGTCAGAGTTGAGAGAGGGAGCAACTACCAACAACCTGCGCTCCATCCTCAGCACCCTCACTCTGCTGATGTTTCAGCTCTACATCACAGTGATACTTGGGACTGCTGATGTAAACCTAAGTGAATGTGAGGCTCTGCTGAAACCCTACTGTGAAAAGTTTCCTAATGGGGCTTTAATGCATTTCTTCAGTGCAAGGATTGCTGTGCTCAAAGGAAACTTCACATTTGCCCAAGAGAAGTTCTTGGCATGTATTACATCGCAGGAAGAGTGGCGTCAGATTCACCACCTGTGCTACTGGGAGCTGATGTGGGTCTACTCCTTTGAACAAAACTGGTTTGAGGCCTATCGATACGCCAACTTGCTCAGCAAGGAGAACAAGTGGTCCCAGGCAGTCTATGTATTTCAGAAAGCTGCCATCTTGAGCATGATGCCAGAGGAAGAAGTGAACAAGTTCAATGAAAATGTGGTGGAATTATTCAGGCAGGTGGAGGGCCTCAGGCTGAGATTTGCTGGGAAGTCGATTCCAACGGAGAAGTTTGCAGCGAAGAAGGCCCAGAGATACTCCTCTTCCACCCCCGTGAAACTTGTCGTTCCTGCTTTGGAAATGATGTATGTGTGGAATGGCTTCACAATAGTAGGCAAAAAACCCGAGCTGACTGAAGGAATCCTGTCAACTTTAGAGAAAGCAGAAGAGCAGCTCAGAAATGATCCAAACCCATCAGAGTACCACACAGGCGATGAGTGCGTTGTCCAGCTGCTGAAGGGTCTGTGCTTAAGAAGTCTGGGACGGCTGGTCCAGGCTGAGATCTGCTTCAATCATGTCATCTCCAGTGATGGTGGCATCAAGCATGATAGCTATCTGGTGCCCTTTACCATGTATGAGCTTGGCCTATTGCACAAGCAGAAAGGTGAAATCAACAAGGCCATTGCTGTAATGGAAAATGTCATGACGAACTACAAGCACTACAACATGGAGTCAAGGCTGCATTTCCGCATCCATGCAGCACTCAACACCATGGGCTCCTTTGCAGCCAAACTTCCACCATCACGCACGGCAGCTTAA---------------------------------------------------------

>AhR4.ENSMZET00005027059.1

ATGCATCAAATGGAGGGTGAAGACAATTCACTACAACAGGTGGGTTTGAAGCAGCCCAATGAGGATGCAATATTGCCTGAAGAATTGCCTAAAATGGCTTTGGAGACAGCATTGAAAGAATGCTTCACTGCCCTCARTTTCTTCCTGAACAACAGGTTTGCCGATGCACTGGCTCTTTTAAAACCCTGGAAGAGTGAAAGCATGTACCATGCGATGGGCTACAGTAGCATTCTGGTGATGCAGGCCGCTGTGACTTTTGATGCAAAGGACATGGATGCTGCCATGACATCACTGAGAGAAGCCTTGCAGGTCTGCCAGAGATTTCGGAAGAAAACKGGACTAATGGAAAGCTTGGCTAACCTTTGGTACAGACAACCAGCTGACAATCTGACAGAAGAAGAGATGCATGCAGAGCTGTGCTATGCTGAAGTCCTGCTGCAGAAAGCTGCCCTCACGTTCTTGGATGAAAGCATAATAGGCTTCATCAAAGGAGGGATGAAAATTCGACACAGTTATCAGCTTTACAAGAATTGCCAGGCCATGGAAAAAGTCACAAAGGATGAGGAAAAACAGAGAAGCACACACATTCATTTTAGGGGTGGGGTCAGCATGGGAATTGGATCATTCAATCTGATGCTGTCTCTGCTTCCATCCAGAGTCCTTAGACTGATGGAGTTTTTGGGCTTCTCTGGAGACAGGGAAGTGGGTTTGTCAGAGTTGAGAGAGGGAGCAACTACCAACAACCTGCGCTCCATCCTCAGCACCCTCACTCTGCTGATGTTTCAGCTCTACATCACAGTGATACTTGGGACTGCTGATGTAAACCTAAGTGAATGTGAGGCTCTGCTGAAACCCTACTGTGAAAAGTTTCCTAATGGGGCTTTAATGCATTTCTTCAGTGCAAGGATTGCTGTGCTCAAAGGAAACTTCACATTTGCCCAAGAGAAGTTCTTGGCATGTATTACATCGCAGGAAGAGTGGCGTCAGATTCACCACCTGTGCTACTGGGAGCTGATGTGGGTCTACTCCTTTGAACAAAACTGGTTTGAGGCCTATCGATACGCCAACTTGCTCAGCAAGGAGAACAAGTGGTCCCAGGCAGTCTATGTATTTCAGAAAGCTGCCATCTTGAGCATGATGCCAGAGGAAGAAGTGAACAAGTTCAATGAAAATGTGGTGGAATTATTCAGGCAGGTGGAGGGCCTCAGGCTGAGATTTGCTGGGAAGTCGATTCCAACGGAGAAGTTTGCAGCGAAGAAGGCCCAGAGATACTCCTCTTCCACCCCCGTGAAACTTGTCGTTCCTGCTTTGGAAATGATGTATGTGTGGAATGGCTTCACAATAGTAGGCAAAAAACCCGAGCTGACTGAAGGAATCCTGTCAACTTTAGAGAAAGCAGAAGAGCAGCTCAGAAATGATCCAAACCCATCAGAGTACCACACAGGCGATGAGTGCGTTGTCCAGCTGCTGAAGGGTCTGTGCTTAAGAAGTCTGGGACGGCTGGTCCAGGCTGAGATCTGCTTCAATCATGTCATCTCCAGTGATGGTGGCATCAAGCATGATAGCTATCTGGTGCCCTTTACCATGTATGAGCTTGGCCTATTGCACAAGCAGAAAGGTGAAATCAACAAGGCCATTGCTGTAATGGAAAATGTCATGACGAACTACAAGSACTACAACATGGAGTCAAGGCTGCATTTCCGCATCCATGCAGCACTCAACACCATGGGCTCCTTTGCAGCCAAACTTCCACCATCACGCACGGCAGCTTAA---------------------------------------------------------

>AhR5.ENSMZET00005027059.1

ATGCATCAAATGGAGGGTGAAGACAATTCACTACAACAGGTGGGTTTGAAGCAGCCCAATGAGGATGCAATATTGCCTGAAGAATTGCCTAAAATGGCTTTGGAGACAGCATTGAAAGAATGCTTCACTGCCCTCAATTTCTTCCTGAACAACAGGTTTGCCGATGCACTGGCTCTTTTAAAACCCTGGAAGAGTGAAAGCATGTACCATGCGATGGGCTACAGTAGCATTCTGGTGATGCAGGCCGCTGTGACTTTTGATGCAAAGGACATGGATGCTGCCATGACATCACTGAGAGAAGCCTTGCAGGTCTGCCAGAGATTTCGGAAGAAAACGGGACTAATGGAAAGCTTGGCTAACCTTTGGTACAGACAACCAGCTGACAATCTGACAGAAGAAGAGATGCATGCAGAGCTGTGCTATGCTGAAGTCCTGCTGCAGAAAGCTGCCCTCACGTTCTTGGATGAAAGCATAATAGGCTTCATCAAAGGAGGGATGAAAATTCGACACAGTTATCAGCTTTACAAGAATTGCCAGGCCATGGAAAAAGTCACAAAGGATGAGGAAAAACAGAGAAGCACACACATTCATTTTAGGGGTGGGGTCAGCATGGGAATTGGATCATTCAATCTGATGCTGTCTCTGCTTCCATCCAGAGTCCTTAGACTGATGGAGTTTTTGGGCTTCTCTGGAGACAGGGAAGTGGGTTTGTCAGAGTTGAGAGAGGGAGCAACTACCAACAACCTGCGCTCCATCCTCAGCACCCTCACTCTGCTGATGTTTCAGCTCTACATCACAGTGATACTTGGGACTGCTGATGTAAACCTAAGTGAATGTGAGGCTCTGCTGAAACCCTACTGTGAAAAGTTTCCTAATGGGGCTTTAATGCATTTCTTCAGTGCAAGGATTGCTGTGCTCAAAGGAAACTTCACATTTGCCCAAGAGAAGTTCTTGGCATGTATTACATCGCAGGAAGAGTGGCGTCAGATTCACCACCTGTGCTACTGGGAGCTGATGTGGGTCTACTCCTTTGAACAAAACTGGTTTGAGGCCTATCGATACGCCAACTTGCTCAGCAAGGAGAACAAGTGGTCCCAGGCAGTCTATGTATTTCAGAAAGCTGCCATCTTGAGCATGATGCCAGAGGAAGAAGTGAACAAGTTCAATGAAAATGTGGTGGAATTATTCAGGCAGGTGGAGGGCCTCAGGCTGAGATTTGCTGGGAAGTCGATTCCAACGGAGAAGTTTGCAGCGAAGAAGGCCCAGAGATACTCCTCTTCCACCCCCGTGAAACTTGTCGTTCCTGCTTTGGAAATGATGTATGTGTGGAATGGCTTCACAATAGTAGGCAAAAAACCCGAGCTGACTGAAGGAATCCTGTCAACTTTAGAGAAAGCAGAAGAGCAGCTCAGAAATGATCCAAACCCATCAGAGTACCACACAGGCGATGAGTGCGTTGTCCAGCTGCTGAAGGGTCTGTGCTTAAGAAGTCTGGGACGGCTGGTCCAGGCTGAGATCTGCTTCAATCATGTCATCTCCAGTGATGGTGGCATCAAGCATGATAGCTATCTGGTGCCCTTTACCATGTATGAGCTTGGCCTATTGCACAAGCAGAAAGGTGAAATCAACAAGGCCATTGCTGTAATGGAAAATGTCATGACGAACTACAAGCACTACAACATGGAGTCAAGGCTGCATTTCCGCATCCATGCAGCACTCAACACCATGGGCTCCTTTGCAGCCAAACTTCCACCATCACGCACGGCAGCTTAA---------------------------------------------------------

>AhR6.ENSMZET00005027059.1

ATGCATCAAATGGAGGGTGAAGACAATTCACTACAACAGGTGGGTTTGAAGCAGCCCAATGAGGATGCAATATTGCCTGAAGAATTGCCTAAAATGGCTTTGGAGACAGCATTGAAAGAATGCTTCACTGCCCTCAATTTCTTCCTGAACAACAGGTTTGCCGATGCACTGGCTCTTTTAAAACCCTGGAAGAGTGAAAGCATGTACCATGCGATGGGCTACAGTAGCATTCTGGTGATGCAGGCCGCTGTGACTTTTGATGCAAAGGACATGGATGCTGCCATGACATCACTGAGAGAAGCCTTGCAGGTCTGCCAGAGATTTCGGAAGAAAACKGGACTAATGGAAAGCTTGGCTAACCTTTGGTACAGACAACCAGCTGACAATCTGACAGAAGAAGAGATGCATGCAGAGCTGTGCTATGCTGAAGTCCTGCTGCAGAAAGCTGCCCTCRCGTTCTTGGATGAAAGCATAATAGGCTTCATCAAAGGAGGGATGAAAATTCGACACAGTTATCAGCTTTACAAGAATTGCCAGGCCATGGAAAAAGTCACAAAGGATGAGGAAAAACAGAGAAGCACACACATTCATTTTAGGGGTGGGGTCAGCATGGGAATTGGATCATTCAATCTGATGCTGTCTCTGCTTCCATCCAGAGTCCTTAGACTGATGGAGTTTTTGGGCTTCTCTGGAGACAGGGAAGTGGGTTTGTCAGAGTTGAGAGAGGGAGCAACTACCAACAACCTGCGCTCCATCCTCAGCACCCTCACTCTGCTGATGTTTCAGCTCTACATCACAGTGATACTTGGGACTGCTGATGTAAACCTAAGTGAATGTGAGGCTCTGCTGAAACCCTACTGTGAAAAGTTTCCTAATGGGGCTTTAATGCATTTCTTCAGTGCAAGGATTGCTGTGCTCAAAGGAAACTTCACATTTGCCCAAGAGAAGTTCTTGGCATGTATTRCATCGCAGGAAGAGTGGCGTCAGATTCACCACCTGTGCTACTGGGAGCTGATGTGGGTCTACTCCTTTGAACAAAACTGGTTTGAGGCCTATCGATACGCCAACTTGCTCAGCAAGGAGAACAAGTGGTCCCAGGCAGTCTATGTATTTCAGAAAGCTGCCATCTTGAGCATGATGCCAGAGGAAGAAGTGAACAAGTTCAATGAAAATGTGGTGGAATTATTCAGGCAGGTGGAGGGCCTCAGGCTGAGATTTGCTGGGAAGTCGATTCCAACGGAGAAGTTTGCAGCGAAGAAGGCCCAGAGATACTCCTCTTCCACCCCCGTGAAACTTGTCGTTCCTGCTTTGGAAATGATGTATGTGTGGAATGGCTTCACAATAGTAGGCAAAAAACCCGAGCTGACTGAAGGAATCCTGTCAACTTTAGAGAAAGCAGAAGAGCAGCTCAGAAATGATCCAAACCCATCAGAGTACCACACAGGCGATGAGTGCGTTGTCCAGCTGCTGAAGGGTCTGTGCTTAAGAAGTCTGGGACGGCTGGTCCAGGCTGAGATCTGCTTCAATCATGTCATCTCCAGTGATGGTGGCATCAAGCATGATAGCTATCTGGTGCCCTTTACCATGTATGAGCTTGGCCTATTGCACAAGCAGAAAGGTGAAATCAACAAGGCCATTGCTGTAATGGAAAATGTCATGACGAACTACAAGGACTACAACATGGAGTCAAGGCTGCATTTCCGCATCCATGCAGCACTCAACACCATGGGCTCCTTTGCAGCCAAACTTCCACCATCAYGCACGGCAGCTTAA---------------------------------------------------------

>AbY1.ENSMZET00005027059.1

ATGCATCAAATGGAGGGTGAAGACAATTCACTACAACAGGTGGGTTTGAAGCAGCCCAATGAGGATGCAATATTGCCTGAAGAATTGCCTAAAATGGCTTTGGAGACAGCATTGAAAGAATGCTTCACTGCCCTCAATTTCTTCCTGAACAACAGGTTTGCCGATGCACTGGCTCTTTTAAAACCCTGGAAGAGTGAAAGCATGTACCATGCGATGGGCTACAGTAGCATTCTGGTGATGCAGGCCGCTGTGACTTTTGATGCAAAGGACATGGATGCTGCCATGACATCACTGAGAGAAGCCTTGCAGGTCTGCCAGAGATTTCGGAAGAAAACGGGACTAATGGAAAGCTTGGCTAACCTTTGGTACAGACAACCAGCTGACAATCTGACAGAAGAAGAGATGCATGCAGAGCTGTGCTATGCTGAAGTCCTGCTGCAGAAAGCTGCCCTCACGTTCTTGGATGAAAGCATAATAGGCTTCATCAAAGGAGGGATGAAAATTCGACACAGTTATCAGCTTTACAAGAATTGCCAGGCCATGGAAAAAGTCACAAAGGATGAGGAAAAACAGAGAAGCACACACATTCATTTTAGGGGTGGGGTCAGCATGGGAATTGGATCATTCAATCTGATGCTGTCTCTGCTTCCATCCAGAGTCCTTAGACTGATGGAGTTTTTGGGCTTCTCTGGAGACAGGGAAGTGGGTTTGTCAGAGTTGAGAGAGGGAGCAACTACCAACAACCTGCGCTCCATCCTCAGCACCCTCACTCTGCTGATGTTTCAGCTCTACATCACAGTGATACTTGGGACTGCTGATGTAAACCTAAGTGAATGTGAGGCTCTGCTGAAACCCTACTGTGAAAAGTTTCCTAATGGGGCTTTAATGCATTTCTTCAGTGCAAGGATTGCTGTGCTCAAAGGAAACTTCACATTTGCCCAAGAGAAGTTCTTGGCATGTATTGCATCGCAGGAAGAGTGGCGTCAGATTCACCACCTGTGCTACTGGGAGCTGATGTGGGTCTACTCCTTTGAACAAAACTGGTTTGAGGCCTATCGATACGCCAACTTGCTCAGCAAGGAGAACAAGTGGTCCCAGGCAGTCTATGTATTTCAGAAAGCTGCCATCTTGAGCATGATGCCAGAGGAAGAAGTGAACAAGTTCAATGAAAATGTGGTGGAATTATTCAGGCAGGTGGAGGGCCTCAGGCTGAGATTTGCTGGGAAGTCGATTCCAACGGAGAAGTTTGCAGCGAAGAAGGCCCAGAGATACTCCTCTTCCACCCCCGTGAAACTTGTCGTTCCTGCTTTGGAAATGATGTATGTGTGGAATGGCTTCACAATAGTAGGCAAAAAACCCRAGCTGACTGAAGGAATCCTGTCAACTTTAGAGAAAGCAGAAGAGCAGCTCAGAAATGATCCAAACCCATCAGAGTACCACACAGGCGATGAGTGCGTTGTCCAGCTGCTGAAGGGTCTGTGCTTAAGAAGTCTGGGACGGCTGGTCCAGGCTGAGATCTGCTTCAATCATGTCATCTCCAGTGATGGTGGCATCAAGCATGATAGCTATCTGGTGCCCTTTACCATGTATGAGCTTGGCCTATTGCACAAGCAGAAAGGTGAAATCAACAAGGCCATTGCTGTAATGGAAAATGTCATGACGAACTACAAGGACTACAACATGGAGTCAAGGCTGCATTTCCGCATCCATGCAGCACTCAACACCATGGGCTCCTTTGCAGCCAAACTTCCACCATCATGCACGGCAGCTTAA---------------------------------------------------------

>AbY2.ENSMZET00005027059.1

ATGCATCAAATGGAGGGTGAAGACAATTCACTACAACAGGTGGGTTTGAAGCAGCCCAATGAGGATGCAATATTGCCTGAAGAATTGCCTAAAATGGCTTTGGAGACAGCATTGAAAGAATGCTTCACTGCCCTCAATTTCTTCCTGAACAACAGGTTTGCCGATGCACTGGCTCTTTTAAAACCCTGGAAGAGTGAAAGCATGTACCATGCGATGGGCTACAGTAGCATTCTGGTGATGCAGGCCGCTGTGACTTTTGATGCAAAGGACATGGATGCTGCCATGACATCACTGAGAGAAGCCTTGCAGGTCTGCCAGAGATTTCGGAAGAAAACGGGACTAATGGAAAGCTTGGCTAACCTTTGGTACAGACAACCAGCTGACAATCTGACAGAAGAAGAGATGCATGCAGAGCTGTGCTATGCTGAAGTCCTGCTGCAGAAAGCTGCCCTCACGTTCTTGGATGAAAGCATAATAGGCTTCATCAAAGGAGGGATGAAAATTCGACACAGTTATCAGCTTTACAAGAATTGCCAGGCCATGGAAAAAGTCACAAAGGATGAGGAAAAACAGAGAAGCACACACATTCATTTTAGGGGTGGGGTCAGCATGGGAATTGGATCATTCAATCTGATRCTGTCTCTGCTTCCATCCAGAGTCCTTAGACTGATGGAGTTTTTGGGCTTCTCTGGAGACAGGGAAGTGGGTTTGTCAGAGTTGAGAGAGGGAGCAACTACCAACAACCTGCGCTCCATCCTCAGCACCCTCACTCTGCTGATGTTTCAGCTCTACATCACAGTGATACTTGGGACTGCTGATGTAAACCTAAGTGAATGTGAGGCTCTGCTGAAACCCTACTGTGAAAAGTTTCCTAATGGGGCTTTAATGCATTTCTTCAGTGCAAGGATTGCTGTGCTCAAAGGAAACTTCACATTTGCCCAAGAGAAGTTCTTGGCATGTATTGCATCGCAGGAAGAGTGGCGTCAGATTCACCACCTGTGCTACTGGGAGCTGATGTGGGTCTACTCCTTTGAACAAAACTGGTTTGAGGCCTATCGATACGCCAACTTGCTCAGCAAGGAGAACAAGTGGTCCCAGGCAGTCTATGTATTTCAGAAAGCTGCCATCTTGAGCATGATGCCAGAGGAAGAAGTGAACAAGTTCAATGAAAATGTGGTGGAATTATTCAGGCAGGTGGAGGGCCTCAGGCTGAGATTTGCTGGGAAGTCGATTCCAACGGAGAAGTTTGCAGCGAAGAAGGCCCAGAGATACTCCTCTTCCACCCCCGTGAAACTTGTCGTTCCTGCTTTGGAAATGATGTATGTGTGGAATGGCTTCACAATAGTAGGCAAAAAACCCRAGCTGACTGAAGGAATCCTGTCAACTTTAGAGAAAGCAGAAGAGCAGCTCAGAAATGATCCAAACCCATCAGAGTACCACACAGGCGATGAGTGCGTTGTCCAGCTGCTGAAGGGTCTGTGCTTAAGAAGTCTGGGACGGCTGGTCCAGGCTGAGATCTGCTTCAATCATGTCATCTCCAGTGATGGTGGCATCAAGCATGATAGCTATCTGGTGCCCTTTACCATGTATGAGCTTGGCCTATTGCACAAGCAGAAAGGTGAAATCAACAAGGCCATTGCTGTAATGGAAAATGTCATGACGAACTACAAGGACTACAACATGGAGTCAAGGCTGCATTTCCGCATCCATGCAGCACTCAACACCATGGGCTCCTTTGCAGCCAAACTTCCACCATCATGCACGGCAGCTTAA---------------------------------------------------------

>AbY5.ENSMZET00005027059.1

ATGCATCAAATGGAGGGTGAAGACAATTCACTACAACAGGTGGGTTTGAAGCAGCCCAATGAGGATGCAATATTGCCTGAAGAATTGCCTAAAATGGCTTTGGAGACAGCATTGAAAGAATGCTTCACTGCCCTCAATTTCTTCCTGAACAACAGGTTTGCCGATGCACTGGCTCTTTTAAAACCCTGGAAGAGTGAAAGCATGTACCATGCGATGGGCTACAGTAGCATTCTGGTGATGCAGGCCGCTGTGACTTTTGATGCAAAGGACATGGATGCTGCCATGACATCACTGAGAGAAGCCTTGCAGGTCTGCCAGAGATTTCGGAAGAAAACGGGACTAATGGAAAGCTTGGCTAACCTTTGGTACAGACAACCAGCTGACAATCTGACAGAAGAAGAGATGCATGCAGAGCTGTGCTATGCTGAAGTCCTGCTGCAGAAAGCTGCCCTCACGTTCTTGGATGAAAGCATAATAGGCTTCATCAAAGGAGGGATGAAAATTCGACACAGTTATCAGCTTTACAAGAATTGCCAGGCCATGGAAAAAGTCACAAAGGATGAGGAAAAACAGAGAAGCACACACATTCATTTTAGGGGTGGGGTCAGCATGGGAATTGGATCATTCAATCTGATGCTGTCTCTGCTTCCATCCAGAGTCCTTAGACTGATGGAGTTTTTGGGCTTCTCTGGAGACAGGGAAGTGGGTTTGTCAGAGTTGAGAGAGGGAGCAACTACCAACAACCTGCGCTCCATCCTCAGCACCCTCACTCTGCTGATGTTTCAGCTCTACATCACAGTGATACTTGGGACTGCTGATGTAAACCTAAGTGAATGTGAGGCTCTGCTGAAACCCTACTGTGAAAAGTTTCCTAATGGGGCTTTAATGCATTTCTTCAGTGCAAGGATTGCTGTGCTCAAAGGAAACTTCACATTTGCCCAAGAGAAGTTCTTGGCATGTATTGCATCGCAGGAAGAGTGGCGTCAGATTCACCACCTGTGCTACTGGGAGCTGATGTGGGTCTACTCCTTTGAACAAAACTGGTTTGAGGCCTATCGATACGCCAACTTGCTCAGCAAGGAGAACAAGTGGTCCCAGGCAGTCTATGTATTTCAGAAAGCTGCCATCTTGAGCATGATGCCAGAGGAAGAAGTGAACAAGTTCAATGAAAATGTGGTGGAATTATTCAGGCAGGTGGAGGGCCTCAGGCTGAGATTTGCTGGGAAGTCGATTCCAACGGAGAAGTTTGCAGCGAAGAAGGCCCAGAGATACTCCTCTTCCACCCCCGTGAAACTTGTCGTTCCTGCTTTGGAAATGATGTATGTGTGGAATGGCTTCACAATAGTAGGCAAAAAACCCAAGCTGACTGAAGGAATCCTGTCAACTTTAGAGAAAGCAGAAGAGCAGCTCAGAAATGATCCAAACCCATCAGAGTACCACACAGGCGATGAGTGCGTTGTCCAGCTGCTGAAGGGTCTGTGCTTAAGAAGTCTGGGACGGCTGGTCCAGGCTGAGATCTGCTTCAATCATGTCATCTCCAGTGATGGTGGCATCAAGCATGATAGCTATCTGGTGCCCTTTACCATGTATGAGCTTGGCCTATTGCACAAGCAGAAAGGTGAAATCAACAAGGCCATTGCTGTAATGGAAAATGTCATGACGAACTACAAGGACTACAACATGGAGTCAAGGCTGCATTTCCGCATCCATGCAGCACTCAACACCATGGGCTCCTTTGCAGCCAAACTTCCACCATCATGCACGGCAGCTTAA---------------------------------------------------------

>AbY3.ENSMZET00005027059.1

ATGCATCAAATGGAGGGTGAAGACAATTCACTACAACAGGTGGGTTTGAAGCAGCCCAATGAGGATGCAATATTGCCTGAAGAATTGCCTAAAATGGCTTTGGAGACAGCATTGAAAGAATGCTTCACTGCCCTCAATTTCTTCCTGAACAACAGGTTTGCCGATGCACTGGCTCTTTTAAAACCCTGGAAGAGTGAAAGCATGTACCATGCGATGGGCTACAGTAGCATTCTGGTGATGCAGGCCGCTGTGACTTTTGATGCAAAGGACATGGATGCTGCCATGACATCACTGAGAGAAGCCTTGCAGGTCTGCCAGAGATTTCGGAAGAAAACGGGACTAATGGAAAGCTTGGCTAACCTTTGGTACAGACAACCAGCTGACAATCTGACAGAAGAAGAGATGCATGCAGAGCTGTGCTATGCTGAAGTCCTGCTGCAGAAAGCTGCCCTCACGTTCTTGGATGAAAGCATAATAGGCTTCATCAAAGGAGGGATGAAAATTCGACACAGTTATCAGCTTTACAAGAATTGCCAGGCCATGGAAAAAGTCACAAAGGATGAGGAAAAACAGAGAAGCACACACATTCATTTTAGGGGTGGGGTCAGCATGGGAATTGGATCATTCAATCTGATRCTGTCTCTGCTTCCATCCAGAGTCCTTAGACTGATGGAGTTTTTGGGCTTCTCTGGAGACAGGGAAGTGGGTTTGTCAGAGTTGAGAGAGGGAGCAACTACCAACAACCTGCGCTCCATCCTCAGCACCCTCACTCTGCTGATGTTTCAGCTCTACATCACAGTGATACTTGGGACTGCTGATGTAAACCTAAGTGAATGTGAGGCTCTGCTGAAACCCTACTGTGAAAAGTTTCCTAATGGGGCTTTAATGCATTTCTTCAGTGCAAGGATTGCTGTGCTCAAAGGAAACTTCACATTTGCCCAAGAGAAGTTCTTGGCATGTATTGCATCGCAGGAAGAGTGGCGTCAGATTCACCACCTGTGCTACTGGGAGCTGATGTGGGTCTACTCCTTTGAACAAAACTGGTTTGAGGCCTATCGATACGCCAACTTGCTCAGCAAGGAGAACAAGTGGTCCCAGGCAGTCTATGTATTTCAGAAAGCTGCCATCTTGAGCATGATGCCAGAGGAAGAAGTGAACAAGTTCAATGAAAATGTGGTGGAATTATTCAGGCAGGTGGAGGGCCTCAGGCTGAGATTTGCTGGGAAGTCGATTCCAACGGAGAAGTTTGCAGCGAAGAAGGCCCAGAGATACTCCTCTTCCACCCCCGTGAAACTTGTCGTTCCTGCTTTGGAAATGATGTATGTGTGGAATGGCTTCACAATAGTAGGCAAAAAACCCRAGCTGACTGAAGGAATCCTGTCAACTTTAGAGAAAGCAGAAGAGCAGCTCAGAAATGATCCAAACCCATCAGAGTACCACACAGGCGATGAGTGCGTTGTCCAGCTGCTGAAGGGTCTGTGCTTAAGAAGTCTGGGACGGCTGGTCCAGGCTGAGATCTGCTTCAATCATGTCATCTCCAGTGATGGTGGCATCAAGCATGATAGCTATCTGGTGCCCTTTACCATGTATGAGCTTGGCCTATTGCACAAGCAGAAAGGTGAAATCAACAAGGCCATTGCTGTAATGGAAAATGTCATGACGAACTACAAGGACTACAACATGGAGTCAAGGCTGCATTTCCGCATCCATGCAGCACTCAACACCATGGGCTCCTTTGCAGCCAAACTTCCACCATCATGCACGGCAGCTTAA---------------------------------------------------------

>AbY4.ENSMZET00005027059.1

ATGCATCAAATGGAGGGTGAAGACAATTCACTACAACAGGTGGGTTTGAAGCAGCCCAATGAGGATGCAATATTGCCTGAAGAATTGCCTAAAATGGCTTTGGAGACAGCATTGAAAGAATGCTTCACTGCCCTCAATTTCTTCCTGAACAACAGGTTTGCCGATGCACTGGCTCTTTTAAAACCCTGGAAGAGTGAAAGCATGTACCATGCGATGGGCTACAGTAGCATTCTGGTGATGCAGGCCGCTGTGACTTTTGATGCAAAGGACATGGATGCTGCCATGACATCACTGAGAGAAGCCTTGCAGGTCTGCCAGAGATTTCGGAAGAAAACGGGACTAATGGAAAGCTTGGCTAACCTTTGGTACAGACAACCAGCTGACAATCTGACAGAAGAAGAGATGCATGCAGAGCTGTGCTATGCTGAAGTCCTGCTGCAGAAAGCTGCCCTCACGTTCTTGGATGAAAGCATAATAGGCTTCATCAAAGGAGGGATGAAAATTCGACACAGTTATCAGCTTTACAAGAATTGCCAGGCCATGGAAAAAGTCACAAAGGATGAGGAAAAACAGAGAAGCACACACATTCATTTTAGGGGTGGGGTCAGCATGGGAATTGGATCATTCAATCTGATGCTGTCTCTGCTTCCATCCAGAGTCCTTAGACTGATGGAGTTTTTGGGCTTCTCTGGAGACAGGGAAGTGGGTTTGTCAGAGTTGAGAGAGGGAGCAACTACCAACAACCTGCGCTCCATCCTCAGCACCCTCACTCTGCTGATGTTTCAGCTCTACATCACAGTGATACTTGGGACTGCTGATGTAAACCTAAGTGAATGTGAGGCTCTGCTGAAACCCTACTGTGAAAAGTTTCCTAATGGGGCTTTAATGCATTTCTTCAGTGCAAGGATTGCTGTGCTCAAAGGAAACTTCACATTTGCCCAAGAGAAGTTCTTGGCATGTATTGCATCGCAGGAAGAGTGGCGTCAGATTCACCACCTGTGCTACTGGGAGCTGATGTGGGTCTACTCCTTTGAACAAAACTGGTTTGAGGCCTATCGATACGCCAACTTGCTCAGCAAGGAGAACAAGTGGTCCCAGGCAGTCTATGTATTTCAGAAAGCTGCCATCTTGAGCATGATGCCAGAGGAAGAAGTGAACAAGTTCAATGAAAATGTGGTGGAATTATTCAGGCAGGTGGAGGGCCTCAGGCTGAGATTTGCTGGGAAGTCGATTCCAACGGAGAAGTTTGCAGCGAAGAAGGCCCAGAGATACTCCTCTTCCACCCCCGTGAAACTTGTCGTTCCTGCTTTGGAAATGATGTATGTGTGGAATGGCTTCACAATAGTAGGCAAAAAACCCAAGCTGACTGAAGGAATCCTGTCAACTTTAGAGAAAGCAGAAGAGCAGCTCAGAAATGATCCAAACCCATCAGAGTACCACACAGGCGATGAGTGCGTTGTCCAGCTGCTGAAGGGTCTGTGCTTAAGAAGTCTGGGACGGCTGGTCCAGGCTGAGATCTGCTTCAATCATGTCATCTCCAGTGATGGTGGCATCAAGCATGATAGCTATCTGGTGCCCTTTACCATGTATGAGCTTGGCCTATTGCACAAGCAGAAAGGTGAAATCAACAAGGCCATTGCTGTAATGGAAAATGTCATGACGAACTACAAGGACTACAACATGGAGTCAAGGCTGCATTTCCGCATCCATGCAGCACTCAACACCATGGGCTCCTTTGCAGCCAAACTTCCACCATCATGCACGGCAGCTTAA---------------------------------------------------------

>AbY6.ENSMZET00005027059.1

ATGCATCAAATGGAGGGTGAAGACAATTCACTACAACAGGTGGGTTTGAAGCAGCCCAATGAGGATGCAATATTGCCTGAAGAATTGCCTAAAATGGCTTTGGAGACAGCATTGAAAGAATGCTTCACTGCCCTCAATTTCTTCCTGAACAACAGGTTTGCCGATGCACTGGCTCTTTTAAAACCCTGGAAGAGTGAAAGCATGTACCATGCGATGGGCTACAGTAGCATTCTGGTGATGCAGGCCGCTGTGACTTTTGATGCAAAGGACATGGATGCTGCCATGACATCACTGAGAGAAGCCTTGCAGGTCTGCCAGAGATTTCGGAAGAAAACGGGACTAATGGAAAGCTTGGCTAACCTTTGGTACAGACAACCAGCTGACAATCTGACAGAAGAAGAGATGCATGCAGAGCTGTGCTATGCTGAAGTCCTGCTGCAGAAAGCTGCCCTCACGTTCTTGGATGAAAGCATAATAGGCTTCATCAAAGGAGGGATGAAAATTCGACACAGTTATCAGCTTTACAAGAATTGCCAGGCCATGGAAAAAGTCACAAAGGATGAGGAAAAACAGAGAAGCACACACATTCATTTTAGGGGTGGGGTCAGCATGGGAATTGGATCATTCAATCTGATRCTGTCTCTGCTTCCATCCAGAGTCCTTAGACTGATGGAGTTTTTGGGCTTCTCTGGAGACAGGGAAGTGGGTTTGTCAGAGTTGAGAGAGGGAGCAACTACCAACAACCTGCGCTCCATCCTCAGCACCCTCACTCTGCTGATGTTTCAGCTCTACATCACAGTGATACTTGGGACTGCTGATGTAAACCTAAGTGAATGTGAGGCTCTGCTGAAACCCTACTGTGAAAAGTTTCCTAATGGGGCTTTAATGCATTTCTTCAGTGCAAGGATTGCTGTGCTCAAAGGAAACTTCACATTTGCCCAAGAGAAGTTCTTGGCATGTATTGCATCGCAGGAAGAGTGGCGTCAGATTCACCACCTGTGCTACTGGGAGCTGATGTGGGTCTACTCCTTTGAACAAAACTGGTTTGAGGCCTATCGATACGCCAACTTGCTCAGCAAGGAGAACAAGTGGTCCCAGGCAGTCTATGTATTTCAGAAAGCTGCCATCTTGAGCATGATGCCAGAGGAAGAAGTGAACAAGTTCAATGAAAATGTGGTGGAATTATTCAGGCAGGTGGAGGGCCTCAGGCTGAGATTTGCTGGGAAGTCGATTCCAACGGAGAAGTTTGCAGCGAAGAAGGCCCAGAGATACTCCTCTTCCACCCCCGTGAAACTTGTCGTTCCTGCTTTGGAAATGATGTATGTGTGGAATGGCTTCACAATAGTAGGCAAAAAACCCRAGCTGACTGAAGGAATCCTGTCAACTTTAGAGAAAGCAGAAGAGCAGCTCAGAAATGATCCAAACCCATCAGAGTACCACACAGGCGATGAGTGCGTTGTCCAGCTGCTGAAGGGTCTGTGCTTAAGAAGTCTGGGACGGCTGGTCCAGGCTGAGATCTGCTTCAATCATGTCATCTCCAGTGATGGTGGCATCAAGCATGATAGCTATCTGGTGCCCTTTACCATGTATGAGCTTGGCCTATTGCACAAGCAGAAAGGTGAAATCAACAAGGCCATTGCTGTAATGGAAAATGTCATGACGAACTACAAGGACTACAACATGGAGTCAAGGCTGCATTTCCGCATCCATGCAGCACTCAACACCATGGGCTCCTTTGCAGCCAAACTTCCACCATCATGCACGGCAGCTTAA---------------------------------------------------------

>TbR1.TM1 T0000032630-R1

---------ATGGAGGGTGAAGACAATTCACTACAACAGGTGGGTTTGAAGCAGCCCAATGAGGATGCAATATTGCCTGAAGAATTGCCTAAAATGGGTTTGGAGACAGCATTGAAAGAATGCTTCACTGCCCTCAATTTCTTCCTGAACAACAGGTTTGCCGATGCATTGGCTCTTTTAAAACCCTGGAAGAGTGAAAGTATGTACCATGCGATGGGCTACAGTAGCATTCTGGTGATGCAGGCCGCCATGACTTTTGATGCAAAGGACATGGATGCTGCCATGACATCACTGAGAGAAGCCTTGCAGGTCTGCCAGAGATTTCGGAAGAAAACGGGACTAATGGAAAGCTTGGCTAACCTTTGGTACAGACAACCAGCTGACAATCTGACAGAAGAAGAGATGCATGCAGAGCTGTGCTATGCTGAAGTCCTGCTGCAGAAAGCTGCCCTCACGTTCTTGGATGAAAGCATAATAGGCTTCATCAAAGGAGGGATGAAAATTCGACACAGTTATCAGCTTTACAAGGATTGCCAGGCCATGGAAAAAGTCACAAAGGATGAGGAAAAACAGAGAAGCACACACATTCATTTTAGGGGTGGGGTCAGCATGGGAATTGGATCATTCAATCTGATGCTGTCTCTGCTTCCGTCCAGAGTCCTTAGACTGATGGAGTTTTTGGGCTTCTCTGGAGACAGGGAAGTGGGTTTGTCAGAGTTGAGAGAGGGAGCAACTACCAACAACCTGCGCTCCATCCTCAGCACCCTTACTCTGCTGATGTTTCAGCTCTACATCACAGTGATACTTGGGACTGCTGATGTAAACCTAAGTGAATGTGAGGCTCTGCTGAAACCCTACTGTGAAAAGTTTCCTAATGGGGCTTTAATGCATTTCTTCAGTGCAAGGATTGCTGTGCTCAAAGGAAACTTCACATTTGCCCAAGAGAAGTTCTTGGCATGTATTGCATCGCAGGAAGAGTGGCGTCAGATTCACCACCTGTGCTACTGGGAGCTGATGTGGGTCTACTCCTTTGAACAAAACTGGTTTGAGGCCTATCGATACGCCAACCTCCTCAGCAAGGAGAACAAGTGGTCCCAGGCAGTCTATGTATTTCAGAAAGCTGCCATCTTGAGCATGATGCCAGAGGAAGAAGTGAACAAGCTCAATGAAAATGTGGTGGATTTATTCAGGCAGGTGGAGGGCCTCAGGCTGAGATTTGCTGGGAAGTCGATTCCAACGGAGAAGTTTGCAGCGAAGAAGGCCCAGAGATACTCCTCTTCCACCCCCGTGAAACTTGTCGTTCCTGCTTTGGAAATGATGTATGTGTGGAATGGCTTCACAATAGTAGGCAAAAGACCTGAGCTGACTGAAGGAATCCTGTCAACTTTAGAGAAAGCAGAAGAGCAGCTCAGAAATGATCCAAACCCATCAGAGTACCACACGGGCGATGAGTGCATTGTCCAGCTGCTGAAGGGTCTGTGCTTAAGAAGTCTGGGACGGCTGGTCCAGGCTGAGATCTGCTTCAATTATGTAATCTCCAGTGATGGTGGCATCAAGCATGATAGCTATCTGGTGCCCTTTACCATGTATGAGCTTGGCCTATTGCACAAGCAGAAAGGTGAAATCAACAAGGCCATTGCTGTAATGGAAAATGTCATGACTAACTACAAGGACTACAACATGGAGTCAAGGCTGCATTTCCGCATCCATGCAGCACTCAACACCATAGGCTCCTTTGCAGCCAAACTTCCACCATCACGCACGGCAGCTTAAGAGATCCCATTACATGAAACTTGACAGTGTTTACTGTT-------------------

>TbR2.TM1 T0000032630-R1

---------ATGGAGGGTGAAGACAATTCACTACAACAGGTGGGTTTGAAGCAGCCCAATGAGGATGCAATATTGCCTGAAGAATTGCCTAAAATGGGTTTGGAGACAGCATTGAAAGAATGCTTCACTGCCCTCAATTTCTTCCTGAACAACAGGTTTGCCGATGCATTGGCTCTTTTAAAACCCTGGAAGAGTGAAAGTATGTACCATGCGATGGGCTACAGTAGCATTCTGGTGATGCAGGCCGCCATGACTTTTGATGCAAAGGACATGGATGCTGCCATGACATCACTGAGAGAAGCCTTGCAGGTCTGCCAGAGATTTCGGAAGAAAACGGGACTAATGGAAAGCTTGGCTAACCTTTGGTACAGACAACCAGCTGACAATCTGACAGAAGAAGAGATGCATGCAGAGCTGTGCTATGCTGAAGTCCTGCTGCAGAAAGCTGCCCTCACGTTCTTGGATGAAAGCATAATAGGCTTCATCAAAGGAGGGATGAAAATTCGACACAGTTATCAGCTTTACAAGGATTGCCAGGCCATGGAAAAAGTCACAAAGGATGAGGAAAAACAGAGCAGCACACACATTCATTTTAGGGGTGGGGTCAGCATGGGAATTGGATCATTCAATCTGATGCTGTCTCTGCTTCCGTCCAGAGTCCTTAGACTGATGGAGTTTTTGGGCTTCTCTGGAGACAGGGAAGTGGGTTTGTCAGAGTTGAGAGAGGGAGCAACTACCAACAACCTGCGCTCCATCCTCAGCACCCTYACTCTGCTGATGTTTCAGCTCTACATCACAGTGATACTTGGGACTGCTGATGTAAACCTAAGTGAATGTGAGGCTCTGCTGAAACCCTACTGTGAAAAGTTTCCTAATGGGGCTTTAATGCATTTCTTCAGTGCAAGGATTGCTGTGCTCAAAGGAAACTTCACATTTGCCCAAGAGAAGTTCTTGGCATGTATTGCATCGCAGGAAGAGTGGCGTCAGATTCACCACCTGTGCTACTGGGAGCTGATGTGGGTCTACTCCTTTGAACAAAACTGGTTTGAGGCCTATCGATACGCCAACCTCCTCAGCAAGGAGAACAAGTGGTCCCAGGCAGTCTATGTATTTCAGAAAGCTGCCATCTTGAGCATGATGCCAGAGGAAGAAGTGAACAAGCTCAATGAAAATGTGGTGGATTTATTCAGGCAGGTGGAGGGCCTCAGGCTGAGATTTGCTGGGAAGTCGATTCCAACGGAGAAGTTTGCAGCGAAGAAGGCCCAGAGATACTCCTCTTCCACCCCCGTGAAACTTGTCGTTCCTGCTTTGGAAATGATGTATGTGTGGAATGGCTTCACAATAGTAGGCAAAAGACCTGAGCTGACTGAAGGAATCCTGTCAACTTTAGAGAAAGCAGAAGAGCAGCTCAGAAATGATCCAAACCCATCAGAGTACCACACGGGCGATGAGTGCATTGTCCAGCTGCTGAAGGGTCTGTGCTTAAGAAGTCTGGGACGGCTGGTCCAGGCTGAGATCTGCTTCAATTATGTAATCTCCAGTGATGGTGGCATCAAGCATGATAGCTATCTGGTGCCCTTTACCATGTATGAGCTTGGCCTATTGCACAAGCAGAAAGGTGAAATCAACAAGGCCATTGCTGTAATGGAAAATGTCATGACTAACTACAAGGACTACAACATGGAGTCAAGGCTGCATTTCCGCATCCATGCAGCACTCAACACCATAGGCTCCTTTGCAGCCAAACTTCCACCATCACGCACGGCAGCTTAAGAGATCCCATTACATGAAACTTGACAGTGTTTACTGTTTCATTG-------------

>TbR3.TM1 T0000032630-R1

---------ATGGAGGGTGAAGACAATTCACTACAACAGGTGGGTTTGAAGCAGCCCAATGAGGATGCAATATTGCCTGAAGAATTGCCTAAAATGGGTTTGGAGACAGCATTGAAAGAATGCTTCACTGCCCTCAATTTCTTCCTGAACAACAGGTTTGCCGATGCATTGGCTCTTTTAAAACCCTGGAAGAGTGAAAGTATGTACCATGCGATGGGCTACAGTAGCATTCTGGTGATGCAGGCCGCCATGACTTTTGATGCAAAGGACATGGATGCTGCCATGACATCACTGAGAGAAGCCTTGCAGGTCTGCCAGAGATTTCGGAAGAAAACGGGACTAATGGAAAGCTTGGCTAACCTTTGGTACAGACAACCAGCTGACAATCTGACAGAAGAAGAGATGCATGCAGAGCTGTGCTATGCTGAAGTCCTGCTGCAGAAAGCTGCCCTCACGTTCTTGGATGAAAGCATAATAGGCTTCATCAAAGGAGGGATGAAAATTCGACACAGTTATCAGCTTTACAAGGATTGCCAGGCCATGGAAAAAGTCACAAAGGATGAGGAAAAACAGAGMAGCRCACACATTCATTTTAGGGGTGGGGTCAGCATGGGAATTGGATCATTCAATCTGATGCTGTCTCTGCTTCCGTCCAGAGTCCTTAGACTGATGGAGTTTTTGGGCTTCTCTGGAGACAGGGAAGTGGGTTTGTCAGAGTTGAGAGAGGGAGCAACTACCAACAACCTGCGCTCCATCCTCAGCACCCTTACTCTGCTGATGTTTCAGCTCTACATCACAGTGATACTTGGGACTGCTGATGTAAACCTAAGTGAATGTGAGGCTCTGCTGAAACCCTACTGTGAAAAGTTTCCTAATGGGGCTTTAATGCATTTCTTCAGTGCAAGGATTGCTGTGCTCAAAGGAAACTTCACATTTGCCCAAGAGAAGTTCTTGGCATGTATTGCATCGCAGGAAGAGTGGCGTCAGATTCACCACCTGTGCTACTGGGAGCTGATGTGGGTCTACTCCTTTGAACAAAACTGGTTTGAGGCCTATCGATACGCCAACCTCCTCAGCAAGGAGAACAAGTGGTCCCAGGCAGTCTATGTATTTCAGAAAGCTGCCATCTTGAGCATGATGCCAGAGGAAGAAGTGAACAAGCTCAATGAAAATGTGGTGGATTTATTCAGGCAGGTGGAGGGCCTCAGGCTGAGATTTGCTGGGAAGTCGATTCCAACGGAGAAGTTTGCAGCGAAGAAGGCCCAGAGATACTCCTCTTCCACCCCCGTGAAACTTGTCGTTCCTGCTTTGGAAATGATGTATGTGTGGAATGGCTTCACAATAGTAGGCAAAAGACCTGAGCTGACTGAAGGAATCCTGTCAACTTTAGAGAAAGCAGAAGAGCAGCTCAGAAATGATCCAAACCCATCAGAGTACCACACGGGCGATGAGTGCATTGTCCAGCTGCTGAAGGGTCTGTGCTTAAGAAGTCTGGGACGGCTGGTCCAGGCTGAGATCTGCTTCAATTATGTAATCTCCAGTGATGGTGGCATCAAGCATGATAGCTATCTGGTGCCCTTTACCATGTATGAGCTTGGCCTATTGCACAAGCAGAAAGGTGAAATCAACAAGGCCATTGCTGTAATGGAAAATGTCATGACTAACTACAAGGACTACAACATGGAGTCAAGGCTGCATTTCCGCATCCATGCAGCACTCAACACCATAGGCTCCTTTGCAGCCAAACTTCCACCATCACGCACGGCAGCTTAAGAGATCCCATTACATGAAACTTGACAGTGTTTACTGTT-------------------

>TbR4.TM1 T0000032630-R1

---------ATGGAGGGTGAAGACAATTCACTACAACAGGTGGGTTTGAAGCAGCCCAATGAGGATGCAATATTGCCTGAAGAATTGCCTAAAATGGGTTTGGAGACAGCATTGAAAGAATGCTTCACTGCCCTCAATTTCTTCCTGAACAACAGGTTTGCCGATGCATTGGCTCTTTTAAAACCCTGGAAGAGTGAAAGTATGTACCATGCGATGGGCTACAGTAGCATTCTGGTGATGCAGGCCGCCATGACTTTTGATGCAAAGGACATGGATGCTGCCATGACATCACTGAGAGAAGCCTTGCAGGTCTGCCAGAGATTTCGGAAGAAAACGGGACTAATGGAAAGCTTGGCTAACCTTTGGTACAGACAACCAGCTGACAATCTGACAGAAGAAGAGATGCATGCAGAGCTRTGCTATGCTGAAGTCCTGCTGCAGAAAGCTGCCCTCACRTTCTTGGATGAAAGCATAATAGGCTTCATCAAAGGAGGGATGAAAATTCGACACAGTTATCAGCTTTACAAGGATTGCCAGGCCATGGAAAAAGTCACAAAGGATGAGGAAAAACAGAGCAGCACACACATTCATTTTAGGGGTGGGGTCAGCATGGGAATTGGATCATTCAATCTGATGCTGTCTCTGCTTCCGTCCAGAGTCCTTAGACTGATGGAGTTTTTGGGCTTCTCTGGAGACAGGGAAGTGGGTTTGTCAGAGTTGAGAGAGGGAGCAACTACCAACAACCTGCGCTCCATCCTCAGCACCCTTACTCTGCTGATGTTTCAGCTCTACATCACAGTGATACTTGGGACTGCTGATGTAAACCTAAGTGAATGTGAGGCTCTGCTGAAACCCTACTGTGAAAAGTTTCCTAATGGGGCTTTAATGCATTTCTTCAGTGCAAGGATTGCTGTGCTCAAAGGAAACTTCACATTTGCCCAAGAGAAGTTCTTGGCATGTATTGCATCGCAGGAAGAGTGGCGTCAGATTCACCACCTGTGCTACTGGGAGCTGATGTGGGTCTACTCCTTTGAACAAAACTGGTTTGAGGCCTATCGATACGCCAACCTCCTCAGCAAGGAGAACAAGTGGTCCCAGGCAGTCTATGTATTTCAGAAAGCTGCCATCTTGAGCATGATGCCAGAGGAAGAAGTGAACAAGCTCAATGAAAATGTGGTGGATTTATTCAGGCAGGTGGAGGGCCTCAGGCTGAGATTTGCTGGGAAGTCGATTCCAACGGAGAAGTTTGCAGCGAAGAAGGCCCAGAGATACTCCTCTTCCACCCCCGTGAAACTTGTCGTTCCTGCTTTGGAAATGATGTATGTGTGGAATGGCTTCACAATAGTAGGCAAAAGACCTGAGCTGACTGAAGGAATCCTGTCAACTTTAGAGAAAGCAGAAGAGCAGCTCAGAAATGATCCAAACCCATCAGAGTACCACACGGGCGATGAGTGCATTGTCCAGCTGCTGAAGGGTCTGTGCTTAAGAAGTCTGGGACGGCTGGTCCAGGCTGAGATCTGCTTCAATTATGTAATCTCCAGTGATGGTGGCATCAAGCATGATAGCTATCTGGTGCCCTTTACCATGTATGAGCTTGGCCTATTGCACAAGCAGAAAGGTGAAATCAACAAGGCCATTGCTGTAATGGAAAATGTCATGACTAACTACAAGGACTACAACATGGAGTCAAGGCTGCATTTCCGCATCCATGCAGCACTCAACACCATAGGCTCCTTTGCAGCCAAACTTCCACCATCACGCACGGCAGCTTAAGAGATCCCATTACATGAAACTTGACAGTGTTTACTGTTTCATTG-------------

>TbR5.TM1 T0000032630-R1

---------ATGGAGGGTGAAGACAATTCACTACAACAGGTGGGTTTGAAGCAGCCCAATGAGGATGCAATATTGCCTGAAGAATTGCCTAAAATGGGTTTGGAGACAGCATTGAAAGAATGCTTCACTGCCCTCAATTTCTTCCTGAACAACAGGTTTGCCGATGCATTGGCTCTTTTAAAACCCTGGAAGAGTGAAAGTATGTACCATGCGATGGGCTACAGTAGCATTCTGGTGATGCAGGCCGCCATGACTTTTGATGCAAAGGACATGGATGCTGCCATGACATCACTGAGAGAAGCCTTGCAGGTCTGCCAGAGATTTCGGAAGAAAACGGGACTAATGGAAAGCTTGGCTAACCTTTGGTACAGACAACCAGCTGACAATCTGACAGAAGAAGAGATGCATGCAGAGCTGTGCTATGCTGAAGTCCTGCTGCAGAAAGCTGCCCTCACGTTCTTGGATGAAAGCATAATAGGCTTCATCAAAGGAGGGATGAAAATTCGACACAGTTATCAGCTTTACAAGGATTGCCAGGCCATGGAAAAAGTCACAAAGGATGAGGAAAAACAGAGCAGCACACACATTCATTTTAGGGGTGGGGTCAGCATGGGAATTGGATCATTCAATCTGATGCTGTCTCTGCTTCCGTCCAGAGTCCTTAGACTGATGGAGTTTTTGGGCTTCTCTGGAGACAGGGAAGTGGGTTTGTCAGAGTTGAGAGAGGGAGCAACTACCAACAACCTGCGCTCCATCCTCAGCACCCTCACTCTGCTGATGTTTCAGCTCTACATCACAGTGATACTTGGGACTGCTGATGTAAACCTAAGTGAATGTGAGGCTCTGCTGAAACCCTACTGTGAAAAGTTTCCTAATGGGGCTTTAATGCATTTCTTCAGTGCAAGGATTGCTGTGCTCAAAGGAAACTTCACATTTGCCCAAGAGAAGTTCTTGGCATGTATTGCATCGCAGGAAGAGTGGCGTCAGATTCACCACCTGTGCTACTGGGAGCTGATGTGGGTCTACTCCTTTGAACAAAACTGGTTTGAGGCCTATCGATACGCCAACCTCCTCAGCAAGGAGAACAAGTGGTCCCAGGCAGTCTATGTATTTCAGAAAGCTGCCATCTTGAGCATGATGCCAGAGGAAGAAGTGAACAAGCTCAATGAAAATGTGGTGGATTTATTCAGGCAGGTGGAGGGCCTCAGGCTGAGATTTGCTGGGAAGTCGATTCCAACGGAGAAGTTTGCAGCGAAGAAGGCCCAGAGATACTCCTCTTCCACCCCCGTGAAACTTGTCGTTCCTGCTTTGGAAATGATGTATGTGTGGAATGGCTTCACAATAGTAGGCAAAAGACCTGAGCTGACTGAAGGAATCCTGTCAACTTTAGAGAAAGCAGAAGAGCAGCTCAGAAATGATCCAAACCCATCAGAGTACCACACGGGCGATGAGTGCATTGTCCAGCTGCTGAAGGGTCTGTGCTTAAGAAGTCTGGGACGGCTGGTCCAGGCTGAGATCTGCTTCAATTATGTAATCTCCAGTGATGGTGGCATCAAGCATGATAGCTATCTGGTGCCCTTTACCATGTATGAGCTTGGCCTATTGCACAAGCAGAAAGGTGAAATCAACAAGGCCATTGCTGTAATGGAAAATGTCATGACTAACTACAAGGACTACAACATGGAGTCAAGGCTGCATTTCCGCATCCATGCAGCACTCAACACCATAGGCTCCTTTGCAGCCAAACTTCCACCATCACGCACGGCAGCTTAAGAGATCCCATTACATGAAACTTGACAGTGTTTACTGTT-------------------

>TbR6.TM1 T0000032630-R1

---------ATGGAGGGTGAAGACAATTCACTACAACAGGTGGGTTTGAAGCAGCCCAATGAGGATGCAATATTGCCTGAAGAATTGCCTAAAATGGGTTTGGAGACAGCATTGAAAGAATGCTTCACTGCCCTCAATTTCTTCCTGAACAACAGGTTTGCCGATGCATTGGCTCTTTTAAAACCCTGGAAGAGTGAAAGTATGTACCATGCGATGGGCTACAGTAGCATTCTGGTGATGCAGGCCGCCATGACTTTTGATGCAAAGGACATGGATGCTGCCATGACATCACTGAGAGAAGCCTTGCAGGTCTGCCAGAGATTTCGGAAGAAAACGGGACTAATGGAAAGCTTGGCTAACCTTTGGTACAGACAACCAGCTGACAATCTGACAGAAGAAGAGATGCATGCAGAGCTGTGCTATGCTGAAGTCCTGCTGCAGAAAGCTGCCCTCACGTTCTTGGATGAAAGCATAATAGGCTTCATCAAAGGAGGGATGAAAATTCGACACAGTTATCAGCTTTACAAGGATTGCCAGGCCATGGAAAAAGTCACAAAGGATGAGGAAAAACAGAGAAGCACACACATTCATTTTAGGGGTGGGGTCAGCATGGGAATTGGATCATTCAATCTGATGCTGTCTCTGCTTCCGTCCAGAGTCCTTAGACTGATGGAGTTTTTGGGCTTCTCTGGAGACAGGGAAGTGGGTTTGTCAGAGTTGAGAGAGGGAGCAACTACCAACAACCTGCGCTCCATCCTCAGCACCCTCACTCTGCTGATGTTTCAGCTCTACATCACAGTGATACTTGGGACTGCTGATGTAAACCTAAGTGAATGTGAGGCTCTGCTGAAACCCTACTGTGAAAAGTTTCCTAATGGGGCTTTAATGCATTTCTTCAGTGCAAGGATTGCTGTGCTCAAAGGAAACTTCACATTTGCCCAAGAGAAGTTCTTGGCATGTATTGCATCGCAGGAAGAGTGGCGTCAGATTCACCACCTGTGCTACTGGGAGCTGATGTGGGTCTACTCCTTTGAACAAAACTGGTTTGAGGCCTATCGATACGCCAACCTCCTCAGCAAGGAGAACAAGTGGTCCCAGGCAGTCTATGTATTTCAGAAAGCTGCCATCTTGAGCATGATGCCAGAGGAAGAAGTGAACAAGCTCAATGAAAATGTGGTGGATTTATTCAGGCAGGTGGAGGGCCTCAGGCTGAGATTTGCTGGGAAGTCGATTCCAACGGAGAAGTTTGCAGCGAAGAAGGCCCAGAGATACTCCTCTTCCACCCCCGTGAAACTTGTCGTTCCTGCTTTGGAAATGATGTATGTGTGGAATGGCTTCACAATAGTAGGCAAAAGACCTGAGCTGACTGAAGGAATCCTGTCAACTTTAGAGAAAGCAGAAGAGCAGCTCAGAAATGATCCAAACCCATCAGAGTACCACACGGGCGATGAGTGCATTGTCCAGCTGCTGAAGGGTCTGTGCTTAAGAAGTCTGGGACGGCTGGTCCAGGCTGAGATCTGCTTCAATTATGTAATCTCCAGTGATGGTGGCATCAAGCATGATAGCTATCTGGTGCCCTTTACCATGTATGAGCTTGGCCTATTGCACAAGCAGAAAGGTGAAATCAACAAGGCCATTGCTGTAATGGAAAATGTCATGACTAACTACAAGGACTACAACATGGAGTCAAGGCTGCATTTCCGCATCCATGCAGCACTCAACACCATAGGCTCCTTTGCAGCCAAACTTCCACCATCACGCACGGCAGCTTAAGAGATCCCATTACATGAAACTTGACAGTGTTTACTGTT-------------------

>TbY1.TM1 T0000032630-R1

---------nnnnnnnnnnnnnnnnnnnnnnnnnnnnnnnnnnnnnnnnnnnnnnnnnnnnnnnnnACAATATTGCCTGAAGAATTGCCTAAAATGGGTTTGGAGACAGCATTGAAAGAATGCTTCACTGCCCTCAATTTCTTCCTGAACAACAGGTTTGCCGATGCATTGGCTCTTTTAAAACCCTGGAAGAGTGAAAGTATGTACCATGCGATGGGCTACAGTAGCATTCTGGTGATGCAGGCCGCCATGACTTTTGATGCAAAGGACATGGATGCTGCCATGACATCACTGAGAGAAGCCTTGCAGGTCTGCCAGnnnnnnnnnnAGAAAACGGGACTAATGGAAAGCTTGGCTAACCTTTGGTACAGACAACCAGCTGACAATCTGACAGAAGAAGAGATGCATGCAGAGCTGTGCTATGCTGAAGTCCTGCTGCAGAAAGCTGCCCTCACGTTCTTGGATGAAAGCATAATAGGCTTCATCAAAGGAGGGATGAAAATTCGACACAGTTATCAGCTTTACAAGGATTGCCnnnnnnnnnnnnnnnnnnnnnnnnnnnnnnnnnnnnnnnnnnnnnnnnnnnnnnnnnnnnnnnnnnnGGTCAGCATGGGAATTGGATCATTCAATCTGATGCTGTCTCTGCTTCCGTCCAGAGTCCTTAGACTGATGGAGTTTTTGGGCTTCTCTGGAGACAGGGAAGTGGGTTTGTCAGAGTTGAGAGAGGGAGCAACTACCAACAACCTGCGCTCCATCCTCAGCACCCTTACTCTGCTGATGTTTCAGCTCTACATCACAGTGATACTTGGGACTGCTGATGTAAACCTAAGTGAATGTGAGGCTCTGCTGAAACCCTACTGTGAnnnnnnnnnnnnnnnnnnnnnnnnnnnnnnnnnnnnnnnnnnnnnnnnnnnnnnnnAAGGAAACTTCACATTTGCCCAAGAGAAGTTCTTGGCATGTATTACATCGCAGGAAGAGTGGCGTCAGATTCACCACCTGTGCTACTGGGAGCTGATGTGGGTCTACTCCTTTGAACAAAACTGGTTTGAGGCCTATCGATACGCCAACCTCCTCAGCAAGGAGAACAAGTGGTCCCAGGCAGTCTATGTATTTCAGAAAGCTGCCATCTTGAGCATGATGCCAGAGGAAGAAGTGAACAAGCTCAATGAAAATGTGGTGGATTTATTCAGGCAGGTGGAGGGCCTCAGGCTGAGATTTGCTGGGAAGTCGATTCCAACGGAGAAGTTTGCAGCGAAGAAGGCCCAGAGATACTCCTCTTCCACCCCCGTGAAACTTGTCGTTCCTGCTTTGGAAATGATGTATGTGTGGAATGGCTTCACAATAGTAGGCAAAAGACCTGAGCTGACTGAAGGAATCCTGTCAACTTTAGAGAAAGCAGAAGAGCAGCTCAGAAATGATCCAAACCCATCAGAGTACCACACGGGCGATGAGTGCGTTGTCCAGCTGCTGAAGGGTCTGTGCTTAAGAAGTCTGGGACGGCTGGTCCAGGCTGAGATCTGCTTCAATTATGTAATCTCCAGTGATGGTGGCATCAAGCATGATAGCTATCTGGTGCCCTTTACCATGTATGAGCTTGGCCTATTGCACAAGCAGAAAGGTGAAATCAACAAGGCCATTGCTGTAATGGAAAATGTCATGACTAACTACAAGGACTACAACATGGAGTCAAGGCTGCATTTCCGCATCCATGCAGCACTCAACACCATGGGCTCCTTTGCAGCCAAACTTCCACCATCACGCACGGCAGCTTAAGAGATCCCATTACATGAAACTTGACATTGTTTACTGT--------------------

>TbY2.TM1 T0000032630-R1

---------ATGGAGGGTGAAGACAATTCACTACAACAGGTGGGTTTGAAGCAGCCCAATGAGGATGCAATATTGCCTGAAGAATTGCCTAAAATGGGTTTGGAGACAGCATTGAAAGAATGTTTCACTGCCCTCAATTTCTTCCTGAACAACAGGTTTGCCGATGCATTGGCTCTTTTAAAACCCTGGAAGAGTGAAAGTATGTACCATGCGATGGGCTACAGTAGCATTCTGGTGATGCAGGCCGCCATGACTTTTGATGCAAAGGACATGGATGCTGCCATGACATCACTGAGAGAAGCCTTGCAGGTCTGCCAGAGATTTCGGAAGAAAACGGGACTAATGGAAAGCTTGGCTAACCTTTGGTACAGACAACCAGCTGACAATCTGACAGAAGAAGAGATGCATGCAGAGCTGTGCTATGCTGAAGTCCTGCTGCAGAAAGCTGCCCTCACGTTCTTGGATGAAAGCATAATAGGCTTCATCAAAGGAGGGATGAAAATTCGACACAGTTATCAGCTTTACAAGGATTGCCAGGCCATGGAAAAAGTCACAAAGGATGAGGAAAAACAGAGAAGCGCACACATTCATTTTAGGGGTGGGGTCAGCATGGGAATTGGATCATTCAATCTGATGCTGTCTCTGCTTCCGTCCAGAGTCCTTAGACTGATGGAGTTTTTGGGCTTCTCTGGAGACAGGGAAGTGGGTTTGTCAGAGTTGAGAGAGGGAGCAACTACCAACAACCTGCGCTCCATCCTCAGCACCCTCACTCTGCTGATGTTTCAGCTCTACATCACAGTGATACTTGGGACTGCTGATGTAAACCTAAGTGAATGTGAGGCTCTGCTGAAACCCTACTGTGAAAAGTTTCCTAATGGGGCTTTAATGCATTTCTTCAGTGCAAGGATTGCTGTGCTCAAAGGAAACTTCACATTTGCCCAAGAGAAGTTCTTGGCATGTATTGCATCGCAGGAAGAGTGGCGTCAGATTCACCACCTGTGCTACTGGGAGCTGATGTGGGTCTACTCCTTTGAACAAAACTGGTTTGAGGCCTATCGATACGCCAACCTCCTCAGCAAGGAGAACAAGTGGTCCCAGGCAGTCTATGTATTTCAGAAAGCTGCCATCTTGAGCATGATGCCAGAGGAAGAAGTGAACAAGCTCAATGAAAATGTGGTGGATTTATTCAGGCAGGTGGAGGGCCTCAGGCTGAGATTTGCTGGGAAGTCGATTCCAACGGAGAAGTTTGCAGCGAAGAAGGCCCAGAGATACTCCTCTTCCACCCCCGTGAAACTTGTCGTTCCTGCTTTGGAAATGATGTATGTGTGGAATGGCTTCACAATAGTAGGCAAAAGACCTGAGCTGACTGAAGGAATCCTGTCAACTTTAGAGAAAGCAGAAGAGCAGCTCAGAAATGATCCAAACCCATCAGAGTACCACACGGGCGATGAGTGCATTGTCCAGCTGCTGAAGGGTCTGTGCTTAAGAAGTCTGGGACGGCTGGTCCAGGCTGAGATCTGCTTCAATTATGTAATCTCCAGTGATGGTGGCATCAAGCATGATAGCTATCTGGTGCCCTTTACCATGTATGAGCTTGGCCTATTGCACAAGCAGAAAGGTGAAATCAACAAGGCCATTGCTGTAATGGAAAATGTCATGACTAACTACAAGGACTACAACATGGAGTCAAGGCTGCATTTCCGCATCCATGCAGCACTCAACACCATAGGCTCCTTTGCAGCCAAACTTCCACCATCACGCACGGCAGCTTAAGAGATCCCATTACATGAAACTTGACAGTGTTT-------------------------

>TbY3.TM1 T0000032630-R1

---------nTGGAGGGTGAAGACAATTCACTACAACAGGTGGGTTTGAAGCAGCCCAATGAGGATGCAATATTGCCTGAAGAATTGCCTAAAATGGGTTTGGAGACAGCATTGAAAGAATGYTTCACTGCCCTCAATTTCTTCCTGAACAACAGGTTTGCCGATGCATTGGCTCTTTTAAAACCCTGGAAGAGTGAAAGTATGTACCATGCGATGGGCTACAGTAGCATTCTGGTGATGCAGGCCGCCATGACTTTTGATGCAAAGGACATGGATGCTGCCATGACATCACTGAGAGAAGCCTTGCAGGTCTGCCAGAGATTTCGGAAGAAAACGGGACTAATGGAAAGCTTGGCTAACCTTTGGTACAGACAACCAGCTGACAATCTGACAGAAGAAGAGATGCATGCAGAGCTGTGCTATGCTGAAGTCCTGCTGCAGAAAGCTGCCCTCACGTTCTTGGATGAAAGCATAATAGGCTTCATCAAAGGAGGGATGAAAATTCGACACAGTTATCAGCTTTACAAGGATTGCCAGGCCATGGAAAAAGTCACAAAGGATGAGGAAAAACAGAGAAGCACACACATTCATTTTAGGGGTGGGGTCAGCATGGGAATTGGATCATTCAATCTGATGCTGTCTCTGCTTCCGTCCAGAGTCCTTAGACTGATGGAGTTTTTGGGCTTCTCTGGAGACAGGGAAGTGGGTTTGTCAGAGTTGAGAGAGGGAGCAACTACCAACAACCTGCGCTCCATCCTCAGCACCCTTACTCTGCTGATGTTTCAGCTCTACATCACAGTGATACTTGGGACTGCTGATGTAAACCTAAGTGAATGTGAGGCTCTGCTGAAACCCTACTGTGAAAAGTTTCCTAATGGGGCTTTAATGCATTTCTTCAGTGCAAGGATTGCTGTGCTCAAAGGAAACTTCACATTTGCCCAAGAGAAGTTCTTGGCATGTATTGCATCGCAGGAAGAGTGGCGTCAGATTCACCACCTGTGCTACTGGGAGCTGATGTGGGTCTACTCCTTTGAACAAAACTGGTTTGAGGCCTATCGATACGCCAACCTCCTCAGCAAGGAGAACAAGTGGTCCCAGGCAGTCTATGTATTTCAGAAAGCTGCCATCTTGAGCATGATGCCAGAGGAAGAAGTGAACAAGCTCAATGAAAATGTGGTGGATTTATTCAGGCAGGTGGAGGGCCTCAGGCTGAGATTTGCTGGGAAGTCGATTCCAACGGAGAAGTTTGCAGCGAAGAAGGCCCAGAGATACTCCTCTTCCACCCCCGTGAAACTTGTCGTTCCTGCTTTGGAAATGATGTATGTGTGGAATGGCTTCACAATAGTAGGCAAAAGACCTGAGCTGACTGAAGGAATCCTGTCAACTTTAGAGAAAGCAGAAGAGCAGCTCAGAAATGATCCAAACCCATCAGAGTACCACACGGGCGATGAGTGCRTTGTCCAGCTGCTGAAGGGTCTGTGCTTAAGAAGTCTGGGACGGCTGGTCCAGGCTGAGATCTGCTTCAATYATGTMATCTCCAGTGATGGTGGCATCAAGCATGATAGCTATCTGGTGCCCTTTACCATGTATGAGCTTGGCCTATTGCACAAGCAGAAAGGTGAAATCAACAAGGCCATTGCTGTAATGGAAAATGTCATGACTAACTACAAGGACTACAACATGGAGTCAAGGCTGCATTTCCGCATCCATGCAGCACTCAACACCATRGGCTCCTTTGCAGCCAAACTTCCACCATCACGCACGGCAGCTTAAGAGATCCCATTACATGAAACTTGACAGTGT---------------------------

>TbY4.TM1 T0000032630-R1

---------nnnnnnnnnnnAGACAATTCACTACAACAGGTGGGTTTGAAGCAGCCCAATGAGGATGCAATATTGCCTGAAGAATTGCCTAAAATGGGTTTGGAGACAGCATTGAAAGAATGCTTCACTGCCCTCAATTTCTTCCTGAACAACAGGTTTGCCGATGCATTGGCTCTTTTAAAACCCTGGAAGAGTGAAAGTAnnnnnnnnnnnnnnnnnnACAGTAGCATTCTGGTGATGCAGGCCGCCATGACTTTTGATGCAAAGGACATGGATGCTGCCATGACATCACTGAGAGAAGCCTTGCAGGTCTGCCAGAGATTTCGGAAGAAAACGGGACTAATGGAAAGCTTGGCTAACCTTTGGTACAGACAACCAGCTGACAATCTGACAGAAGAAGAGATGCATGCAGAGCTGTGCTATGCTGAAGTCCTGCTGCAGAAAGCTGCCCTCACGTTCTTGGATGAAAGCATAATAGGCTTCATCAAAGGAGGGATGAAAATTCGACACAGTTATCAGCTTTACAAGGATTGCCAGGCCATGGAAAAAGTCACAAAGGATGAGGAAAAACAGAGAAGCGCACACATTCATTTTAGGGGTGGGGTCAGCATGGGAATTGGATCATTCAATCTGATGCTGTCTCTGCTTCCGTCCAGAGTCCTTAGACTGATGGAGTTTTTGGGCTTCTCTGGAGACAGGGAAGTGGGTTTGTCAGAGTTGAGAGAGGGAGCAACTACCAACAACCTGCGCTCCATCCTCAGCACCCTTACTCTGCTGATGTTTCAGCTCTACATCACAGTGATACTTGGGACTGCTGATGTAAACCTAAGTGAATGTGAGGCTCTGCTGAAACCCTACTGTGAAAAGTTTCCTAATGGGGCTTTAATGCATTTCTTCAGTGCAAGGATTGCTGTGCTCAAAGGAAACTTCACATTTGCCCAAGAGAAGTTCTTGGCATGTATTGCATCGCAGGAAGAGTGGCGTCAGATTCACCACCTGTGCTACTGGGAGCTGATGTGGGTCTACTCCTTTGAACAAAACTGGTTTGAGGCCTATCGATACGCCAACCTCCTCAGCAAGGAGAACAAGTGGTCCCAGGCAGTCTATGTATTTCAGAAAGCTGCCATCTTGAGCATGATGCCAGAGGAAGAAGTGAACAAGCTCAATGAAAATGTGGTGGATTTATTCAGGCAGGTGGAGGGCCTCAGGCTGAGATTTGCTGGGAAGTCGATTCCAACGGAGAAGTTTGCAGCGAAGAAGGCCCAGAGATACTCCTCTTCCACCCCCGTGAAACTTGTCGTTCCTGCTTTGGAAATGATGTATGTGTGGAATGGCTTCACAATAGTAGGCAAAAGACCTGAGCTGACTGAAGGAATCCTGTCAACTTTAGAGAAAGCAGAAGAGCAGCTCAGAAATGATCCAAACCCATCAGAGTACCACACGGGCGATGAGTGCGTTGTCCAGCTGCTGAAGGGTCTGTGCTTAAGAAGTCTGGGACGGCTGGTCCAGGCTGAGATCTGCTTCAATTATGTAATCTCCAGTGATGGTGGCATCAAGCATGATAGCTATCTGGTGCCCTTTACCATGTATGAGCTTGGCCTATTGCACAAGCAGAAAGGTGAAATCAACAAGGCCATTGCTGTAATGGAAAATGTCATGACTAACTACAAGGACTACAACATGGAGTCAAGGCTGCATTTCCGCATCCATGCAGCACTCAACACCATGGGCTCCTTTGCAGCCAAACTTCCACCATCACGCACGGCAGCTTAAGAGATCCCATTACATGAAAC-------------------------------------

>TbY5.TM1 T0000032630-R1

---------nnnnnnnnnnnnnnnnnnnnnnnnnnnnnnnnnnGTTTGAAGCAGCCCAATGAGGATGCAATATTGCCTGAAGAATTGCCTAAAATGGGTTTGGAGACAGCATTGAAAGAATGCTTCACTGCCCTCAATTTCTTCCTGAACAACAGGTTTGCCGATGCATTGGCTCTTTTAAAACCCTGGAAGAGTGAAAGTATGTACCATGCGATGGGCTACAGTAGCATTCTGGTGATGCAGGCCGCCATGACTTTTGATGCAAAGGACATGGATGCTGCCATGACATCACTGAGAGAAGCCTTGCAGGTCTGCCAGAGATTTCGGAAGAAAACGGGACTAATGGAAAGCTTGGCTAACCTTTGGTACAGACAACCAGCTGACAATCTGACAGAAGAAGAGATGCATGCAGAGCTGTGCTATGCTGAAGTCCTGCTGCAGAAAGCTGCCnnnnnnnnnnnnnnnnnnnnnnnnnnnnnnnnnnnnnnnnnnnnnnnnnnnnnnnnnnnnnnnnnnnnnnnnnnnnnnnnnnnnnnnnnnnnnnnnnnnnnnnnnnnnnnnnnnnnnnnnnnnnnnnnnnnnnnnnnnnnnnnnnnnnnnnnnnnnnnnnnnnnnnnnnnnnnnnnnnnnnnnnnnnnnnnnnnnnnnnnGTCCAGAGTCCTTAGACTGATGGAGTTTTTGGGCTTCTCTGGAGACAGGGAAGTGGGTTTGTCAGAGTTGAGAGAGGGAGCAACTACCAACAACCTGCGCTCCATCCTCAGCACCCTTACTCTGCTGATGTTTCAGCTCTACATCACAGTGATACTTGGGACTGCTGATGTAAACCTAAGTGAAnnnnnnnnnnnnnnnnnnnnnnnnnnnnnnnnnnnnnnnnnnnnnnnnnnnnnnnnnnnnnnnnnnnnnnnnnnnnnnnnnnnnnnnnnnnnnnnnnnnnnnGCCCAAGAGAAGTTCTTGGCATGTATTGCATCGCAGGAAGAGTGGCGTCAGATTCACCACCTGTGCTACTGGGAGCTGATGTGGGTCTACTCCTTTGAACAAAACTGGTTTGAGGCCTATCGATACGCCAACCTCCTCAGCAAGGAGAACAAGTGGTCCCAGGCAGTCTATGTATTTCAGAAAGCTGCCATCTTGAGCATGATGCCAGAGGAAGAAGTGAACAAGCTCAATGAAAATGTGGTGGATTTATTCAGGCAGGTGGAGGGCCTCAGGCTGAGATTTGCTGGGAAGTCGATTCCAACGGAGAAGTTTGCAGCGAAGAAGGCCCAGAGATACTCCTCTTCCACCCCCGTGAAACTTGTCGTTCCTGCTTTGGAAATGATGTATGTGTGGAATGGCTTCACAATAGTAGGCAAAAGACCTGAGCTGACTGAAGGAATCCTGTCAACTTTAGAGAAAGCAGAAGAGCAGCTCAGAAATGATCCAAACCCATCAGAGTACCACACGGGCGATGAGTGCGTTGTCCAGCTGCTGAAGGGTCTGTGCTTAAGAAGTCTGGGACGGCTGGTCCAGGCTGAGATCTGCTTCAATTATGTAATCTCCAGTGATGGTGGCATCAAGCATGATAGCTATCTGGTGCCCTTTACCATGTATGAGCTTGGCCTATTGCACAAGCAGAAAGGTGAAATCAACAAGGCCATTGCTGTAATGGAAAATGTCATGACTAACTACAAGGACTACAACATGGAGTCAAGGCTGCATTTCCGCATCCATGCAGCACTCAACACCATGGGCTCCTTTGCAGCCAAACTTCCACCAACACGCACGGCAGTTTAAGAGATCCCAT-----------------------------------------------

>TbY6.TM1 T0000032630-R1

---------nnnnnnnnnnnnnnnnnnnnnnnnnnnnnnnnnnnnnnnnnnnnnnnnnnnnnnnnnnnnnnnnnnnnnnnnnnnnnnnnnnnnnnnnnnnnnnnnnnnnnnnnnnnnnnnnnnnnnnnnnnnnnnnnnnnnnnnnnnnnnnnnnnnnnnnnnnnnnnnnnnnnnnnnnnnnnnnnnnnnnnnnGTGAAAGTATGTACCATGCGATGGGCTACAGTAGCATTCTGGTGATGCAGGCCGCCATGACTTTTGATGCAAAGGACATGGATGCTGCCATGACATCACTGAGAGAAGCCTTGCAGGTCTGCCAGAGATTTCGGAAGAAAACGGGACTAATGGAAAGCTTGGCTAACCTTTGGTACAGACAACCAGCTGACAATCTGACAGAAGAAGAGATGCATGCAGAGCTGTGCTATGCTGAAGTCCTGCTGCAGAAAGCTGCCCTCACGTTCTTGGATGAAAGCATAATAGGCTTCATCAAAGGAGGGATGAAAATTCGACACAGTTATCAGCTTTACAAGGATTGCCAGGCCATGGAAAAAGTCACAAAGGATGAGGAAAAACAGAGAAGCGCACACATTCATTTTAGGGGTGGGnnnnnnnnnnnnnnnnnnnnnnnnnnnnnnnnnnnnnnnnnnnnnnnnnnnnnnnnnnnnnnnnnnnnnnnnnnnnnnnnnnnnnCTGGAGACAGGGAAGTGGGTTTGTCAGAGTTGAGAGAGGGAGCAACTACCAACAACCTGCGCTCCATCCTCAGCACCCTCACTCTGCTGATGTTTCAGCTCTACATCACAGTGATACTTGGGACTGCTGATGTAAACCTAAGTGAATGTGAGGCTCTGCTGAAACCCTACTGTGAAAAGTTTCCTAATGGGGCTTTAATGCATTTCTTCAGTGCAAGGATTGCTGTGCTCAAAGGAAACTTCACATTTGCCCAAGAGAAGTTCTTGGCATGTATTNCATCGCAGGAAGAGTGGCGTCAGATTCACCACCTGTGCTACTGGGAGCTGATGTGGGTCTACTCCTTTGAACAAAACTGGTTTGAGGCCTATCGATACGCCAACYTSCTCAGCAAGGAGAACAAGTGGTCCCAGGCAGTCTATGTATTTCAGAAAGCTGCCATCTTGAGCATGATGCCAGAGGAAGAAGTGAACAAGCTCAATGAAAATGTGGTGGATTTATTCAnnnnnnnnnnnnnnnnnnnnCTGAGATTTGCTGGGAAGTCGATTCCAACGGAGAAGTTTGCAGCGAAGAAGGCCCAGAGATACTCCTCTTCCACCCCCGTGAAACTTGTCGTTCCTGCTTTGGAAATGATGTATGTGTGGAATGGnnnnnnnnnnnnnGGCAAAAGACCCGAGCTGACTGAAGGAATCCTGTCAACTTTAGAGAAAGCAGAAGAGCAGCTCAGAAATGATCCAAACCCATCAGAGTACCACACGGGCGATGAGTGCGTTGTCCAGCTGCTGAAGGGTCTGTGCTTAAGAAGTCTGGGACGGCTGGTCCAGGCTGAGATCTGCTTCAATTATGTAATCTCCAGTGATGGTGGCATCAAGCATGATAGCTATCTGGTGCCCTTTACCATGTATGAGCTTGGCCTATTGCACAAGCAGAAAGGTGAAATCAACAAGGCCATTGCTGTAATGGAAAATGTCATGACTAACTACAAGGACTACAACATGGAGTCAAGGCTGCATTTCCGCATCCATGCAGCACTCAACACCATGGGCTCCTTTGCAGCCAAACTTCCACCAA--------------------------------------------------------------------------

>TmR1.TM1 T0000032630-R1

---------ATGGAGGGTGAAGACAATTCACTACAACAGGTGGGTTTGAAGCAGCCCAATGAGGATACAATATTGCCTGAAGAATTGCCTAAAATGGGTTTGGAGACAACATTGAAAGAATGCTTCACTGCCCTCAATTTCTTCCTGAACAACAGGTTTGCCGATGCATTGGCTCTTTTAAAACCCTGGAAGAGTGAAAGTATGTACCATGCGATGGGCTACAGTAGCATTCTGGTGATGCAGGCCGCCATGACTTTTGATGCAAAGGACATGGATGCTGCCATGACATCACTGAGAGAAGCCTTGCAGGTCTGCCAGAGATTTCGGAAGAAAACGGGACTAATGGAAAGCTTGGCTAACCTTTGGTACAGACAACCAGCTGACAATCTGACAGAAGAAGAGATGCATGCAGAGCTGTGCTATGCTGAAGTCCTGCTGCAGAAAGCTGCCCTCACGTTCTTGGATGAAAGCATAATAGGCTTCATCAAAGGAGGGATGAAAATTCGACACAGTTATCAGCTTTACAAGGATTGCCAGGCCATGGAAAAAGTCACAAAGGATGAGGAAAAACAGAGAAGCACACACATTCATTTTAGGGGTGGGGTCAGCATGGGAATTGGATCATTCAATCTGATGCTGTCTCTGCTTCCGTCCAGAGTCCTTAGACTGATGGAGTTTTTGGGCTTCTCTGGAGACAGGGAAGTGGGTTTGTCAGAGTTGAGAGAGGGAGCAACTACCAACAACCTGCGCTCCATCCTCAGCACCCTCACTCTGCTGATGTTTCAGCTCTACATCACAGTGATACTTGGGACTGCTGATGTAAACCTAAGTGAATGTGAGGCTCTGCTGAAACCCTACTGTGAAAAGTTTCCTAATGGGGCTTTAATGCATTTCTTCAGTGCAAGGATTGCTGTGCTCAAAGGAAACTTCACATTTGCCCAAGAGAAGTTCTTGGCATGTATTGCATTGCAGGAAGAGTGGCGTCAGATTCACCACCTGTGCTACTGGGAGCTGATGTGGGTCTACTCCTTTGAACAAAACTGGTTTGAGGCCTATCGATACGCCAACCTCCTCAGCAAGGAGAACAAGTGGTCCCAGGCAGTCTATGTATTTCAGAAAGCTGCCATCTTGAGCATGATGCCAGAGGAAGAAGTGAACAAGCTCAATGAAAATGTGGTGGAATTATTCAGGCAGGTGGAGGGCCTCAGGCTGAGATTTGCTGGGAAGTCGATTCCAACGGAGAAGTTTGCAGCAAAGAAGGCCCAGAGATACTCCTCTTCCACCCCCGTGAAACTTGTCGTTCCTGCTTTGGAAATGATGTATGTGTGGAATGGCTTCACAATAGTAGGCAAAAGACCCGAGCTGACTGAAGGAATCCTGTCAACTTTAGAGAAAGCAGAAGAGCAGCTCAGAAATGATCCGAACCCATCAGAGTACCACACGGGCGATGAGTGCGTTGTCCAGCTGCTGAAGGGTCTGTGCTTAAGAAGTCTGGGACAGCTGGTCCAGGCTGAGATCTGCTTCAATTATGTAATCTCCAGTGATGGTGGCATCAAGCATGATAGCTATCTGGTGCCCTTTACCATGTATGAGCTTGGCCTATTGCACAAGCAGAAAGGTGAAATCAACAAGGCCATTGCTGTAATGGAAAATGTCATGACGAACTACAAGGACTACAACATGGAGTCAAGGCTGCATTTCCGCATCCATGCAGCACTCAACACCATGGGCTCCTTTGCAGCCAAACTTCCACCAACACGCACGGCAGTTTAAGAGATCCCATTACATGAAACTTGACAGTGTTTACTGTTTCATTGTCTGTTGCCTTCA

>TmR2.TM1 T0000032630-R1

---------ATGGAGGGTGAAGACAATTCACTACAACAGGTGGGTTTGAAGCAGCCCAATGAGGATACAATATTGCCTGAAGAATTGCCTAAAATGGGTTTGGAGACAGCATTGAAAGAATGCTTCACTGCCCTCAATTTCTTCCTGAACAACAGGTTTGCCGATGCATTGGCTCTTTTAAAACCCTGGAAGAGTGAAAGTATGTACCATGCGATGGGCTACAGTAGCATTCTGGTGATGCAGGCCGCCATGACTTTTGATGCAAAGGACATGGATGCTGCCATGACATCACTGAGAGAAGCCTTGCAGGTCTGCCAGAGATTTCGGAAGAAAACGGGACTAATGGAAAGCTTGGCTAACCTTTGGTACAGACAACCAGCTGACAATCTGACAGAAGAAGAGATGCATGCAGAGCTGTGCTATGCTGAAGTCCTGCTGCAGAAAGCTGCCCTCACGTTCTTGGATGAAAGCATAATAGGCTTCATCAAAGGAGGGATGAAAATTCGACACAGTTATCAGCTTTACAAGGATTGCCAGGCCATGGAAAAAGTCACAAAGGATGAGGAAAAACAGAGAAGCACACACATTCATTTTAGGGGTGGGGTCAGCATGGGAATTGGATCATTCAATCTGATGCTGTCTCTGCTTCCGTCCAGAGTCCTTAGACTGATGGAGTTTTTGGGCTTCTCTGGAGACAGGGAAGTGGGTTTGTCAGAGTTGAGAGAGGGAGCAAYTACCAACAACCTGCGCTCCATCCTCAGCACCCTCACTSTGCTGATGTTTCAGCTCTACATCACAGTGATACTTGGGACTGCTGATGTAAACCTAAGTGAATGTGAGGCTCTGCTGAAACCCTACTGTGAAAAGTTTCCTAATGGGGCTTTAATGCATTTCTTCAGTGCAAGGATTGCTGTGCTCAAAGGAAACTTCACATTTGCCCAAGAGAAGTTCTTGGCATGTATTGCATTGCAGGAAGAGTGGCGTCAGATTCACCACCTGTGCTACTGGGAGCTGATGTGGGTCTACTCCTTTGAACAAAACTGGTTTGAGGCCTATCGATACGCCAACCTCCTCAGCAAGGAGAACAAGTGGTCCCAGGCAGTCTATGTATTTCAGAAAGCTGCCATCTTGAGCATGATGCCAGAGGAAGAAGTGAACAAGCTCAATGAAAATGTGGTGGAATTATTCAGGCAGGTGGAGGGCCTCAGGCTGAGATTTGCTGGGAAGTCGATTCCAACGGAGAAGTTTGCAGCAAAGAAGGCCCAGAGATACTCCTCTTCCACCCCCGTGAAACTTGTCGTTCCTGCTTTGGAAATGATGTATGTGTGGAATGGCTTCACAATAGTAGGCAAAAGACCCGAGCTGACTGAAGGAATCCTGTCAACTTTAGAGAAAGCAGAAGAGCAGCTCAGAAATGATCCGAACCCATCAGAGTACCACACGGGCGATGAGTGCGTTGTCCAGCTGCTGAAGGGTCTGTGCTTAAGAAGTCTGGGACAGCTGGTCCAGGCTGAGATCTGCTTCAATTATGTAATCTCCAGTGATGGTGGCATCAAGCATGATAGCTATCTGGTGCCCTTTACCATGTATGAGCTTGGCCTATTGCACAAGCAGAAAGGTGAAATCAACAAGGCCATTGCTGTAATGGAAAATGTCATGACGAACTACAAGGACTACAACATGGAGTCAAGGCTGCATTTCCGCATCCATGCAGCACTCAACACCATGGGCTCCTTTGCAGCCAAACTTCCACCAACACGCACRGCAGTTTAAGAGATCCCATTACATGAAACTTGACAGTGTTTACTGTTTCATTGTCTGTTGCCTTC-

>TmR3.TM1 T0000032630-R1

---------ATGGAGGGTGAAGACAATTCACTACAACAGGTGGGTTTGAAGCAGCCCAATGAGGATACAATATTGCCTGAAGAATTGCCTAAAATGGGTTTGGAGACAGCATTGAAAGAATGCTTCACTGCCCTCAATTTCTTCCTGAACAACAGGTTTGCCGATGCATTGGCTCTTTTAAAACCCTGGAAGAGTGAAAGTATGTACCATGCGATGGGCTACAGTAGCATTCTGGTGATGCAGGCCGCCATGACTTTTGATGCAAAGGACATGGATGCTGCCATGACATCACTGAGAGAAGCCTTGCAGGTCTGCCAGAGATTTCGGAAGAAAACGGGACTAATGGAAAGCTTGGCTAACCTTTGGTACAGACAACCAGCTGACAATCTGACAGAAGAAGAGATGCATGCAGAGCTGTGCTATGCTGAAGTCCTGCTGCAGAAAGCTGCCCTCAYGTTCTTGGATGAAAGCATAATAGGCTTCATCAAAGGAGGGATGAAAATTCGACACAGTTATCAGCTTTACAAGGATTGCCAGGCCATGGAAAAAGTCACAAAGGATGAGGAAAAACAGAGAAGCACACACATTCATTTTAGGGGTGGGGTCAGCATGGGAATTGGATCATTCAATCTGATGCTGTCTCTGCTTCCGTCCAGAGTCCTTAGACTGATGGAGTTTTTGGGCTTCTCTGGAGACAGGGAAGTGGGTTTGTCAGAGTTGAGAGAGGGAGCAACTACCAACAACCTGCGCTCCATCCTCAGCACCCTCACTSTGCTGATGTTTCAGCTCTACATCACAGTGATACTTGGGACTGCTGATGTAAACCTAAGTGAATGTGAGGCTCTGCTGAAACCCTACTGTGAAAAGTTTCCTAATGGGGCTTTAATGCATTTCTTCAGTGCAAGGATTGCTGTGCTCAAAGGAAACTTCACATTTGCCCAAGAGAAGTTCTTGGCATGTATTGCATTGCAGGAAGAGTGGCGTCAGATTCACCACCTGTGCTACTGGGAGCTGATGTGGGTCTACTCCTTTGAACAAAACTGGTTTGAGGCCTATCGATACGCCAACCTCCTCAGCAAGGAGAACAAGTGGTCCCAGGCAGTCTATGTATTTCAGAAAGCTGCCATCTTGAGCATGATGCCAGAGGAAGAAGTGAACAAGCTCAATGAAAATGTGGTGGAATTATTCAGGCAGGTGGAGGGCCTCAGGCTGAGATKTGCTGGGAAGTCGATTCCAACGGAGAAGTTTGCAGCAAAGAAGGCCCAGAGATACTCCTCTTCCACCCCCGTGAAACTTGTCGTTCCTGCTTTGGAAATGATGTATGTGTGGAATGGCTTCACAATAGTAGGCAAAAGACCCGAGCTGACTGAAGGAATCCTGTCAACTTTAGAGAAAGCAGAAGAGCAGCTCAGAAATGATCCGAACCCATCAGAGTACCACACGGGCGATGAGTGCGTTGTCCAGCTGCTGAAGGGTCTGTGCTTAAGAAGTCTGGGACAGCTGGTCCAGGCTGAGATCTGCTTCAATTATGTAATCTCCAGTGATGGTGGCATCAAGCATGATAGCTATCTGGTGCCCTTTACCATGTATGAGCTTGGCCTATTGCACAAGCAGAAAGGTGAAATCAACAAGGCCATTGCTGTAATGGAAAATGTCATGACGAACTACAAGGACTACAACATGGAGTCAAGGCTGCATTTCCGCATCCATGCAGCACTCAACACCATGGGCTCCTTTGCAGCCAAACTTCCACCAACACGCACGGCAGTTTAAGAGATCCCATTACATGAAACTTGACAGTGTTTACTGTTTCATTGTCTGTTGCCTTCA

>TmR4.TM1 T0000032630-R1

---------ATGGAGGGTGAAGACAATTCACTACAACAGGTGGGTTTGAAGCAGCCCAATGAGGATACAATATTGCCTGAAGAATTGCCTAAAATGGGTTTGGAGACAACATTGAAAGAATGCTTCACTGCCCTCAATTTCTTCCTGAACAACAGGTTTGCCGATGCATTGGCTCTTTTAAAACCCTGGAAGAGTGAAAGTATGTACCATGCGATGGGCTACAGTAGCATTCTGGTGATGCAGGCCGCCATGACTTTTGATGCAAAGGACATGGATGCTGCCATGACATCACTGAGAGAAGCCTTGCAGGTCTGCCAGAGATTTCGGAAGAAAACGGGACTAATGGAAAGCTTGGCTAACCTTTGGTACAGACAACCAGCTGACAATCTGACAGAAGAAGAGATGCATGCAGAGCTGTGCTATGCTGAAGTCCTGCTGCAGAAAGCTGCCCTCACGTTCTTGGATGAAAGCATAATAGGCTTCATCAAAGGAGGGATGAAAATTCGACACAGTTATCAGCTTTACAAGGATTGCCAGGCCATGGAAAAAGTCACAAAGGATGAGGAAAAACAGAGAAGCACACACATTCATTTTAGGGGTGGGGTCAGCATGGGAATTGGATCATTCAATCTGATGCTGTCTCTGCTTCCGTCCAGAGTCCTTAGACTGATGGAGTTTTTGGGCTTCTCTGGAGACAGGGAAGTGGGTTTGTCAGAGTTGAGAGAGGGAGCAACTACCAACAACCTGCGCTCCATCCTCAGCACCCTCACTCTGCTGATGTTTCAGCTCTACATCACAGTGATACTTGGGACTGCTGATGTAAACCTAAGTGAATGTGAGGCTCTGCTGAAACCCTACTGTGAAAAGTTTCCTAATGGGGCTTTAATGCATTTCTTCAGTGCAAGGATTGCTGTGCTCAAAGGAAACTTCACATTTGCCCAAGAGAAGTTCTTGGCATGTATTGCATTGCAGGAAGAGTGGCGTCAGATTCACCACCTGTGCTACTGGGAGCTGATGTGGGTCTACTCCTTTGAACAAAACTGGTTTGAGGCCTATCGATACGCCAACCTCCTCAGCAAGGAGAACAAGTGGTCCCAGGCAGTCTATGTATTTCAGAAAGCTGCCATCTTGAGCATGATGCCAGAGGAAGAAGTGAACAAGCTCAATGAAAATGTGGTGGAATTATTCAGGCAGGTGGAGGGCCTCAGGCTGAGATTTGCTGGGAAGTCGATTCCAACGGAGAAGTTTGCAGCAAAGAAGGCCCAGAGATACTCCTCTTCCACCCCCGTGAAACTTGTCGTTCCTGCTTTGGAAATGATGTATGTGTGGAATGGCTTCACAATAGTAGGCAAAAGACCCGAGCTGACTGAAGGAATCCTGTCAACTTTAGAGAAAGCAGAAGAGCAGCTCAGAAATGATCCGAACCCATCAGAGTACCACACGGGCGATGAGTGCGTTGTCCAGCTGCTGAAGGGTCTGTGCTTAAGAAGTCTGGGACAGCTGGTCCAGGCTGAGATCTGCTTCAATTATGTAATCTCCAGTGATGGTGGCATCAAGCATGATAGCTATCTGGTGCCCTTTACCATGTATGAGCTTGGCCTATTGCACAAGCAGAAAGGTGAAATCAACAAGGCCATTGCTGTAATGGAAAATGTCATGACGAACTACAAGGACTACAACATGGAGTCAAGGCTGCATTTCCGCATCCATGCAGCACTCAACACCATGGGCTCCTTTGCAGCCAAACTTCCACCAACACGCACGGCAGTTTAAGAGATCCCATTACATGAAACTTGACAGTGTTTACTGTTTCATTGTCTGTTGCCTTCA

>TmR5.TM1 T0000032630-R1

---------ATGGAGGGTGAAGACAATTCACTACAACAGGTGGGTTTGAAGCAGCCCAATGAGGATACAATATTGCCTGAAGAATTGCCTAAAATGGGTTTGGAGACAACATTGAAAGAATGCTTCACTGCCCTCAATTTCTTCCTGAACAACAGGTTTGCCGATGCATTGGCTCTTTTAAAACCCTGGAAGAGTGAAAGTATGTACCATGCGATGGGCTACAGTAGCATTCTGGTGATGCAGGCCGCCATGACTTTTGATGCAAAGGACATGGATGCTGCCATGACATCACTGAGAGAAGCCTTGCAGGTCTGCCAGAGATTTCGGAAGAAAACGGGACTAATGGAAAGCTTGGCTAACCTTTGGTACAGACAACCAGCTGACAATCTGACAGAAGAAGAGATGCATGCAGAGCTGTGCTATGCTGAAGTCCTGCTGCAGAAAGCTGCCCTCACGTTCTTGGATGAAAGCATAATAGGCTTCATCAAAGGAGGGATGAAAATTCGACACAGTTATCAGCTTTACAAGGATTGCCAGGCCATGGAAAAAGTCACAAAGGATGAGGAAAAACAGAGAAGCACACACATTCATTTTAGGGGTGGGGTCAGCATGGGAATTGGATCATTCAATCTGATGCTGTCTCTGCTTCCGTCCAGAGTCCTTAGACTGATGGAGTTTTTGGGCTTCTCTGGAGACAGGGAAGTGGGTTTGTCAGAGTTGAGAGAGGGAGCAACTACCAACAACCTGCGCTCCATCCTCAGCACCCTCACTCTGCTGATGTTTCAGCTCTACATCACAGTGATACTTGGGACTGCTGATGTAAACCTAAGTGAATGTGAGGCTCTGCTGAAACCCTACTGTGAAAAGTTTCCTAATGGGGCTTTAATGCATTTCTTCAGTGCAAGGATTGCTGTGCTCAAAGGAAACTTCACATTTGCCCAAGAGAAGTTCTTGGCATGTATTGCATTGCAGGAAGAGTGGCGTCAGATTCACCACCTGTGCTACTGGGAGCTGATGTGGGTCTACTCCTTTGAACAAAACTGGTTTGAGGCCTATCGATACGCCAACCTCCTCAGCAAGGAGAACAAGTGGTCCCAGGCAGTCTATGTATTTCAGAAAGCTGCCATCTTGAGCATGATGCCAGAGGAAGAAGTGAACAAGCTCAATGAAAATGTGGTGGAATTATTCAGGCAGGTGGAGGGCCTCAGGCTGAGATTTGCTGGGAAGTCGATTCCAACGGAGAAGTTTGCAGCAAAGAAGGCCCAGAGATACTCCTCTTCCACCCCCGTGAAACTTGTCGTTCCTGCTTTGGAAATGATGTATGTGTGGAATGGCTTCACAATAGTAGGCAAAAGACCCGAGCTGACTGAAGGAATCCTGTCAACTTTAGAGAAAGCAGAAGAGCAGCTCAGAAATGATCCGAACCCATCAGAGTACCACACGGGCGATGAGTGCGTTGTCCAGCTGCTGAAGGGTCTGTGCTTAAGAAGTCTGGGACAGCTGGTCCAGGCTGAGATCTGCTTCAATTATGTAATCTCCAGTGATGGTGGCATCAAGCATGATAGCTATCTGGTGCCCTTTACCATGTATGAGCTTGGCCTATTGCACAAGCAGAAAGGTGAAATCAACAAGGCCATTGCTGTAATGGAAAATGTCATGACGAACTACAAGGACTACAACATGGAGTCAAGGCTGCATTTCCGCATCCATGCAGCACTCAACACCATGGGCTCCTTTGCAGCCAAACTTCCACCAACACGCACGGCAGTTTAAGAGATCCCATTACATGAAACTTGACAGTGTTTACTGTTTCATTGTCTGTTGCCTTCA

>TmR6.TM1 T0000032630-R1

---------ATGGAGGGTGAAGACAATTCACTACAACAGGTGGGTTTGAAGCAGCCCAATGAGGATACAATATTGCCTGAAGAATTGCCTAAAATGGGTTTGGAGACAACATTGAAAGAATGCTTCACTGCCCTCAATTTCTTCCTGAACAACAGGTTTGCCGATGCATTGGCTCTTTTAAAACCCTGGAAGAGTGAAAGTATGTACCATGCGATGGGCTACAGTAGCATTCTGGTGATGCAGGCCGCCATGACTTTTGATGCAAAGGACATGGATGCTGCCATGACATCACTGAGAGAAGCCTTGCAGGTCTGCCAGAGATTTCGGAAGAAAACGGGACTAATGGAAAGCTTGGCTAACCTTTGGTACAGACAACCAGCTGACAATCTGACAGAAGAAGAGATGCATGCAGAGCTGTGCTATGCTGAAGTCCTGCTGCAGAAAGCTGCCCTCACGTTCTTGGATGAAAGCATAATAGGCTTCATCAAAGGAGGGATGAAAATTCGACACAGTTATCAGCTTTACAAGGATTGCCAGGCCATGGAAAAAGTCACAAAGGATGAGGAAAAACAGAGAAGCACACACATTCATTTTAGGGGTGGGGTCAGCATGGGAATTGGATCATTCAATCTGATGCTGTCTCTGCTTCCGTCCAGAGTCCTTAGACTGATGGAGTTTTTGGGCTTCTCTGGAGACAGGGAAGTGGGTTTGTCAGAGTTGAGAGAGGGAGCAACTACCAACAACCTGCGCTCCATCCTCAGCACCCTCACTCTGCTGATGTTTCAGCTCTACATCACAGTGATACTTGGGACTGCTGATGTAAACCTAAGTGAATGTGAGGCTCTGCTGAAACCCTACTGTGAAAAGTTTCCTAATGGGGCTTTAATGCATTTCTTCAGTGCAAGGATTGCTGTGCTCAAAGGAAACTTCACATTTGCCCAAGAGAAGTTCTTGGCATGTATTGCATTGCAGGAAGAGTGGCGTCAGATTCACCACCTGTGCTACTGGGAGCTGATGTGGGTCTACTCCTTTGAACAAAACTGGTTTGAGGCCTATCGATACGCCAACCTCCTCAGCAAGGAGAACAAGTGGTCCCAGGCAGTCTATGTATTTCAGAAAGCTGCCATCTTGAGCATGATGCCAGAGGAAGAAGTGAACAAGCTCAATGAAAATGTGGTGGAATTATTCAGGCAGGTGGAGGGCCTCAGGCTGAGATTTGCTGGGAAGTCGATTCCAACGGAGAAGTTTGCAGCAAAGAAGGCCCAGAGATACTCCTCTTCCACCCCCGTGAAACTTGTCGTTCCTGCTTTGGAAATGATGTATGTGTGGAATGGCTTCACAATAGTAGGCAAAAGACCCGAGCTGACTGAAGGAATCCTGTCAACTTTAGAGAAAGCAGAAGAGCAGCTCAGAAATGATCCGAACCCATCAGAGTACCACACGGGCGATGAGTGCGTTGTCCAGCTGCTGAAGGGTCTGTGCTTAAGAAGTCTGGGACAGCTGGTCCAGGCTGAGATCTGCTTCAATTATGTAATCTCCAGTGATGGTGGCATCAAGCATGATAGCTATCTGGTGCCCTTTACCATGTATGAGCTTGGCCTATTGCACAAGCAGAAAGGTGAAATCAACAAGGCCATTGCTGTAATGGAAAATGTCATGACGAACTACAAGGACTACAACATGGAGTCAAGGCTGCATTTCCGCATCCATGCAGCACTCAACACCATGGGCTCCTTTGCAGCCAAACTTCCACCAACACGCACGGCAGTTTAAGAGATCCCATTACATGAAACTTGACAGTGTTTACTGTTTCATTGTCTGTTGCCTTCA

>TmY1.TM1 T0000032630-R1

---------ATGGAGGGTGAAGACAATTCACTACAACAGGTGGGTTTGAAGCAGCCCAATGAGGATACAATATTGCCTGAAGAATTGCCTAAAATGGGTTTGGAGACAGCATTGAAAGAATGCTTCACTGCCCTCAATTTCTTCCTGAACAACAGGTTTGCCGATGCATTGGCTCTTTTAAAACCCTGGAAGAGTGAAAGTATGTACCATGCGATGGGCTACAGTAGCATTCTGGTGATGCAGGCCGCCATGACTTTTGATGCAAAGGACATGGATGCTGCCATGACATCACTGAGAGAAGCCTTGCAGGTCTGCCAGAGATTTCGGAAGAAAACGGGACTAATGGAAAGCTTGGCTAACCTTTGGTACAGACAACCAGCTGACAATCTGACAGAAGAAGAGATGCATGCAGAGCTGTGCTATGCTGAAGTCCTGCTGCAGAAAGCTGCCCTCACGTTCTTGGATGAAAGCATAATAGGCTTCATCAAAGGAGGGATGAAAATTCGACACAGTTATCAGCTTTACAAGGATTGCCAGGCCATGGAAAAAGTCACAAAGGATGAGGAAAAACAGAGAAGCRCACACATTCATTTTAGGGGTGGGGTCAGCATGGGAATTGGATCATTCAATCTGATGCTGTCTCTGCTTCCGTCCAGAGTCCTTAGACTGATGGAGTTTTTGGGCTTCTCTGGAGACAGGGAAGTGGGTTTGTCAGAGTTGAGAGAGGGAGCAACTACCAACAACCTGCGCTCCATCCTCAGCACCCTCACTCTGCTGATGTTTCAGCTCTACATCACAGTGATACTTGGGACTGCTGATGTAAACCTAAGTGAATGTGAGGCTCTGCTGAAACCCTACTGTGAAAAGTTTCCTAATGGGGCTTTAATGCATTTCTTCAGTGCAAGGATTGCTGTGCTCAAAGGAAACTTCACATTTGCCCAAGAGAAGTTCTTGGCATGYATTGCATYGCAGGAAGAGTGGCGTCAGATTCACCACCTGTGCTACTGGGAGCTGATGTGGGTCTACTCCTTTGAACAAAACTGGTTTGAGGCCTATCGATACGCCAACCTCCTCAGCAAGGAGAACAAGTGGTCCCAGGCAGTCTATGTATTTCAGAAAGCTGCCATCTTGAGCATGATGCCAGAGGAAGAAGTGAACAAGCTCAATGAAAATGTGGTGGAATTATTCAGGCAGGTGGAGGGCCTCAGGCTGAGATTTGCTGGGAAGTCGATTCCAACGGAGAAGTTTGCAGCGAAGAAGGCCCAGAGATACTCCTCTTCCACCMCCGTGAAACTTGTCGTTCCTGCTTTGGAAATGATGTATGTGTGGAATGGCTTCACAATAGTAGGCAAAAGACCCGAGCTGACTGAAGGAATCCTGTCAACTTTAGAGAAAGCAGAAGAGCAGCTCAGAAATGATCCGAACCCATCAGAKTACCACACGGGCGATGAGTGCGTTGTCCAGCTGCTGAAGGGTCTGTGCTTAAGAAGTCTGGGACGGCTGGTCCAGGCTGAGATCTGCTTCAATTATGTAATCTCCAGTGATGGTGGCATCAAGCATGATAGCTATCTGGTGCCCTTTACCATGTATGAGCTTGGCCTATTGCACAAGCAGAAAGGTGAAATCAACAAGGCCATTGCTGTAATGGAAAATGTCATGACGAACTACAAGGACTACAACATGGAGTCAAGGCTGCATTTCCGCATCCATGCAGCACTCAACACCATGGGCTCCTTTGCAGCCAAACTTCCACCAACACGCACGGCAGTTTAAGAGATCCCATTACATGAAACTTGACAGTGTTTACTGTTTCATTGTCTGTTGCCTTC-

>TmY2.TM1 T0000032630-R1

---------ATGGAGGGTGAAGACAATTCACTACAACAGGTGGGTTTGAAGCAGCCCAATGAGGATACAATATTGCCTGAAGAATTGCCTAAAATGGGTTTGGAGACAGCATTGAAAGAATGCTTCACTGCCCTCAATTTCTTCCTGAACAACAGGTTTGCCGATGCATTGGCTCTTTTAAAACCCTGGAAGAGTGAAAGTATGTACCATGCGATGGGCTACAGTAGCATTCTGGTGATGCAGGCCGCCATGACTTTTGATGCAAAGGACATGGATGCTGCCATGACATCACTGAGAGAAGCCTTGCAGGTCTGCCAGAGATTTCGGAAGAAAACGGGACTAATGGAAAGCTTGGCTAACCTTTGGTACAGACAACCAGCTGACAATCTGACAGAAGAAGAGATGCATGCAGAGCTGTGCTATGCTGAAGTCCTGCTGCAGAAAGCTGCCCTCACGTTCTTGGATGAAAGCATAATAGGCTTCATCAAAGGAGGGATGAAAATTCGACACAGTTATCAGCTTTACAAGGATTGCCAGGCCATGGAAAAAGTCACAAAGGATGAGGAAAAACAGAGAAGCGCACACATTCATTTTAGGGGTGGGGTCAGCATGGGAATTGGATCATTCAATCTGATGCTGTCTCTGCTTCCGTCCAGAGTCCTTAGACTGATGGAGTTTTTGGGCTTCTCTGGAGACAGGGAAGTGGGTTTGTCAGAGTTGAGAGAGGGAGCAACTACCAACAACCTGCGCTCCATCCTCAGCACCCTCACTCTGCTGATGTTTCAGCTCTACATCACAGTGATACTTGGGACTGCTGATGTAAACCTAAGTGAATGTGAGGCTCTGCTGAAACCCTACTGTGAAAAGTTTCCTAATGGGGCTTTAATGCATTTCTTCAGTGCAAGGATTGCTGTGCTCAAAGGAAACTTCACATTTGCCCAAGAGAAGTTCTTGGCATGYATTGCATYGCAGGAAGAGTGGCGTCAGATTCACCACCTGTGCTACTGGGAGCTGATGTGGGTCTACTCCTTTGAACAAAACTGGTTTGAGGCCTATCGATACGCCAACCTCCTCAGCAAGGAGAACAAGTGGTCCCAGGCAGTCTATGTATTTCAGAAAGCTGCCATCTTGAGCATGATGCCAGAGGAAGAAGTGAACAAGCTCAATGAAAATGTGGTGGAATTATTCAGGCAGGTGGAGGGCCTCAGGCTGAGATTTGCTGGGAAGTCGATTCCAACGGAGAAGTTTGCAGCGAAGAAGGCCCAGAGATACTCCTCTTCCACCMCCGTGAAACTTGTCGTTCCTGCTTTGGAAATGATGTATGTGTGGAATGGCTTCACAATAGTAGGCAAAAGACCCGAGCTGACTGAAGGAATCCTGTCAACTTTAGAGAAAGCAGAAGAGCAGCTCAGAAATGATCCGAACCCATCAGAKTACCACACGGGCGATGAGTGCGTTGTCCAGCTGCTGAAGGGTCTGTGCTTAAGAAGTCTGGGACGGCTGGTCCAGGCTGAGATCTGCTTCAATTATGTAATCTCCAGTGATGGTGGCATCAAGCATGATAGCTATCTGGTGCCCTTTACCATGTATGAGCTTGGCCTATTGCACAAGCAGAAAGGTGAAATCAACAAGGCCATTGCTGTAATGGAAAATGTCATGACGAACTACAAGGACTACAACATGGAGTCAAGGCTGCATTTCCGCATCCATGCAGCACTCAACACCATGGGCTCCTTTGCAGCCAAACTTCCACCAACACGCACGGCAGTTTAAGAGATCCCATTACATGAAACTTGACAGTGTTTACTGTTTCATTGTCTGTTGCCTTC-

>TmY3.TM1 T0000032630-R1

---------nnGGAGGGTGAAGACAATTCACTACAACAGGTGGGTTTGAAGCAGCCCAATGAGGATACAATATTGCCTGAAGAATTGCCTAAAATGGGTTTGGAGACAGCATTGAAAGAATGCTTCACTGCCCTCAATTTCTTCCTGAACAACAGGTTTGCCGATGCATTGGCTCTTTTAAAACCCTGGAAGAGTGAAAGTATGTACCATGCGATGGGCTACAGTAGCATTCTGGTGATGCAGGCCGCCATGACTTTTGATGCAAAGGACATGGATGCTGCCATGACATCACTGAGAGAAGCCTTGCAGGTCTGCCAGAGATTTCGGAAGAAAACGGGACTAATGGAAAGCTTGGCTAACCTTTGGTACAGACAACCAGCTGACAATCTGACAGAAGAAGAGATGCATGCAGAGCTGTGCTATGCTGAAGTCCTGCTGCAGAAAGCTGCCCTCACGTTCTTGGATGAAAGCATAATAGGCTTCATCAAAGGAGGGATGAAAATTCGACACAGTTATCAGCTTTACAAGGATTGCCAGGCCATGGAAAAAGTCACAAAGGATGAGGAAAAACAGAGAAGCRCACACATTCATTTTAGGGGTGGGGTCAGCATGGGAATTGGATCATTCAATCTGATGCTGTCTCTGCTTCCGTCCAGAGTCCTTAGACTGATGGAGTTTTTGGGCTTCTCTGGAGACAGGGAAGTGGGTTTGTCAGAGTTGAGAGAGGGAGCAACTACCAACAACCTGCGCTCCATCCTCAGCACCCTCACTCTGCTGATGTTTCAGCTCTACATCACAGTGATACTTGGGACTGCTGATGTAAACCTAAGTGAATGTGAGGCTCTGCTGAAACCCTACTGTGAAAAGTTTCCTAATGGGGCTTTAATGCATTTCTTCAGTGCAAGGATTGCTGTGCTCAAAGGAAACTTCACATTTGCCCAAGAGAAGTTCTTGGCATGYATTGCATYGCAGGAAGAGTGGCGTCAGATTCACCACCTGTGCTACTGGGAGCTGATGTGGGTCTACTCCTTTGAACAAAACTGGTTTGAGGCCTATCGATACGCCAACCTCCTCAGCAAGGAGAACAAGTGGTCCCAGGCAGTCTATGTATTTCAGAAAGCTGCCATCTTGAGCATGATGCCAGAGGAAGAAGTGAACAAGCTCAATGAAAATGTGGTGGAATTATTCAGGCAGGTGGAGGGCCTCAGGCTGAGATTTGCTGGGAAGTCGATTCCAACGGAGAAGTTTGCAGCGAAGAAGGCCCAGAGATACTCCTCTTCCACCMCCGTGAAACTTGTCGTTCCTGCTTTGGAAATGATGTATGTGTGGAATGGCTTCACAATAGTAGGCAAAAGACCCGAGCTGACTGAAGGAATCCTGTCAACTTTAGAGAAAGCAGAAGAGCAGCTCAGAAATGATCCGAACCCATCAGAKTACCACACGGGCGATGAGTGCGTTGTCCAGCTGCTGAAGGGTCTGTGCTTAAGAAGTCTGGGACGGCTGGTCCAGGCTGAGATCTGCTTCAATTATGTAATCTCCAGTGATGGTGGCATCAAGCATGATAGCTATCTGGTGCCCTTTACCATGTATGAGCTTGGCCTATTGCACAAGCAGAAAGGTGAAATCAACAAGGCCATTGCTGTAATGGAAAATGTCATGACGAACTACAAGGACTACAACATGGAGTCAAGGCTGCATTTCCGCATCCATGCAGCACTCAACACCATGGGCTCCTTTGCAGCCAAACTTCCACCAACACGCACGGCAGTTTAAGAGATCCCATTACATGAAACTTGACAGTGTTTACTGTTTCATTGTCTGTTGCCT---

>TmY4.TM1 T0000032630-R1

---------ATGGAGGGTGAAGACAATTCACTACAACAGGTGGGTTTGAAGCAGCCCAATGAGGATACAATATTGCCTGAAGAATTGCCTAAAATGGGTTTGGAGACAGCATTGAAAGAATGCTTCACTGCCCTCAATTTCTTCCTGAACAACAGGTTTGCCGATGCATTGGCTCTTTTAAAACCCTGGAAGAGTGAAAGTATGTACCATGCGATGGGCTACAGTAGCATTCTGGTGATGCAGGCCGCCATGACTTTTGATGCAAAGGACATGGATGCTGCCATGACATCACTGAGAGAAGCCTTGCAGGTCTGCCAGAGATTTCGGAAGAAAACGGGACTAATGGAAAGCTTGGCTAACCTTTGGTACAGACAACCAGCTGACAATCTGACAGAAGAAGAGATGCATGCAGAGCTGTGCTATGCTGAAGTCCTGCTGCAGAAAGCTGCCCTCACGTTCTTGGATGAAAGCATAATAGGCTTCATCAAAGGAGGGATGAAAATTCGACACAGTTATCAGCTTTACAAGGATTGCCAGGCCATGGAAAAAGTCACAAAGGATGAGGAAAAACAGAGAAGCGCACACATTCATTTTAGGGGTGGGGTCAGCATGGGAATTGGATCATTCAATCTGATGCTGTCTCTGCTTCCGTCCAGAGTCCTTAGACTGATGGAGTTTTTGGGCTTCTCTGGAGACAGGGAAGTGGGTTTGTCAGAGTTGAGAGAGGGAGCAACTACCAACAACCTGCGCTCCATCCTCAGCACCCTCACTCTGCTGATGTTTCAGCTCTACATCACAGTGATACTTGGGACTGCTGATGTAAACCTAAGTGAATGTGAGGCTCTGCTGAAACCCTACTGTGAAAAGTTTCCTAATGGGGCTTTAATGCATTTCTTCAGTGCAAGGATTGCTGTGCTCAAAGGAAACTTCACATTTGCCCAAGAGAAGTTCTTGGCATGTATTGCATCGCAGGAAGAGTGGCGTCAGATTCACCACCTGTGCTACTGGGAGCTGATGTGGGTCTACTCCTTTGAACAAAACTGGTTTGAGGCCTATCGATACGCCAACCTCCTCAGCAAGGAGAACAAGTGGTCCCAGGCAGTCTATGTATTTCAGAAAGCTGCCATCTTGAGCATGATGCCAGAGGAAGAAGTGAACAAGCTCAATGAAAATGTGGTGGAATTATTCAGGCAGGTGGAGGGCCTCAGGCTGAGATTTGCTGGGAAGTCGATTCCAACGGAGAAGTTTGCAGCGAAGAAGGCCCAGAGATACTCCTCTTCCACCCCCGTGAAACTTGTCGTTCCTGCTTTGGAAATGATGTATGTGTGGAATGGCTTCACAATAGTAGGCAAAAGACCCGAGCTGACTGAAGGAATCCTGTCAACTTTAGAGAAAGCAGAAGAGCAGCTCAGAAATGATCCGAACCCATCAGATTACCACACGGGCGATGAGTGCGTTGTCCAGCTGCTGAAGGGTCTGTGCTTAAGAAGTCTGGGACGGCTGGTCCAGGCTGAGATCTGCTTCAATTATGTAATCTCCAGTGATGGTGGCATCAAGCATGATAGCTATCTGGTGCCCTTTACCATGTATGAGCTTGGCCTATTGCACAAGCAGAAAGGTGAAATCAACAAGGCCATTGCTGTAATGGAAAATGTCATGACGAACTACAAGGACTACAACATGGAGTCAAGGCTGCATTTCCGCATCCATGCAGCACTCAACACCATGGGCTCCTTTGCAGCCAAACTTCCACCAACACGCACGGCAGTTTAAGAGATCCCATTACATGAAACTTGACAGTGTTTACTGTTTCATTGTCTGTTGCCTT--

>TmY5.TM1 T0000032630-R1

---------ATGGAGGGTGAAGACAATTCACTACAACAGGTGGGTTTGAAGCAGCCCAATGAGGATACAATATTGCCTGAAGAATTGCCTAAAATGGGTTTGGAGACAGCATTGAAAGAATGCTTCACTGCCCTCAATTTCTTCCTGAACAACAGGTTTGCCGATGCATTGGCTCTTTTAAAACCCTGGAAGAGTGAAAGTATGTACCATGCGATGGGCTACAGTAGCATTCTGGTGATGCAGGCCGCCATGACTTTTGATGCAAAGGACATGGATGCTGCCATGACATCACTGAGAGAAGCCTTGCAGGTCTGCCAGAGATTTCGGAAGAAAACGGGACTAATGGAAAGCTTGGCTAACCTTTGGTACAGACAACCAGCTGACAATCTGACAGAAGAAGAGATGCATGCAGAGCTGTGCTATGCTGAAGTCCTGCTGCAGAAAGCTGCCCTCACGTTCTTGGATGAAAGCATAATAGGCTTCATCAAAGGAGGGATGAAAATTCGACACAGTTATCAGCTTTACAAGGATTGCCAGGCCATGGAAAAAGTCACAAAGGATGAGGAAAAACAGAGAAGCGCACACATTCATTTTAGGGGTGGGGTCAGCATGGGAATTGGATCATTCAATCTGATGCTGTCTCTGCTTCCGTCCAGAGTCCTTAGACTGATGGAGTTTTTGGGCTTCTCTGGAGACAGGGAAGTGGGTTTGTCAGAGTTGAGAGAGGGAGCAACTACCAACAACCTGCGCTCCATCCTCAGCACCCTCACTCTGCTGATGTTTCAGCTCTACATCACAGTGATACTTGGGACTGCTGATGTAAACCTAAGTGAATGTGAGGCTCTGCTGAAACCCTACTGTGAAAAGTTTCCTAATGGGGCTTTAATGCATTTCTTCAGTGCAAGGATTGCTGTGCTCAAAGGAAACTTCACATTTGCCCAAGAGAAGTTCTTGGCATGYATTGCATYGCAGGAAGAGTGGCGTCAGATTCACCACCTGTGCTACTGGGAGCTGATGTGGGTCTACTCCTTTGAACAAAACTGGTTTGAGGCCTATCGATACGCCAACCTCCTCAGCAAGGAGAACAAGTGGTCCCAGGCAGTCTATGTATTTCAGAAAGCTGCCATCTTGAGCATGATGCCAGAGGAAGAAGTGAACAAGCTCAATGAAAATGTGGTGGAATTATTCAGGCAGGTGGAGGGCCTCAGGCTGAGATTTGCTGGGAAGTCGATTCCAACGGAGAAGTTTGCAGCGAAGAAGGCCCAGAGATACTCCTCTTCCACCMCCGTGAAACTTGTCGTTCCTGCTTTGGAAATGATGTATGTGTGGAATGGCTTCACAATAGTAGGCAAAAGACCCGAGCTGACTGAAGGAATCCTGTCAACTTTAGAGAAAGCAGAAGAGCAGCTCAGAAATGATCCGAACCCATCAGAKTACCACACGGGCGATGAGTGCGTTGTCCAGCTGCTGAAGGGTCTGTGCTTAAGAAGTCTGGGACGGCTGGTCCAGGCTGAGATCTGCTTCAATTATGTAATCTCCAGTGATGGTGGCATCAAGCATGATAGCTATCTGGTGCCCTTTACCATGTATGAGCTTGGCCTATTGCACAAGCAGAAAGGTGAAATCAACAAGGCCATTGCTGTAATGGAAAATGTCATGACGAACTACAAGGACTACAACATGGAGTCAAGGCTGCATTTCCGCATCCATGCAGCACTCAACACCATGGGCTCCTTTGCAGCCAAACTTCCACCAACACGCACGGCAGTTTAAGAGATCCCATTACATGAAACTTGACAGTGTTTACTGTTTCATTGTCTGTTGCCTT--

>TmY6.TM1 T0000032630-R1

---------ATGGAGGGTGAAGACAATTCACTACAACAGGTGGGTTTGAAGCAGCCCAATGAGGATACAATATTGCCTGAAGAATTGCCTAAAATGGGTTTGGAGACAGCATTGAAAGAATGCTTCACTGCCCTCAATTTCTTCCTGAACAACAGGTTTGCCGATGCATTGGCTCTTTTAAAACCCTGGAAGAGTGAAAGTATGTACCATGCGATGGGCTACAGTAGCATTCTGGTGATGCAGGCCGCCATGACTTTTGATGCAAAGGACATGGATGCTGCCATGACATCACTGAGAGAAGCCTTGCAGGTCTGCCAGAGATTTCGGAAGAAAACGGGACTAATGGAAAGCTTGGCTAACCTTTGGTACAGACAACCAGCTGACAATCTGACAGAAGAAGAGATGCATGCAGAGCTGTGCTATGCTGAAGTCCTGCTGCAGAAAGCTGCCCTCACGTTCTTGGATGAAAGCATAATAGGCTTCATCAAAGGAGGGATGAAAATTCGACACAGTTATCAGCTTTACAAGGATTGCCAGGCCATGGAAAAAGTCACAAAGGATGAGGAAAAACAGAGAAGCGCACACATTCATTTTAGGGGTGGGGTCAGCATGGGAATTGGATCATTCAATCTGATGCTGTCTCTGCTTCCGTCCAGAGTCCTTAGACTGATGGAGTTTTTGGGCTTCTCTGGAGACAGGGAAGTGGGTTTGTCAGAGTTGAGAGAGGGAGCAACTACCAACAACCTGCGCTCCATCCTCAGCACCCTCACTCTGCTGATGTTTCAGCTCTACATCACAGTGATACTTGGGACTGCTGATGTAAACCTAAGTGAATGTGAGGCTCTGCTGAAACCCTACTGTGAAAAGTTTCCTAATGGGGCTTTAATGCATTTCTTCAGTGCAAGGATTGCTGTGCTCAAAGGAAACTTCACATTTGCCCAAGAGAAGTTCTTGGCATGYATTGCATYGCAGGAAGAGTGGCGTCAGATTCACCACCTGTGCTACTGGGAGCTGATGTGGGTCTACTCCTTTGAACAAAACTGGTTTGAGGCCTATCGATACGCCAACCTCCTCAGCAAGGAGAACAAGTGGTCCCAGGCAGTCTATGTATTTCAGAAAGCTGCCATCTTGAGCATGATGCCAGAGGAAGAAGTGAACAAGCTCAATGAAAATGTGGTGGAATTATTCAGGCAGGTGGAGGGCCTCAGGCTGAGATTTGCTGGGAAGTCGATTCCAACGGAGAAGTTTGCAGCGAAGAAGGCCCAGAGATACTCCTCTTCCACCMCCGTGAAACTTGTCGTTCCTGCTTTGGAAATGATGTATGTGTGGAATGGCTTCACAATAGTAGGCAAAAGACCCGAGCTGACTGAAGGAATCCTGTCAACTTTAGAGAAAGCAGAAGAGCAGCTCAGAAATGATCCGAACCCATCAGAKTACCACACGGGCGATGAGTGCGTTGTCCAGCTGCTGAAGGGTCTGTGCTTAAGAAGTCTGGGACGGCTGGTCCAGGCTGAGATCTGCTTCAATTATGTAATCTCCAGTGATGGTGGCATCAAGCATGATAGCTATCTGGTGCCCTTTACCATGTATGAGCTTGGCCTATTGCACAAGCAGAAAGGTGAAATCAACAAGGCCATTGCTGTAATGGAAAATGTCATGACGAACTACAAGGACTACAACATGGAGTCAAGGCTGCATTTCCGCATCCATGCAGCACTCAACACCATGGGCTCCTTTGCAGCCAAACTTCCACCAACACGCACGGCAGTTTAAGAGATCCCATTACATGAAACTTGACAGTGTTTACTGTTTCATTGTCT----------
